# Supplementary material for: GaN JBS Diode Device Performance Prediction Method Based on Neural Network
Source: Micromachines (Basel). 2023 Jan 12;14(1):188. doi: 10.3390/mi14010188 (PMC9860762; doi:10.3390/mi14010188)
Supplement: Supplementary file 1 [file micromachines-14-00188-s001.zip › Datasets .pdf]

Sample data composed of 3018 groups of data					
Epidop	L	Impthickness	Impdop	Ron	BV
3.00E+15	0.2	0.15	3.00E+17	0.723	78.094
3.00E+15	0.2	0.15	4.00E+17	0.751	93.869
3.00E+15	0.2	0.15	5.00E+17	0.776	111.348
3.00E+15	0.2	0.15	6.00E+17	0.796	130.679
3.00E+15	0.2	0.15	7.00E+17	0.808	151.773
3.00E+15	0.2	0.15	8.00E+17	0.822	174.481
3.00E+15	0.2	0.15	9.00E+17	0.824	198.540
3.00E+15	0.2	0.15	1.00E+18	0.825	223.205
3.00E+15	0.2	0.2	3.00E+17	0.788	93.877
3.00E+15	0.2	0.2	4.00E+17	0.826	117.867
3.00E+15	0.2	0.2	5.00E+17	0.852	145.013
3.00E+15	0.2	0.2	6.00E+17	0.866	175.605
3.00E+15	0.2	0.2	7.00E+17	0.878	208.305
3.00E+15	0.2	0.2	8.00E+17	0.886	241.584
3.00E+15	0.2	0.2	9.00E+17	0.891	274.783
3.00E+15	0.2	0.2	1.00E+18	0.897	307.976
3.00E+15	0.2	0.25	3.00E+17	0.852	110.876
3.00E+15	0.2	0.25	4.00E+17	0.901	144.166
3.00E+15	0.2	0.25	5.00E+17	0.928	182.411
3.00E+15	0.2	0.25	6.00E+17	0.942	223.434
3.00E+15	0.2	0.25	7.00E+17	0.960	264.700
3.00E+15	0.2	0.25	8.00E+17	0.971	305.956
3.00E+15	0.2	0.25	9.00E+17	0.978	347.335
3.00E+15	0.2	0.25	1.00E+18	0.988	388.657
3.00E+15	0.2	0.3	3.00E+17	0.920	129.042
3.00E+15	0.2	0.3	4.00E+17	0.981	172.470
3.00E+15	0.2	0.3	5.00E+17	1.015	220.869
3.00E+15	0.2	0.3	6.00E+17	1.030	269.655
3.00E+15	0.2	0.3	7.00E+17	1.045	318.553
3.00E+15	0.2	0.3	8.00E+17	1.061	367.738
3.00E+15	0.2	0.3	9.00E+17	1.073	416.484
3.00E+15	0.2	0.3	1.00E+18	1.084	465.619
3.00E+15	0.2	0.35	3.00E+17	0.999	147.901
3.00E+15	0.2	0.35	4.00E+17	1.069	201.582
3.00E+15	0.2	0.35	5.00E+17	1.102	257.959
3.00E+15	0.2	0.35	6.00E+17	1.123	314.275
3.00E+15	0.2	0.35	7.00E+17	1.142	370.712
3.00E+15	0.2	0.35	8.00E+17	1.157	427.077
3.00E+15	0.2	0.35	9.00E+17	1.172	483.480
3.00E+15	0.2	0.35	1.00E+18	1.186	539.793
3.00E+15	0.2	0.4	3.00E+17	1.089	167.720
3.00E+15	0.2	0.4	4.00E+17	1.163	230.604
3.00E+15	0.2	0.4	5.00E+17	1.199	294.079
3.00E+15	0.2	0.4	6.00E+17	1.218	357.672
3.00E+15	0.2	0.4	7.00E+17	1.245	421.173
3.00E+15	0.2	0.4	8.00E+17	1.256	484.685
3.00E+15	0.2	0.4	9.00E+17	1.277	548.394
3.00E+15	0.2	0.4	1.00E+18	1.294	611.640
3.00E+15	0.25	0.15	3.00E+17	0.750	65.258
3.00E+15	0.25	0.15	4.00E+17	0.783	80.240
3.00E+15	0.25	0.15	5.00E+17	0.811	97.263
3.00E+15	0.25	0.15	6.00E+17	0.845	116.227
3.00E+15	0.25	0.15	7.00E+17	0.871	137.285
3.00E+15	0.25	0.15	8.00E+17	0.869	160.231
3.00E+15	0.25	0.15	9.00E+17	0.874	184.899
3.00E+15	0.25	0.15	1.00E+18	0.881	210.667

3.00E+15	0.25	0.2	3.00E+17	0.829	81.161
3.00E+15	0.25	0.2	4.00E+17	0.876	105.229
3.00E+15	0.25	0.2	5.00E+17	0.916	133.121
3.00E+15	0.25	0.2	6.00E+17	0.925	164.664
3.00E+15	0.25	0.2	7.00E+17	0.947	199.279
3.00E+15	0.25	0.2	8.00E+17	0.954	234.897
3.00E+15	0.25	0.2	9.00E+17	0.966	270.455
3.00E+15	0.25	0.2	1.00E+18	0.980	306.017
3.00E+15	0.25	0.25	3.00E+17	0.913	97.929
3.00E+15	0.25	0.25	4.00E+17	0.975	131.685
3.00E+15	0.25	0.25	5.00E+17	1.009	171.197
3.00E+15	0.25	0.25	6.00E+17	1.015	214.504
3.00E+15	0.25	0.25	7.00E+17	1.043	257.679
3.00E+15	0.25	0.25	8.00E+17	1.055	302.791
3.00E+15	0.25	0.25	9.00E+17	1.066	346.716
3.00E+15	0.25	0.25	1.00E+18	1.074	390.776
3.00E+15	0.25	0.3	3.00E+17	1.013	115.960
3.00E+15	0.25	0.3	4.00E+17	1.066	160.504
3.00E+15	0.25	0.3	5.00E+17	1.109	211.535
3.00E+15	0.25	0.3	6.00E+17	1.118	263.701
3.00E+15	0.25	0.3	7.00E+17	1.133	315.865
3.00E+15	0.25	0.3	8.00E+17	1.161	368.116
3.00E+15	0.25	0.3	9.00E+17	1.168	420.282
3.00E+15	0.25	0.3	1.00E+18	1.177	472.382
3.00E+15	0.25	0.35	3.00E+17	1.092	135.231
3.00E+15	0.25	0.35	4.00E+17	1.171	191.196
3.00E+15	0.25	0.35	5.00E+17	1.203	251.243
3.00E+15	0.25	0.35	6.00E+17	1.224	311.336
3.00E+15	0.25	0.35	7.00E+17	1.254	371.467
3.00E+15	0.25	0.35	8.00E+17	1.270	431.216
3.00E+15	0.25	0.35	9.00E+17	1.279	491.325
3.00E+15	0.25	0.35	1.00E+18	1.299	551.279
3.00E+15	0.25	0.4	3.00E+17	1.204	155.882
3.00E+15	0.25	0.4	4.00E+17	1.290	222.293
3.00E+15	0.25	0.4	5.00E+17	1.326	289.972
3.00E+15	0.25	0.4	6.00E+17	1.346	357.850
3.00E+15	0.25	0.4	7.00E+17	1.369	425.628
3.00E+15	0.25	0.4	8.00E+17	1.403	493.351
3.00E+15	0.25	0.4	9.00E+17	1.421	561.039
3.00E+15	0.25	0.4	1.00E+18	1.447	628.612
3.00E+15	0.3	0.15	3.00E+17	0.770	70.066
3.00E+15	0.3	0.15	4.00E+17	0.807	87.681
3.00E+15	0.3	0.15	5.00E+17	0.864	107.877
3.00E+15	0.3	0.15	6.00E+17	0.899	130.568
3.00E+15	0.3	0.15	7.00E+17	0.913	155.729
3.00E+15	0.3	0.15	8.00E+17	0.933	183.124
3.00E+15	0.3	0.15	9.00E+17	0.942	211.824
3.00E+15	0.3	0.15	1.00E+18	0.955	240.758
3.00E+15	0.3	0.2	3.00E+17	0.870	88.669
3.00E+15	0.3	0.2	4.00E+17	0.953	117.196
3.00E+15	0.3	0.2	5.00E+17	0.987	150.394
3.00E+15	0.3	0.2	6.00E+17	1.006	187.795
3.00E+15	0.3	0.2	7.00E+17	1.021	230.656
3.00E+15	0.3	0.2	8.00E+17	1.038	266.832
3.00E+15	0.3	0.2	9.00E+17	1.057	306.344
3.00E+15	0.3	0.2	1.00E+18	1.070	346.056
3.00E+15	0.3	0.25	3.00E+17	0.985	108.647
3.00E+15	0.3	0.25	4.00E+17	1.066	148.970

3.00E+15	0.3	0.25	5.00E+17	1.096	198.981
3.00E+15	0.3	0.25	6.00E+17	1.113	244.650
3.00E+15	0.3	0.25	7.00E+17	1.139	297.477
3.00E+15	0.3	0.25	8.00E+17	1.158	342.773
3.00E+15	0.3	0.25	9.00E+17	1.166	391.806
3.00E+15	0.3	0.25	1.00E+18	1.178	445.348
3.00E+15	0.3	0.3	3.00E+17	1.100	133.092
3.00E+15	0.3	0.3	4.00E+17	1.180	186.985
3.00E+15	0.3	0.3	5.00E+17	1.208	245.409
3.00E+15	0.3	0.3	6.00E+17	1.233	300.109
3.00E+15	0.3	0.3	7.00E+17	1.264	362.391
3.00E+15	0.3	0.3	8.00E+17	1.277	404.125
3.00E+15	0.3	0.3	9.00E+17	1.289	445.322
3.00E+15	0.3	0.3	1.00E+18	1.299	537.702
3.00E+15	0.3	0.35	3.00E+17	1.222	154.168
3.00E+15	0.3	0.35	4.00E+17	1.309	219.770
3.00E+15	0.3	0.35	5.00E+17	1.344	286.808
3.00E+15	0.3	0.35	6.00E+17	1.371	358.174
3.00E+15	0.3	0.35	7.00E+17	1.393	425.612
3.00E+15	0.3	0.35	8.00E+17	1.420	492.993
3.00E+15	0.3	0.35	9.00E+17	1.436	555.480
3.00E+15	0.3	0.35	1.00E+18	1.462	627.795
3.00E+15	0.3	0.4	3.00E+17	1.345	182.483
3.00E+15	0.3	0.4	4.00E+17	1.441	254.972
3.00E+15	0.3	0.4	5.00E+17	1.486	334.832
3.00E+15	0.3	0.4	6.00E+17	1.527	411.086
3.00E+15	0.3	0.4	7.00E+17	1.551	482.684
3.00E+15	0.3	0.4	8.00E+17	1.583	563.505
3.00E+15	0.3	0.4	9.00E+17	1.619	639.651
3.00E+15	0.3	0.4	1.00E+18	1.627	715.196
3.00E+15	0.35	0.15	3.00E+17	0.796	75.992
3.00E+15	0.35	0.15	4.00E+17	0.846	96.491
3.00E+15	0.35	0.15	5.00E+17	0.914	120.024
3.00E+15	0.35	0.15	6.00E+17	0.956	146.563
3.00E+15	0.35	0.15	7.00E+17	0.981	175.959
3.00E+15	0.35	0.15	8.00E+17	1.002	207.293
3.00E+15	0.35	0.15	9.00E+17	1.021	239.047
3.00E+15	0.35	0.15	1.00E+18	1.033	270.693
3.00E+15	0.35	0.2	3.00E+17	0.925	97.944
3.00E+15	0.35	0.2	4.00E+17	1.024	131.333
3.00E+15	0.35	0.2	5.00E+17	1.056	170.271
3.00E+15	0.35	0.2	6.00E+17	1.080	213.162
3.00E+15	0.35	0.2	7.00E+17	1.107	256.624
3.00E+15	0.35	0.2	8.00E+17	1.129	300.073
3.00E+15	0.35	0.2	9.00E+17	1.142	343.498
3.00E+15	0.35	0.2	1.00E+18	1.154	387.069
3.00E+15	0.35	0.25	3.00E+17	1.039	121.818
3.00E+15	0.35	0.25	4.00E+17	1.140	169.313
3.00E+15	0.35	0.25	5.00E+17	1.180	222.709
3.00E+15	0.35	0.25	6.00E+17	1.218	276.566
3.00E+15	0.35	0.25	7.00E+17	1.247	330.634
3.00E+15	0.35	0.25	8.00E+17	1.266	384.685
3.00E+15	0.35	0.25	9.00E+17	1.269	438.462
3.00E+15	0.35	0.25	1.00E+18	1.281	492.048
3.00E+15	0.35	0.3	3.00E+17	1.188	148.051
3.00E+15	0.35	0.3	4.00E+17	1.286	210.052
3.00E+15	0.35	0.3	5.00E+17	1.321	274.211
3.00E+15	0.35	0.3	6.00E+17	1.358	338.421

3.00E+15	0.35	0.3	7.00E+17	1.391	402.594
3.00E+15	0.35	0.3	8.00E+17	1.402	466.774
3.00E+15	0.35	0.3	9.00E+17	1.421	530.862
3.00E+15	0.35	0.3	1.00E+18	1.445	593.629
3.00E+15	0.35	0.35	3.00E+17	1.334	176.360
3.00E+15	0.35	0.35	4.00E+17	1.437	250.232
3.00E+15	0.35	0.35	5.00E+17	1.495	324.204
3.00E+15	0.35	0.35	6.00E+17	1.528	398.267
3.00E+15	0.35	0.35	7.00E+17	1.551	472.532
3.00E+15	0.35	0.35	8.00E+17	1.588	546.480
3.00E+15	0.35	0.35	9.00E+17	1.620	620.531
3.00E+15	0.35	0.35	1.00E+18	1.638	694.493
3.00E+15	0.35	0.4	3.00E+17	1.497	205.689
3.00E+15	0.35	0.4	4.00E+17	1.612	289.477
3.00E+15	0.35	0.4	5.00E+17	1.677	373.356
3.00E+15	0.35	0.4	6.00E+17	1.718	457.307
3.00E+15	0.35	0.4	7.00E+17	1.758	541.155
3.00E+15	0.35	0.4	8.00E+17	1.800	625.017
3.00E+15	0.35	0.4	9.00E+17	1.832	708.593
3.00E+15	0.35	0.4	1.00E+18	1.843	791.839
3.00E+15	0.4	0.15	3.00E+17	0.819	80.126
3.00E+15	0.4	0.15	4.00E+17	0.886	103.098
3.00E+15	0.4	0.15	5.00E+17	0.972	129.651
3.00E+15	0.4	0.15	6.00E+17	1.028	159.658
3.00E+15	0.4	0.15	7.00E+17	1.065	192.663
3.00E+15	0.4	0.15	8.00E+17	1.089	226.829
3.00E+15	0.4	0.15	9.00E+17	1.103	260.903
3.00E+15	0.4	0.15	1.00E+18	1.112	294.691
3.00E+15	0.4	0.2	3.00E+17	0.989	104.776
3.00E+15	0.4	0.2	4.00E+17	1.092	142.385
3.00E+15	0.4	0.2	5.00E+17	1.145	186.243
3.00E+15	0.4	0.2	6.00E+17	1.183	232.929
3.00E+15	0.4	0.2	7.00E+17	1.212	279.662
3.00E+15	0.4	0.2	8.00E+17	1.225	326.527
3.00E+15	0.4	0.2	9.00E+17	1.246	373.351
3.00E+15	0.4	0.2	1.00E+18	1.259	420.041
3.00E+15	0.4	0.25	3.00E+17	1.167	131.951
3.00E+15	0.4	0.25	4.00E+17	1.254	185.632
3.00E+15	0.4	0.25	5.00E+17	1.302	243.832
3.00E+15	0.4	0.25	6.00E+17	1.345	302.048
3.00E+15	0.4	0.25	7.00E+17	1.365	360.152
3.00E+15	0.4	0.25	8.00E+17	1.388	418.526
3.00E+15	0.4	0.25	9.00E+17	1.404	476.613
3.00E+15	0.4	0.25	1.00E+18	1.417	534.859
3.00E+15	0.4	0.3	3.00E+17	1.338	162.049
3.00E+15	0.4	0.3	4.00E+17	1.407	230.595
3.00E+15	0.4	0.3	5.00E+17	1.485	299.675
3.00E+15	0.4	0.3	6.00E+17	1.507	369.300
3.00E+15	0.4	0.3	7.00E+17	1.526	438.571
3.00E+15	0.4	0.3	8.00E+17	1.557	507.861
3.00E+15	0.4	0.3	9.00E+17	1.595	577.164
3.00E+15	0.4	0.3	1.00E+18	1.619	646.503
3.00E+15	0.4	0.35	3.00E+17	1.493	194.319
3.00E+15	0.4	0.35	4.00E+17	1.583	274.570
3.00E+15	0.4	0.35	5.00E+17	1.678	354.824
3.00E+15	0.4	0.35	6.00E+17	1.697	435.021
3.00E+15	0.4	0.35	7.00E+17	1.738	515.254
3.00E+15	0.4	0.35	8.00E+17	1.783	595.412

3.00E+15	0.4	0.35	9.00E+17	1.806	675.456
3.00E+15	0.4	0.35	1.00E+18	1.832	755.372
3.00E+15	0.4	0.4	3.00E+17	1.674	226.881
3.00E+15	0.4	0.4	4.00E+17	1.798	317.811
3.00E+15	0.4	0.4	5.00E+17	1.899	408.660
3.00E+15	0.4	0.4	6.00E+17	1.934	499.562
3.00E+15	0.4	0.4	7.00E+17	1.999	590.359
3.00E+15	0.4	0.4	8.00E+17	2.030	681.135
3.00E+15	0.4	0.4	9.00E+17	2.068	771.649
3.00E+15	0.4	0.4	1.00E+18	2.122	861.666
3.00E+15	0.45	0.15	3.00E+17	0.841	84.522
3.00E+15	0.45	0.15	4.00E+17	0.925	110.166
3.00E+15	0.45	0.15	5.00E+17	1.029	139.828
3.00E+15	0.45	0.15	6.00E+17	1.095	173.472
3.00E+15	0.45	0.15	7.00E+17	1.147	209.707
3.00E+15	0.45	0.15	8.00E+17	1.163	246.336
3.00E+15	0.45	0.15	9.00E+17	1.182	282.901
3.00E+15	0.45	0.15	1.00E+18	1.195	319.598
3.00E+15	0.45	0.2	3.00E+17	1.054	112.218
3.00E+15	0.45	0.2	4.00E+17	1.170	154.453
3.00E+15	0.45	0.2	5.00E+17	1.240	203.048
3.00E+15	0.45	0.2	6.00E+17	1.284	253.156
3.00E+15	0.45	0.2	7.00E+17	1.310	303.344
3.00E+15	0.45	0.2	8.00E+17	1.334	353.475
3.00E+15	0.45	0.2	9.00E+17	1.368	403.622
3.00E+15	0.45	0.2	1.00E+18	1.383	453.681
3.00E+15	0.45	0.25	3.00E+17	1.263	142.896
3.00E+15	0.45	0.25	4.00E+17	1.364	202.575
3.00E+15	0.45	0.25	5.00E+17	1.441	264.967
3.00E+15	0.45	0.25	6.00E+17	1.466	327.427
3.00E+15	0.45	0.25	7.00E+17	1.515	389.793
3.00E+15	0.45	0.25	8.00E+17	1.547	452.275
3.00E+15	0.45	0.25	9.00E+17	1.569	514.596
3.00E+15	0.45	0.25	1.00E+18	1.592	576.952
3.00E+15	0.45	0.3	3.00E+17	1.455	176.949
3.00E+15	0.45	0.3	4.00E+17	1.562	251.108
3.00E+15	0.45	0.3	5.00E+17	1.655	325.541
3.00E+15	0.45	0.3	6.00E+17	1.692	399.982
3.00E+15	0.45	0.3	7.00E+17	1.729	474.234
3.00E+15	0.45	0.3	8.00E+17	1.773	548.583
3.00E+15	0.45	0.3	9.00E+17	1.812	623.021
3.00E+15	0.45	0.3	1.00E+18	1.833	697.344
3.00E+15	0.45	0.35	3.00E+17	1.664	212.492
3.00E+15	0.45	0.35	4.00E+17	1.797	298.609
3.00E+15	0.45	0.35	5.00E+17	1.909	384.707
3.00E+15	0.45	0.35	6.00E+17	1.948	470.755
3.00E+15	0.45	0.35	7.00E+17	2.012	527.283
3.00E+15	0.45	0.35	8.00E+17	2.055	642.916
3.00E+15	0.45	0.35	9.00E+17	2.094	728.836
3.00E+15	0.45	0.35	1.00E+18	2.129	814.579
3.00E+15	0.45	0.4	3.00E+17	1.900	247.562
3.00E+15	0.45	0.4	4.00E+17	2.075	345.298
3.00E+15	0.45	0.4	5.00E+17	2.217	443.064
3.00E+15	0.45	0.4	6.00E+17	2.252	540.655
3.00E+15	0.45	0.4	7.00E+17	2.334	638.239
3.00E+15	0.45	0.4	8.00E+17	2.397	735.878
3.00E+15	0.45	0.4	9.00E+17	2.458	833.177
3.00E+15	0.45	0.4	1.00E+18	2.523	929.550

3.00E+15	0.5	0.15	3.00E+17	0.866	187.510
3.00E+15	0.5	0.15	4.00E+17	0.957	225.432
3.00E+15	0.5	0.15	5.00E+17	1.087	250.644
3.00E+15	0.5	0.15	6.00E+17	1.174	268.855
3.00E+15	0.5	0.15	7.00E+17	1.239	364.250
3.00E+15	0.5	0.15	8.00E+17	1.261	407.956
3.00E+15	0.5	0.15	9.00E+17	1.293	420.863
3.00E+15	0.5	0.15	1.00E+18	1.312	495.827
3.00E+15	0.5	0.2	3.00E+17	1.109	238.043
3.00E+15	0.5	0.2	4.00E+17	1.257	297.789
3.00E+15	0.5	0.2	5.00E+17	1.369	357.959
3.00E+15	0.5	0.2	6.00E+17	1.407	417.121
3.00E+15	0.5	0.2	7.00E+17	1.453	477.739
3.00E+15	0.5	0.2	8.00E+17	1.479	537.170
3.00E+15	0.5	0.2	9.00E+17	1.505	597.186
3.00E+15	0.5	0.2	1.00E+18	1.524	657.226
3.00E+15	0.5	0.25	3.00E+17	1.362	285.067
3.00E+15	0.5	0.25	4.00E+17	1.493	360.253
3.00E+15	0.5	0.25	5.00E+17	1.612	435.454
3.00E+15	0.5	0.25	6.00E+17	1.653	511.416
3.00E+15	0.5	0.25	7.00E+17	1.697	586.565
3.00E+15	0.5	0.25	8.00E+17	1.727	662.222
3.00E+15	0.5	0.25	9.00E+17	1.766	737.264
3.00E+15	0.5	0.25	1.00E+18	1.799	811.957
3.00E+15	0.5	0.3	3.00E+17	1.605	308.308
3.00E+15	0.5	0.3	4.00E+17	1.742	420.627
3.00E+15	0.5	0.3	5.00E+17	1.896	511.526
3.00E+15	0.5	0.3	6.00E+17	1.923	601.975
3.00E+15	0.5	0.3	7.00E+17	1.978	534.049
3.00E+15	0.5	0.3	8.00E+17	2.039	782.572
3.00E+15	0.5	0.3	9.00E+17	2.087	871.778
3.00E+15	0.5	0.3	1.00E+18	2.117	959.612
3.00E+15	0.5	0.35	3.00E+17	1.878	374.727
3.00E+15	0.5	0.35	4.00E+17	2.053	479.815
3.00E+15	0.5	0.35	5.00E+17	2.249	585.136
3.00E+15	0.5	0.35	6.00E+17	2.269	690.445
3.00E+15	0.5	0.35	7.00E+17	2.356	794.921
3.00E+15	0.5	0.4	3.00E+17	2.195	418.292
3.00E+15	0.5	0.4	4.00E+17	2.422	537.741
3.00E+15	0.5	0.4	5.00E+17	2.666	657.195
3.00E+15	0.5	0.4	6.00E+17	2.708	776.619
3.00E+15	0.5	0.4	7.00E+17	2.792	894.610
3.00E+15	0.6	0.15	3.00E+17	0.919	199.380
3.00E+15	0.6	0.15	4.00E+17	1.011	246.624
3.00E+15	0.6	0.15	5.00E+17	1.221	293.680
3.00E+15	0.6	0.15	6.00E+17	1.376	336.399
3.00E+15	0.6	0.15	7.00E+17	1.476	370.067
3.00E+15	0.6	0.15	8.00E+17	1.544	390.304
3.00E+15	0.6	0.15	9.00E+17	1.570	408.676
3.00E+15	0.6	0.15	1.00E+18	1.601	426.697
3.00E+15	0.6	0.2	3.00E+17	1.263	252.455
3.00E+15	0.6	0.2	4.00E+17	1.489	316.829
3.00E+15	0.6	0.2	5.00E+17	1.672	381.252
3.00E+15	0.6	0.2	6.00E+17	1.766	446.240
3.00E+15	0.6	0.2	7.00E+17	1.817	459.681
3.00E+15	0.6	0.2	8.00E+17	1.872	480.117
3.00E+15	0.6	0.2	9.00E+17	1.915	506.748
3.00E+15	0.6	0.2	1.00E+18	1.959	530.208

3.00E+15	0.6	0.25	3.00E+17	1.679	303.976
3.00E+15	0.6	0.25	4.00E+17	1.895	385.190
3.00E+15	0.6	0.25	5.00E+17	2.113	467.030
3.00E+15	0.6	0.25	6.00E+17	2.134	549.008
3.00E+15	0.6	0.25	7.00E+17	2.228	630.187
3.00E+15	0.6	0.25	8.00E+17	2.298	711.966
3.00E+15	0.6	0.3	3.00E+17	2.072	354.690
3.00E+15	0.6	0.3	4.00E+17	2.343	444.536
3.00E+15	0.6	0.3	5.00E+17	2.595	550.674
3.00E+15	0.6	0.3	6.00E+17	2.697	648.603
3.00E+15	0.6	0.3	7.00E+17	2.813	746.983
3.00E+15	0.6	0.3	8.00E+17	2.918	844.623
3.00E+15	0.6	0.35	3.00E+17	2.553	403.007
3.00E+15	0.6	0.35	4.00E+17	2.904	518.030
3.00E+15	0.6	0.35	5.00E+17	3.306	599.039
3.00E+15	0.6	0.35	6.00E+17	3.428	746.434
3.00E+15	0.6	0.35	7.00E+17	3.554	860.172
3.00E+15	0.6	0.35	8.00E+17	3.670	972.689
3.00E+15	0.6	0.4	3.00E+17	3.150	451.424
3.00E+15	0.6	0.4	4.00E+17	3.665	581.712
5.00E+15	0.2	0.15	3.00E+17	0.445	63.032
5.00E+15	0.2	0.15	4.00E+17	0.457	73.613
5.00E+15	0.2	0.15	5.00E+17	0.469	84.628
5.00E+15	0.2	0.15	6.00E+17	0.478	96.283
5.00E+15	0.2	0.15	7.00E+17	0.485	109.063
5.00E+15	0.2	0.15	8.00E+17	0.490	122.927
5.00E+15	0.2	0.15	9.00E+17	0.493	137.907
5.00E+15	0.2	0.15	1.00E+18	0.498	154.091
5.00E+15	0.2	0.2	3.00E+17	0.480	73.506
5.00E+15	0.2	0.2	4.00E+17	0.493	88.427
5.00E+15	0.2	0.2	5.00E+17	0.505	104.852
5.00E+15	0.2	0.2	6.00E+17	0.512	123.104
5.00E+15	0.2	0.2	7.00E+17	0.517	142.775
5.00E+15	0.2	0.2	8.00E+17	0.521	161.783
5.00E+15	0.2	0.2	9.00E+17	0.525	191.296
5.00E+15	0.2	0.2	1.00E+18	0.528	217.400
5.00E+15	0.2	0.25	3.00E+17	0.508	84.080
5.00E+15	0.2	0.25	4.00E+17	0.522	104.093
5.00E+15	0.2	0.25	5.00E+17	0.535	127.347
5.00E+15	0.2	0.25	6.00E+17	0.544	153.829
5.00E+15	0.2	0.25	7.00E+17	0.548	183.157
5.00E+15	0.2	0.25	8.00E+17	0.556	215.859
5.00E+15	0.2	0.25	9.00E+17	0.559	251.315
5.00E+15	0.2	0.25	1.00E+18	0.563	287.259
5.00E+15	0.2	0.3	3.00E+17	0.535	94.675
5.00E+15	0.2	0.3	4.00E+17	0.556	121.016
5.00E+15	0.2	0.3	5.00E+17	0.576	151.830
5.00E+15	0.2	0.3	6.00E+17	0.582	187.124
5.00E+15	0.2	0.3	7.00E+17	0.589	226.439
5.00E+15	0.2	0.3	8.00E+17	0.598	269.922
5.00E+15	0.2	0.3	9.00E+17	0.604	314.415
5.00E+15	0.2	0.3	1.00E+18	0.608	365.844
5.00E+15	0.2	0.35	3.00E+17	0.564	105.728
5.00E+15	0.2	0.35	4.00E+17	0.591	138.743
5.00E+15	0.2	0.35	5.00E+17	0.613	177.616
5.00E+15	0.2	0.35	6.00E+17	0.626	205.440
5.00E+15	0.2	0.35	7.00E+17	0.635	272.512
5.00E+15	0.2	0.35	8.00E+17	0.642	327.458

5.00E+15	0.2	0.35	9.00E+17	0.650	383.517
5.00E+15	0.2	0.35	1.00E+18	0.655	439.612
5.00E+15	0.2	0.4	3.00E+17	0.599	117.472
5.00E+15	0.2	0.4	4.00E+17	0.639	157.767
5.00E+15	0.2	0.4	5.00E+17	0.659	205.666
5.00E+15	0.2	0.4	6.00E+17	0.669	258.366
5.00E+15	0.2	0.4	7.00E+17	0.681	321.865
5.00E+15	0.2	0.4	8.00E+17	0.688	385.078
5.00E+15	0.2	0.4	9.00E+17	0.695	448.370
5.00E+15	0.2	0.4	1.00E+18	0.705	511.836
5.00E+15	0.25	0.15	3.00E+17	0.458	53.554
5.00E+15	0.25	0.15	4.00E+17	0.474	64.090
5.00E+15	0.25	0.15	5.00E+17	0.489	75.076
5.00E+15	0.25	0.15	6.00E+17	0.500	86.647
5.00E+15	0.25	0.15	7.00E+17	0.509	99.266
5.00E+15	0.25	0.15	8.00E+17	0.513	113.100
5.00E+15	0.25	0.15	9.00E+17	0.518	128.324
5.00E+15	0.25	0.15	1.00E+18	0.523	144.648
5.00E+15	0.25	0.2	3.00E+17	0.493	64.639
5.00E+15	0.25	0.2	4.00E+17	0.519	79.869
5.00E+15	0.25	0.2	5.00E+17	0.535	96.588
5.00E+15	0.25	0.2	6.00E+17	0.551	115.679
5.00E+15	0.25	0.2	7.00E+17	0.552	137.087
5.00E+15	0.25	0.2	8.00E+17	0.557	160.865
5.00E+15	0.25	0.2	9.00E+17	0.563	186.927
5.00E+15	0.25	0.2	1.00E+18	0.566	215.145
5.00E+15	0.25	0.25	3.00E+17	0.541	75.159
5.00E+15	0.25	0.25	4.00E+17	0.568	95.474
5.00E+15	0.25	0.25	5.00E+17	0.582	119.308
5.00E+15	0.25	0.25	6.00E+17	0.587	146.814
5.00E+15	0.25	0.25	7.00E+17	0.596	177.732
5.00E+15	0.25	0.25	8.00E+17	0.608	212.227
5.00E+15	0.25	0.25	9.00E+17	0.615	226.627
5.00E+15	0.25	0.25	1.00E+18	0.623	247.421
5.00E+15	0.25	0.3	3.00E+17	0.579	85.725
5.00E+15	0.25	0.3	4.00E+17	0.609	112.459
5.00E+15	0.25	0.3	5.00E+17	0.630	144.405
5.00E+15	0.25	0.3	6.00E+17	0.633	181.245
5.00E+15	0.25	0.3	7.00E+17	0.652	222.983
5.00E+15	0.25	0.3	8.00E+17	0.651	269.528
5.00E+15	0.25	0.3	9.00E+17	0.664	320.317
5.00E+15	0.25	0.3	1.00E+18	0.667	372.432
5.00E+15	0.25	0.35	3.00E+17	0.613	96.844
5.00E+15	0.25	0.35	4.00E+17	0.657	130.871
5.00E+15	0.25	0.35	5.00E+17	0.675	171.640
5.00E+15	0.25	0.35	6.00E+17	0.686	218.994
5.00E+15	0.25	0.35	7.00E+17	0.701	272.763
5.00E+15	0.25	0.35	8.00E+17	0.710	331.708
5.00E+15	0.25	0.35	9.00E+17	0.721	391.681
5.00E+15	0.25	0.35	1.00E+18	0.728	451.718
5.00E+15	0.25	0.4	3.00E+17	0.657	108.874
5.00E+15	0.25	0.4	4.00E+17	0.709	150.927
5.00E+15	0.25	0.4	5.00E+17	0.723	201.351
5.00E+15	0.25	0.4	6.00E+17	0.745	260.095
5.00E+15	0.25	0.4	7.00E+17	0.760	326.020
5.00E+15	0.25	0.4	8.00E+17	0.770	378.293
5.00E+15	0.25	0.4	9.00E+17	0.783	461.321
5.00E+15	0.25	0.4	1.00E+18	0.789	528.884

5.00E+15	0.3	0.15	3.00E+17	0.472	56.818
5.00E+15	0.3	0.15	4.00E+17	0.493	68.678
5.00E+15	0.3	0.15	5.00E+17	0.513	81.192
5.00E+15	0.3	0.15	6.00E+17	0.528	94.786
5.00E+15	0.3	0.15	7.00E+17	0.539	109.893
5.00E+15	0.3	0.15	8.00E+17	0.548	126.764
5.00E+15	0.3	0.15	9.00E+17	0.556	144.987
5.00E+15	0.3	0.15	1.00E+18	0.563	164.758
5.00E+15	0.3	0.2	3.00E+17	0.521	69.245
5.00E+15	0.3	0.2	4.00E+17	0.556	86.582
5.00E+15	0.3	0.2	5.00E+17	0.578	106.436
5.00E+15	0.3	0.2	6.00E+17	0.582	129.413
5.00E+15	0.3	0.2	7.00E+17	0.592	155.264
5.00E+15	0.3	0.2	8.00E+17	0.606	183.826
5.00E+15	0.3	0.2	9.00E+17	0.616	215.157
5.00E+15	0.3	0.2	1.00E+18	0.617	249.317
5.00E+15	0.3	0.25	3.00E+17	0.571	82.631
5.00E+15	0.3	0.25	4.00E+17	0.618	107.025
5.00E+15	0.3	0.25	5.00E+17	0.630	134.036
5.00E+15	0.3	0.25	6.00E+17	0.642	169.749
5.00E+15	0.3	0.25	7.00E+17	0.656	204.656
5.00E+15	0.3	0.25	8.00E+17	0.670	250.007
5.00E+15	0.3	0.25	9.00E+17	0.673	292.209
5.00E+15	0.3	0.25	1.00E+18	0.679	340.709
5.00E+15	0.3	0.3	3.00E+17	0.621	94.079
5.00E+15	0.3	0.3	4.00E+17	0.673	126.346
5.00E+15	0.3	0.3	5.00E+17	0.691	167.563
5.00E+15	0.3	0.3	6.00E+17	0.707	209.635
5.00E+15	0.3	0.3	7.00E+17	0.725	264.292
5.00E+15	0.3	0.3	8.00E+17	0.731	316.806
5.00E+15	0.3	0.3	9.00E+17	0.737	379.499
5.00E+15	0.3	0.3	1.00E+18	0.748	433.028
5.00E+15	0.3	0.35	3.00E+17	0.677	107.805
5.00E+15	0.3	0.35	4.00E+17	0.736	149.199
5.00E+15	0.3	0.35	5.00E+17	0.757	198.814
5.00E+15	0.3	0.35	6.00E+17	0.774	256.632
5.00E+15	0.3	0.35	7.00E+17	0.792	326.011
5.00E+15	0.3	0.35	8.00E+17	0.801	388.596
5.00E+15	0.3	0.35	9.00E+17	0.813	460.751
5.00E+15	0.3	0.35	1.00E+18	0.823	527.976
5.00E+15	0.3	0.4	3.00E+17	0.740	122.740
5.00E+15	0.3	0.4	4.00E+17	0.805	176.656
5.00E+15	0.3	0.4	5.00E+17	0.826	239.303
5.00E+15	0.3	0.4	6.00E+17	0.848	311.712
5.00E+15	0.3	0.4	7.00E+17	0.866	383.137
5.00E+15	0.3	0.4	8.00E+17	0.880	463.926
5.00E+15	0.3	0.4	9.00E+17	0.896	534.580
5.00E+15	0.3	0.4	1.00E+18	0.908	615.786
5.00E+15	0.35	0.15	3.00E+17	0.490	60.740
5.00E+15	0.35	0.15	4.00E+17	0.515	73.914
5.00E+15	0.35	0.15	5.00E+17	0.541	88.060
5.00E+15	0.35	0.15	6.00E+17	0.564	103.878
5.00E+15	0.35	0.15	7.00E+17	0.579	121.911
5.00E+15	0.35	0.15	8.00E+17	0.591	141.553
5.00E+15	0.35	0.15	9.00E+17	0.599	163.140
5.00E+15	0.35	0.15	1.00E+18	0.607	186.581
5.00E+15	0.35	0.2	3.00E+17	0.548	74.689
5.00E+15	0.35	0.2	4.00E+17	0.601	94.672

5.00E+15	0.35	0.2	5.00E+17	0.624	118.212
5.00E+15	0.35	0.2	6.00E+17	0.631	145.330
5.00E+15	0.35	0.2	7.00E+17	0.650	175.822
5.00E+15	0.35	0.2	8.00E+17	0.658	209.753
5.00E+15	0.35	0.2	9.00E+17	0.666	247.042
5.00E+15	0.35	0.2	1.00E+18	0.665	287.491
5.00E+15	0.35	0.25	3.00E+17	0.614	88.771
5.00E+15	0.35	0.25	4.00E+17	0.670	117.380
5.00E+15	0.35	0.25	5.00E+17	0.684	151.571
5.00E+15	0.35	0.25	6.00E+17	0.703	190.847
5.00E+15	0.35	0.25	7.00E+17	0.728	235.618
5.00E+15	0.35	0.25	8.00E+17	0.735	285.344
5.00E+15	0.35	0.25	9.00E+17	0.747	338.688
5.00E+15	0.35	0.25	1.00E+18	0.752	392.490
5.00E+15	0.35	0.3	3.00E+17	0.686	104.087
5.00E+15	0.35	0.3	4.00E+17	0.746	142.688
5.00E+15	0.35	0.3	5.00E+17	0.763	188.780
5.00E+15	0.35	0.3	6.00E+17	0.785	242.560
5.00E+15	0.35	0.3	7.00E+17	0.801	303.162
5.00E+15	0.35	0.3	8.00E+17	0.815	367.152
5.00E+15	0.35	0.3	9.00E+17	0.817	431.280
5.00E+15	0.35	0.3	1.00E+18	0.827	495.388
5.00E+15	0.35	0.35	3.00E+17	0.766	120.981
5.00E+15	0.35	0.35	4.00E+17	0.823	170.426
5.00E+15	0.35	0.35	5.00E+17	0.851	230.054
5.00E+15	0.35	0.35	6.00E+17	0.880	299.346
5.00E+15	0.35	0.35	7.00E+17	0.887	373.263
5.00E+15	0.35	0.35	8.00E+17	0.904	447.152
5.00E+15	0.35	0.35	9.00E+17	0.920	521.035
5.00E+15	0.35	0.35	1.00E+18	0.931	594.977
5.00E+15	0.35	0.4	3.00E+17	0.840	139.198
5.00E+15	0.35	0.4	4.00E+17	0.912	200.677
5.00E+15	0.35	0.4	5.00E+17	0.938	275.115
5.00E+15	0.35	0.4	6.00E+17	0.966	358.240
5.00E+15	0.35	0.4	7.00E+17	0.979	441.982
5.00E+15	0.35	0.4	8.00E+17	0.998	525.654
5.00E+15	0.35	0.4	9.00E+17	1.021	609.305
5.00E+15	0.35	0.4	1.00E+18	1.032	690.417
5.00E+15	0.4	0.15	3.00E+17	0.507	63.251
5.00E+15	0.4	0.15	4.00E+17	0.537	77.537
5.00E+15	0.4	0.15	5.00E+17	0.577	93.406
5.00E+15	0.4	0.15	6.00E+17	0.608	111.484
5.00E+15	0.4	0.15	7.00E+17	0.628	131.746
5.00E+15	0.4	0.15	8.00E+17	0.644	154.340
5.00E+15	0.4	0.15	9.00E+17	0.648	178.860
5.00E+15	0.4	0.15	1.00E+18	0.659	205.590
5.00E+15	0.4	0.2	3.00E+17	0.584	78.415
5.00E+15	0.4	0.2	4.00E+17	0.651	100.782
5.00E+15	0.4	0.2	5.00E+17	0.676	127.599
5.00E+15	0.4	0.2	6.00E+17	0.698	158.345
5.00E+15	0.4	0.2	7.00E+17	0.716	193.123
5.00E+15	0.4	0.2	8.00E+17	0.728	231.765
5.00E+15	0.4	0.2	9.00E+17	0.738	274.310
5.00E+15	0.4	0.2	1.00E+18	0.740	320.072
5.00E+15	0.4	0.25	3.00E+17	0.682	94.392
5.00E+15	0.4	0.25	4.00E+17	0.740	126.972
5.00E+15	0.4	0.25	5.00E+17	0.764	165.930
5.00E+15	0.4	0.25	6.00E+17	0.781	211.074

5.00E+15	0.4	0.25	7.00E+17	0.803	262.183
5.00E+15	0.4	0.25	8.00E+17	0.813	318.769
5.00E+15	0.4	0.25	9.00E+17	0.826	376.889
5.00E+15	0.4	0.25	1.00E+18	0.833	434.985
5.00E+15	0.4	0.3	3.00E+17	0.771	112.314
5.00E+15	0.4	0.3	4.00E+17	0.831	156.301
5.00E+15	0.4	0.3	5.00E+17	0.858	209.277
5.00E+15	0.4	0.3	6.00E+17	0.888	270.917
5.00E+15	0.4	0.3	7.00E+17	0.902	339.284
5.00E+15	0.4	0.3	8.00E+17	0.913	346.261
5.00E+15	0.4	0.3	9.00E+17	0.928	382.496
5.00E+15	0.4	0.3	1.00E+18	0.938	547.011
5.00E+15	0.4	0.35	3.00E+17	0.859	131.781
5.00E+15	0.4	0.35	4.00E+17	0.918	188.693
5.00E+15	0.4	0.35	5.00E+17	0.955	257.356
5.00E+15	0.4	0.35	6.00E+17	0.982	335.929
5.00E+15	0.4	0.35	7.00E+17	0.998	416.059
5.00E+15	0.4	0.35	8.00E+17	1.017	496.201
5.00E+15	0.4	0.35	9.00E+17	1.036	576.220
5.00E+15	0.4	0.35	1.00E+18	1.049	656.190
5.00E+15	0.4	0.4	3.00E+17	0.951	153.125
5.00E+15	0.4	0.4	4.00E+17	1.022	224.199
5.00E+15	0.4	0.4	5.00E+17	1.063	309.887
5.00E+15	0.4	0.4	6.00E+17	1.091	400.674
5.00E+15	0.4	0.4	7.00E+17	1.120	491.484
5.00E+15	0.4	0.4	8.00E+17	1.144	582.330
5.00E+15	0.4	0.4	9.00E+17	1.161	672.900
5.00E+15	0.4	0.4	1.00E+18	1.182	762.845
5.00E+15	0.45	0.15	3.00E+17	0.527	65.758
5.00E+15	0.45	0.15	4.00E+17	0.563	81.291
5.00E+15	0.45	0.15	5.00E+17	0.617	98.984
5.00E+15	0.45	0.15	6.00E+17	0.657	119.491
5.00E+15	0.45	0.15	7.00E+17	0.685	142.305
5.00E+15	0.45	0.15	8.00E+17	0.698	167.661
5.00E+15	0.45	0.15	9.00E+17	0.710	195.392
5.00E+15	0.45	0.15	1.00E+18	0.718	225.506
5.00E+15	0.45	0.2	3.00E+17	0.627	82.474
5.00E+15	0.45	0.2	4.00E+17	0.698	107.678
5.00E+15	0.45	0.2	5.00E+17	0.735	137.767
5.00E+15	0.45	0.2	6.00E+17	0.759	172.620
5.00E+15	0.45	0.2	7.00E+17	0.779	211.919
5.00E+15	0.45	0.2	8.00E+17	0.794	255.798
5.00E+15	0.45	0.2	9.00E+17	0.815	303.786
5.00E+15	0.45	0.2	1.00E+18	0.823	353.781
5.00E+15	0.45	0.25	3.00E+17	0.747	100.485
5.00E+15	0.45	0.25	4.00E+17	0.808	137.279
5.00E+15	0.45	0.25	5.00E+17	0.848	181.408
5.00E+15	0.45	0.25	6.00E+17	0.871	232.629
5.00E+15	0.45	0.25	7.00E+17	0.895	290.647
5.00E+15	0.45	0.25	8.00E+17	0.915	352.621
5.00E+15	0.45	0.25	9.00E+17	0.920	414.767
5.00E+15	0.45	0.25	1.00E+18	0.938	477.218
5.00E+15	0.45	0.3	3.00E+17	0.848	120.947
5.00E+15	0.45	0.3	4.00E+17	0.913	170.869
5.00E+15	0.45	0.3	5.00E+17	0.947	231.054
5.00E+15	0.45	0.3	6.00E+17	0.975	300.811
5.00E+15	0.45	0.3	7.00E+17	1.010	375.113
5.00E+15	0.45	0.3	8.00E+17	1.032	449.487

5.00E+15	0.45	0.3	9.00E+17	1.050	523.825
5.00E+15	0.45	0.3	1.00E+18	1.067	596.840
5.00E+15	0.45	0.35	3.00E+17	0.958	143.347
5.00E+15	0.45	0.35	4.00E+17	1.038	207.919
5.00E+15	0.45	0.35	5.00E+17	1.092	286.253
5.00E+15	0.45	0.35	6.00E+17	1.120	371.850
5.00E+15	0.45	0.35	7.00E+17	1.144	457.891
5.00E+15	0.45	0.35	8.00E+17	1.174	543.932
5.00E+15	0.45	0.35	9.00E+17	1.200	629.860
5.00E+15	0.45	0.35	1.00E+18	1.217	715.575
5.00E+15	0.45	0.4	3.00E+17	1.076	167.714
5.00E+15	0.45	0.4	4.00E+17	1.172	248.765
5.00E+15	0.45	0.4	5.00E+17	1.239	344.358
5.00E+15	0.45	0.4	6.00E+17	1.270	441.902
5.00E+15	0.45	0.4	7.00E+17	1.304	535.484
5.00E+15	0.45	0.4	8.00E+17	1.346	637.054
5.00E+15	0.45	0.4	9.00E+17	1.370	734.285
5.00E+15	0.45	0.4	1.00E+18	1.395	830.724
5.00E+15	0.5	0.15	3.00E+17	0.547	129.890
5.00E+15	0.5	0.15	4.00E+17	0.590	158.517
5.00E+15	0.5	0.15	5.00E+17	0.658	170.221
5.00E+15	0.5	0.15	6.00E+17	0.710	181.604
5.00E+15	0.5	0.15	7.00E+17	0.746	194.900
5.00E+15	0.5	0.15	8.00E+17	0.764	208.139
5.00E+15	0.5	0.15	9.00E+17	0.776	224.442
5.00E+15	0.5	0.15	1.00E+18	0.790	236.387
5.00E+15	0.5	0.2	3.00E+17	0.669	162.195
5.00E+15	0.5	0.2	4.00E+17	0.756	209.067
5.00E+15	0.5	0.2	5.00E+17	0.815	260.872
5.00E+15	0.5	0.2	6.00E+17	0.845	318.410
5.00E+15	0.5	0.2	7.00E+17	0.871	376.054
5.00E+15	0.5	0.2	8.00E+17	0.886	438.130
5.00E+15	0.5	0.2	9.00E+17	0.897	497.743
5.00E+15	0.5	0.2	1.00E+18	0.910	557.771
5.00E+15	0.5	0.25	3.00E+17	0.794	197.357
5.00E+15	0.5	0.25	4.00E+17	0.900	261.544
5.00E+15	0.5	0.25	5.00E+17	0.940	334.944
5.00E+15	0.5	0.25	6.00E+17	0.981	410.228
5.00E+15	0.5	0.25	7.00E+17	1.003	487.633
5.00E+15	0.5	0.25	8.00E+17	1.027	560.931
5.00E+15	0.5	0.25	9.00E+17	1.040	638.461
5.00E+15	0.5	0.25	1.00E+18	1.062	712.853
5.00E+15	0.5	0.3	3.00E+17	0.952	235.278
5.00E+15	0.5	0.3	4.00E+17	1.033	322.938
5.00E+15	0.5	0.3	5.00E+17	1.092	304.658
5.00E+15	0.5	0.3	6.00E+17	1.126	503.457
5.00E+15	0.5	0.3	7.00E+17	1.161	593.844
5.00E+15	0.5	0.3	8.00E+17	1.175	684.128
5.00E+15	0.5	0.3	9.00E+17	1.219	773.394
5.00E+15	0.5	0.3	1.00E+18	1.233	861.228
5.00E+15	0.5	0.35	3.00E+17	1.092	275.927
5.00E+15	0.5	0.35	4.00E+17	1.181	379.944
5.00E+15	0.5	0.35	5.00E+17	1.273	486.922
5.00E+15	0.5	0.35	6.00E+17	1.300	589.928
5.00E+15	0.5	0.35	7.00E+17	1.333	696.934
5.00E+15	0.5	0.35	8.00E+17	1.373	800.541
5.00E+15	0.5	0.35	9.00E+17	1.401	902.373
5.00E+15	0.5	0.35	1.00E+18	1.429	1001.000

5.00E+15	0.5	0.4	3.00E+17	1.238	318.879
5.00E+15	0.5	0.4	4.00E+17	1.351	427.758
5.00E+15	0.5	0.4	5.00E+17	1.477	557.321
5.00E+15	0.5	0.4	6.00E+17	1.502	678.896
5.00E+15	0.5	0.4	7.00E+17	1.551	797.024
5.00E+15	0.5	0.4	8.00E+17	1.606	913.031
5.00E+15	0.5	0.4	9.00E+17	1.629	1025.440
5.00E+15	0.5	0.4	1.00E+18	1.667	1131.870
5.00E+15	0.6	0.15	3.00E+17	0.591	136.917
5.00E+15	0.6	0.15	4.00E+17	0.646	167.845
5.00E+15	0.6	0.15	5.00E+17	0.750	203.410
5.00E+15	0.6	0.15	6.00E+17	0.833	245.321
5.00E+15	0.6	0.15	7.00E+17	0.896	287.248
5.00E+15	0.6	0.15	8.00E+17	0.933	336.102
5.00E+15	0.6	0.2	3.00E+17	0.763	171.839
5.00E+15	0.6	0.2	4.00E+17	0.905	222.625
5.00E+15	0.6	0.2	5.00E+17	1.014	281.161
5.00E+15	0.6	0.2	6.00E+17	1.060	347.241
5.00E+15	0.6	0.2	7.00E+17	1.095	409.508
5.00E+15	0.6	0.2	8.00E+17	1.129	476.233
5.00E+15	0.6	0.2	9.00E+17	1.150	540.840
5.00E+15	0.6	0.2	1.00E+18	1.176	605.547
5.00E+15	0.6	0.25	3.00E+17	1.008	212.295
5.00E+15	0.6	0.25	4.00E+17	1.129	285.555
5.00E+15	0.6	0.25	5.00E+17	1.242	366.585
5.00E+15	0.6	0.25	6.00E+17	1.275	450.191
5.00E+15	0.6	0.25	7.00E+17	1.309	531.524
5.00E+15	0.6	0.25	8.00E+17	1.364	613.284
5.00E+15	0.6	0.25	9.00E+17	1.403	694.654
5.00E+15	0.6	0.25	1.00E+18	1.437	775.312
5.00E+15	0.6	0.3	3.00E+17	1.218	258.489
5.00E+15	0.6	0.3	4.00E+17	1.351	352.459
5.00E+15	0.6	0.3	5.00E+17	1.491	452.563
5.00E+15	0.6	0.3	6.00E+17	1.532	550.478
5.00E+15	0.6	0.3	7.00E+17	1.597	503.521
5.00E+15	0.6	0.3	8.00E+17	1.646	746.440
5.00E+15	0.6	0.3	9.00E+17	1.693	843.016
5.00E+15	0.6	0.3	1.00E+18	1.738	937.728
5.00E+15	0.6	0.35	3.00E+17	1.454	303.850
5.00E+15	0.6	0.35	4.00E+17	1.619	420.125
5.00E+15	0.6	0.35	5.00E+17	1.845	533.920
5.00E+15	0.6	0.35	6.00E+17	1.850	648.647
5.00E+15	0.6	0.35	7.00E+17	1.931	762.548
5.00E+15	0.6	0.35	1.00E+18	2.091	1090.890
5.00E+15	0.6	0.4	3.00E+17	1.732	354.445
5.00E+15	0.6	0.4	4.00E+17	1.938	484.421
5.00E+15	0.6	0.4	5.00E+17	2.283	614.733
5.00E+15	0.6	0.4	6.00E+17	2.248	744.869
5.00E+15	0.6	0.4	7.00E+17	2.356	873.333
5.00E+15	0.6	0.4	8.00E+17	2.433	999.292
5.00E+15	0.6	0.4	9.00E+17	2.518	1119.930
7.00E+15	0.2	0.15	3.00E+17	0.320	55.491
7.00E+15	0.2	0.15	4.00E+17	0.327	64.762
7.00E+15	0.2	0.15	5.00E+17	0.336	74.470
7.00E+15	0.2	0.15	6.00E+17	0.341	84.589
7.00E+15	0.2	0.15	7.00E+17	0.347	95.039
7.00E+15	0.2	0.15	8.00E+17	0.348	105.820
7.00E+15	0.2	0.15	9.00E+17	0.352	116.868

7.00E+15	0.2	0.15	1.00E+18	0.353	128.582
7.00E+15	0.2	0.2	3.00E+17	0.337	64.779
7.00E+15	0.2	0.2	4.00E+17	0.351	77.891
7.00E+15	0.2	0.2	5.00E+17	0.360	91.659
7.00E+15	0.2	0.2	6.00E+17	0.361	106.035
7.00E+15	0.2	0.2	7.00E+17	0.366	121.026
7.00E+15	0.2	0.2	8.00E+17	0.368	130.162
7.00E+15	0.2	0.2	9.00E+17	0.370	142.683
7.00E+15	0.2	0.2	1.00E+18	0.372	150.040
7.00E+15	0.2	0.25	3.00E+17	0.359	73.996
7.00E+15	0.2	0.25	4.00E+17	0.369	90.955
7.00E+15	0.2	0.25	5.00E+17	0.374	108.779
7.00E+15	0.2	0.25	6.00E+17	0.381	127.742
7.00E+15	0.2	0.25	7.00E+17	0.383	147.574
7.00E+15	0.2	0.25	8.00E+17	0.387	172.258
7.00E+15	0.2	0.25	9.00E+17	0.391	197.711
7.00E+15	0.2	0.25	1.00E+18	0.391	223.990
7.00E+15	0.2	0.3	3.00E+17	0.380	83.111
7.00E+15	0.2	0.3	4.00E+17	0.385	103.815
7.00E+15	0.2	0.3	5.00E+17	0.394	125.961
7.00E+15	0.2	0.3	6.00E+17	0.398	151.131
7.00E+15	0.2	0.3	7.00E+17	0.406	179.611
7.00E+15	0.2	0.3	8.00E+17	0.408	210.908
7.00E+15	0.2	0.3	9.00E+17	0.411	243.643
7.00E+15	0.2	0.3	1.00E+18	0.413	282.782
7.00E+15	0.2	0.35	3.00E+17	0.394	91.961
7.00E+15	0.2	0.35	4.00E+17	0.404	116.395
7.00E+15	0.2	0.35	5.00E+17	0.413	144.110
7.00E+15	0.2	0.35	6.00E+17	0.420	174.814
7.00E+15	0.2	0.35	7.00E+17	0.425	210.915
7.00E+15	0.2	0.35	8.00E+17	0.430	200.908
7.00E+15	0.2	0.35	9.00E+17	0.434	295.092
7.00E+15	0.2	0.35	1.00E+18	0.440	343.177
7.00E+15	0.2	0.4	3.00E+17	0.411	100.606
7.00E+15	0.2	0.4	4.00E+17	0.425	129.585
7.00E+15	0.2	0.4	5.00E+17	0.437	163.746
7.00E+15	0.2	0.4	6.00E+17	0.445	203.084
7.00E+15	0.2	0.4	7.00E+17	0.456	247.838
7.00E+15	0.2	0.4	8.00E+17	0.459	297.732
7.00E+15	0.2	0.4	9.00E+17	0.460	350.882
7.00E+15	0.2	0.4	1.00E+18	0.466	410.587
7.00E+15	0.25	0.15	3.00E+17	0.328	47.271
7.00E+15	0.25	0.15	4.00E+17	0.340	56.503
7.00E+15	0.25	0.15	5.00E+17	0.349	66.138
7.00E+15	0.25	0.15	6.00E+17	0.357	76.319
7.00E+15	0.25	0.15	7.00E+17	0.362	86.879
7.00E+15	0.25	0.15	8.00E+17	0.365	97.759
7.00E+15	0.25	0.15	9.00E+17	0.368	109.041
7.00E+15	0.25	0.15	1.00E+18	0.370	120.779
7.00E+15	0.25	0.2	3.00E+17	0.356	57.002
7.00E+15	0.25	0.2	4.00E+17	0.367	70.356
7.00E+15	0.25	0.2	5.00E+17	0.376	84.616
7.00E+15	0.25	0.2	6.00E+17	0.382	99.559
7.00E+15	0.25	0.2	7.00E+17	0.392	115.141
7.00E+15	0.25	0.2	8.00E+17	0.390	132.139
7.00E+15	0.25	0.2	9.00E+17	0.391	150.590
7.00E+15	0.25	0.2	1.00E+18	0.394	171.082
7.00E+15	0.25	0.3	3.00E+17	0.401	75.449

7.00E+15	0.25	0.3	4.00E+17	0.414	96.627
7.00E+15	0.25	0.3	5.00E+17	0.426	119.649
7.00E+15	0.25	0.3	6.00E+17	0.439	145.801
7.00E+15	0.25	0.3	7.00E+17	0.440	175.954
7.00E+15	0.25	0.3	8.00E+17	0.447	209.555
7.00E+15	0.25	0.3	9.00E+17	0.449	246.578
7.00E+15	0.25	0.3	1.00E+18	0.452	287.205
7.00E+15	0.25	0.35	3.00E+17	0.422	84.470
7.00E+15	0.25	0.35	4.00E+17	0.445	109.637
7.00E+15	0.25	0.35	5.00E+17	0.456	138.523
7.00E+15	0.25	0.35	6.00E+17	0.467	172.553
7.00E+15	0.25	0.35	7.00E+17	0.475	211.290
7.00E+15	0.25	0.35	8.00E+17	0.475	254.662
7.00E+15	0.25	0.35	9.00E+17	0.482	302.605
7.00E+15	0.25	0.35	1.00E+18	0.486	355.221
7.00E+15	0.25	0.4	3.00E+17	0.445	93.457
7.00E+15	0.25	0.4	4.00E+17	0.473	123.478
7.00E+15	0.25	0.4	5.00E+17	0.486	159.372
7.00E+15	0.25	0.4	6.00E+17	0.496	201.616
7.00E+15	0.25	0.4	7.00E+17	0.504	249.849
7.00E+15	0.25	0.4	8.00E+17	0.512	304.011
7.00E+15	0.25	0.4	9.00E+17	0.518	363.962
7.00E+15	0.25	0.4	1.00E+18	0.526	429.281
7.00E+15	0.3	0.15	3.00E+17	0.339	50.059
7.00E+15	0.3	0.15	4.00E+17	0.355	60.421
7.00E+15	0.3	0.15	5.00E+17	0.366	71.417
7.00E+15	0.3	0.15	6.00E+17	0.377	82.961
7.00E+15	0.3	0.15	7.00E+17	0.385	94.894
7.00E+15	0.3	0.15	8.00E+17	0.388	107.360
7.00E+15	0.3	0.15	9.00E+17	0.390	120.459
7.00E+15	0.3	0.15	1.00E+18	0.392	134.606
7.00E+15	0.3	0.2	3.00E+17	0.373	60.926
7.00E+15	0.3	0.2	4.00E+17	0.388	76.143
7.00E+15	0.3	0.2	5.00E+17	0.402	92.161
7.00E+15	0.3	0.2	6.00E+17	0.409	109.095
7.00E+15	0.3	0.2	7.00E+17	0.412	127.565
7.00E+15	0.3	0.2	8.00E+17	0.417	147.816
7.00E+15	0.3	0.2	9.00E+17	0.420	170.553
7.00E+15	0.3	0.2	1.00E+18	0.422	195.050
7.00E+15	0.3	0.25	3.00E+17	0.400	72.721
7.00E+15	0.3	0.25	4.00E+17	0.421	92.491
7.00E+15	0.3	0.25	5.00E+17	0.436	112.127
7.00E+15	0.3	0.25	6.00E+17	0.446	135.686
7.00E+15	0.3	0.25	7.00E+17	0.451	162.570
7.00E+15	0.3	0.25	8.00E+17	0.452	195.206
7.00E+15	0.3	0.25	9.00E+17	0.460	225.709
7.00E+15	0.3	0.25	1.00E+18	0.464	261.952
7.00E+15	0.3	0.3	3.00E+17	0.429	83.414
7.00E+15	0.3	0.3	4.00E+17	0.462	107.810
7.00E+15	0.3	0.3	5.00E+17	0.471	135.631
7.00E+15	0.3	0.3	6.00E+17	0.483	165.895
7.00E+15	0.3	0.3	7.00E+17	0.489	205.390
7.00E+15	0.3	0.3	8.00E+17	0.496	243.349
7.00E+15	0.3	0.3	9.00E+17	0.498	292.486
7.00E+15	0.3	0.3	1.00E+18	0.507	338.392
7.00E+15	0.3	0.35	3.00E+17	0.458	93.948
7.00E+15	0.3	0.35	4.00E+17	0.493	123.899
7.00E+15	0.3	0.35	5.00E+17	0.508	159.779

7.00E+15	0.3	0.35	6.00E+17	0.523	201.951
7.00E+15	0.3	0.35	7.00E+17	0.529	246.574
7.00E+15	0.3	0.35	8.00E+17	0.535	303.816
7.00E+15	0.3	0.35	9.00E+17	0.545	358.896
7.00E+15	0.3	0.35	1.00E+18	0.552	425.444
7.00E+15	0.3	0.4	3.00E+17	0.489	104.568
7.00E+15	0.3	0.4	4.00E+17	0.535	139.162
7.00E+15	0.3	0.4	5.00E+17	0.550	183.397
7.00E+15	0.3	0.4	6.00E+17	0.562	235.419
7.00E+15	0.3	0.4	7.00E+17	0.572	298.793
7.00E+15	0.3	0.4	8.00E+17	0.579	361.707
7.00E+15	0.3	0.4	9.00E+17	0.591	440.591
7.00E+15	0.3	0.4	1.00E+18	0.596	510.656
7.00E+15	0.35	0.15	3.00E+17	0.353	53.448
7.00E+15	0.35	0.15	4.00E+17	0.370	64.910
7.00E+15	0.35	0.15	5.00E+17	0.384	77.212
7.00E+15	0.35	0.15	6.00E+17	0.400	89.973
7.00E+15	0.35	0.15	7.00E+17	0.407	103.376
7.00E+15	0.35	0.15	8.00E+17	0.411	117.490
7.00E+15	0.35	0.15	9.00E+17	0.416	132.865
7.00E+15	0.35	0.15	1.00E+18	0.418	149.538
7.00E+15	0.35	0.2	3.00E+17	0.393	65.646
7.00E+15	0.35	0.2	4.00E+17	0.417	82.604
7.00E+15	0.35	0.2	5.00E+17	0.433	100.597
7.00E+15	0.35	0.2	6.00E+17	0.446	119.906
7.00E+15	0.35	0.2	7.00E+17	0.451	141.631
7.00E+15	0.35	0.2	8.00E+17	0.458	166.122
7.00E+15	0.35	0.2	9.00E+17	0.460	192.879
7.00E+15	0.35	0.2	1.00E+18	0.464	222.131
7.00E+15	0.35	0.25	3.00E+17	0.428	77.733
7.00E+15	0.35	0.25	4.00E+17	0.459	99.761
7.00E+15	0.35	0.25	5.00E+17	0.478	124.090
7.00E+15	0.35	0.25	6.00E+17	0.487	152.243
7.00E+15	0.35	0.25	7.00E+17	0.497	184.319
7.00E+15	0.35	0.25	8.00E+17	0.500	218.292
7.00E+15	0.35	0.25	9.00E+17	0.518	242.776
7.00E+15	0.35	0.25	1.00E+18	0.521	303.296
7.00E+15	0.35	0.3	3.00E+17	0.472	89.722
7.00E+15	0.35	0.3	4.00E+17	0.506	117.467
7.00E+15	0.35	0.3	5.00E+17	0.529	150.333
7.00E+15	0.35	0.3	6.00E+17	0.536	188.733
7.00E+15	0.35	0.3	7.00E+17	0.550	232.785
7.00E+15	0.35	0.3	8.00E+17	0.558	282.197
7.00E+15	0.35	0.3	9.00E+17	0.565	336.801
7.00E+15	0.35	0.3	1.00E+18	0.572	396.571
7.00E+15	0.35	0.35	3.00E+17	0.520	101.737
7.00E+15	0.35	0.35	4.00E+17	0.549	136.679
7.00E+15	0.35	0.35	5.00E+17	0.578	179.465
7.00E+15	0.35	0.35	6.00E+17	0.586	229.517
7.00E+15	0.35	0.35	7.00E+17	0.600	286.828
7.00E+15	0.35	0.35	8.00E+17	0.610	351.184
7.00E+15	0.35	0.35	9.00E+17	0.621	422.068
7.00E+15	0.35	0.35	1.00E+18	0.628	495.887
7.00E+15	0.35	0.4	3.00E+17	0.558	114.342
7.00E+15	0.35	0.4	4.00E+17	0.603	158.117
7.00E+15	0.35	0.4	5.00E+17	0.626	211.332
7.00E+15	0.35	0.4	6.00E+17	0.643	274.249
7.00E+15	0.35	0.4	7.00E+17	0.656	346.192

7.00E+15	0.35	0.4	8.00E+17	0.672	426.623
7.00E+15	0.35	0.4	9.00E+17	0.681	510.146
7.00E+15	0.35	0.4	1.00E+18	0.687	593.442
7.00E+15	0.4	0.15	3.00E+17	0.368	55.536
7.00E+15	0.4	0.15	4.00E+17	0.391	68.031
7.00E+15	0.4	0.15	5.00E+17	0.410	81.318
7.00E+15	0.4	0.15	6.00E+17	0.431	95.260
7.00E+15	0.4	0.15	7.00E+17	0.440	109.887
7.00E+15	0.4	0.15	8.00E+17	0.447	125.998
7.00E+15	0.4	0.15	9.00E+17	0.454	143.469
7.00E+15	0.4	0.15	1.00E+18	0.455	162.394
7.00E+15	0.4	0.2	3.00E+17	0.417	68.806
7.00E+15	0.4	0.2	4.00E+17	0.451	87.189
7.00E+15	0.4	0.2	5.00E+17	0.473	106.744
7.00E+15	0.4	0.2	6.00E+17	0.494	128.732
7.00E+15	0.4	0.2	7.00E+17	0.498	153.624
7.00E+15	0.4	0.2	8.00E+17	0.503	181.410
7.00E+15	0.4	0.2	9.00E+17	0.509	212.130
7.00E+15	0.4	0.2	1.00E+18	0.501	245.685
7.00E+15	0.4	0.25	3.00E+17	0.472	82.039
7.00E+15	0.4	0.25	4.00E+17	0.511	106.080
7.00E+15	0.4	0.25	5.00E+17	0.528	133.766
7.00E+15	0.4	0.25	6.00E+17	0.546	166.278
7.00E+15	0.4	0.25	7.00E+17	0.556	203.169
7.00E+15	0.4	0.25	8.00E+17	0.561	244.378
7.00E+15	0.4	0.25	9.00E+17	0.575	289.937
7.00E+15	0.4	0.25	1.00E+18	0.586	339.712
7.00E+15	0.4	0.3	3.00E+17	0.529	95.389
7.00E+15	0.4	0.3	4.00E+17	0.570	126.724
7.00E+15	0.4	0.3	5.00E+17	0.590	164.674
7.00E+15	0.4	0.3	6.00E+17	0.604	208.957
7.00E+15	0.4	0.3	7.00E+17	0.617	259.502
7.00E+15	0.4	0.3	8.00E+17	0.631	316.474
7.00E+15	0.4	0.3	9.00E+17	0.635	379.630
7.00E+15	0.4	0.3	1.00E+18	0.652	447.718
7.00E+15	0.4	0.35	3.00E+17	0.577	108.957
7.00E+15	0.4	0.35	4.00E+17	0.628	149.392
7.00E+15	0.4	0.35	5.00E+17	0.652	198.839
7.00E+15	0.4	0.35	6.00E+17	0.670	256.574
7.00E+15	0.4	0.35	7.00E+17	0.685	323.006
7.00E+15	0.4	0.35	8.00E+17	0.699	397.626
7.00E+15	0.4	0.35	9.00E+17	0.711	477.157
7.00E+15	0.4	0.35	1.00E+18	0.716	556.931
7.00E+15	0.4	0.4	3.00E+17	0.641	123.766
7.00E+15	0.4	0.4	4.00E+17	0.695	174.433
7.00E+15	0.4	0.4	5.00E+17	0.712	236.440
7.00E+15	0.4	0.4	6.00E+17	0.739	309.392
7.00E+15	0.4	0.4	7.00E+17	0.745	393.270
7.00E+15	0.4	0.4	8.00E+17	0.772	483.384
7.00E+15	0.4	0.4	9.00E+17	0.782	573.971
7.00E+15	0.4	0.4	1.00E+18	0.794	663.944
7.00E+15	0.45	0.15	3.00E+17	0.384	57.613
7.00E+15	0.45	0.15	4.00E+17	0.410	71.139
7.00E+15	0.45	0.15	5.00E+17	0.442	85.424
7.00E+15	0.45	0.15	6.00E+17	0.469	100.560
7.00E+15	0.45	0.15	7.00E+17	0.482	116.862
7.00E+15	0.45	0.15	8.00E+17	0.488	134.849
7.00E+15	0.45	0.15	9.00E+17	0.495	154.866

7.00E+15	0.45	0.15	1.00E+18	0.504	176.597
7.00E+15	0.45	0.2	3.00E+17	0.445	72.067
7.00E+15	0.45	0.2	4.00E+17	0.490	91.853
7.00E+15	0.45	0.2	5.00E+17	0.524	113.544
7.00E+15	0.45	0.2	6.00E+17	0.535	138.234
7.00E+15	0.45	0.2	7.00E+17	0.543	166.664
7.00E+15	0.45	0.2	8.00E+17	0.553	198.285
7.00E+15	0.45	0.2	9.00E+17	0.567	233.034
7.00E+15	0.45	0.2	1.00E+18	0.578	271.153
7.00E+15	0.45	0.25	3.00E+17	0.518	86.456
7.00E+15	0.45	0.25	4.00E+17	0.567	113.010
7.00E+15	0.45	0.25	5.00E+17	0.586	144.300
7.00E+15	0.45	0.25	6.00E+17	0.613	181.087
7.00E+15	0.45	0.25	7.00E+17	0.626	223.034
7.00E+15	0.45	0.25	8.00E+17	0.633	270.071
7.00E+15	0.45	0.25	9.00E+17	0.651	322.079
7.00E+15	0.45	0.25	1.00E+18	0.663	379.074
7.00E+15	0.45	0.3	3.00E+17	0.580	101.090
7.00E+15	0.45	0.3	4.00E+17	0.628	136.391
7.00E+15	0.45	0.3	5.00E+17	0.673	179.605
7.00E+15	0.45	0.3	6.00E+17	0.686	230.215
7.00E+15	0.45	0.3	8.00E+17	0.723	352.924
7.00E+15	0.45	0.3	9.00E+17	0.733	424.523
7.00E+15	0.45	0.3	1.00E+18	0.742	498.786
7.00E+15	0.45	0.35	3.00E+17	0.659	116.626
7.00E+15	0.45	0.35	4.00E+17	0.722	162.774
7.00E+15	0.45	0.35	5.00E+17	0.736	218.976
7.00E+15	0.45	0.35	6.00E+17	0.773	285.125
7.00E+15	0.45	0.35	7.00E+17	0.791	360.929
7.00E+15	0.45	0.35	8.00E+17	0.810	445.037
7.00E+15	0.45	0.35	9.00E+17	0.822	530.941
7.00E+15	0.45	0.35	1.00E+18	0.838	616.602
7.00E+15	0.45	0.4	3.00E+17	0.732	133.561
7.00E+15	0.45	0.4	4.00E+17	0.795	191.668
7.00E+15	0.45	0.4	5.00E+17	0.840	262.116
7.00E+15	0.45	0.4	6.00E+17	0.853	346.053
7.00E+15	0.45	0.4	7.00E+17	0.879	440.982
7.00E+15	0.45	0.4	8.00E+17	0.902	538.468
7.00E+15	0.45	0.4	9.00E+17	0.927	536.737
7.00E+15	0.45	0.4	1.00E+18	0.938	577.246
7.00E+15	0.5	0.15	3.00E+17	0.402	108.990
7.00E+15	0.5	0.15	4.00E+17	0.431	129.706
7.00E+15	0.5	0.15	5.00E+17	0.472	153.880
7.00E+15	0.5	0.15	6.00E+17	0.506	178.386
7.00E+15	0.5	0.15	7.00E+17	0.529	151.462
7.00E+15	0.5	0.15	8.00E+17	0.537	160.868
7.00E+15	0.5	0.15	9.00E+17	0.547	173.019
7.00E+15	0.5	0.15	1.00E+18	0.556	183.175
7.00E+15	0.5	0.2	3.00E+17	0.479	132.400
7.00E+15	0.5	0.2	4.00E+17	0.537	166.250
7.00E+15	0.5	0.2	5.00E+17	0.579	203.599
7.00E+15	0.5	0.2	6.00E+17	0.601	245.685
7.00E+15	0.5	0.2	7.00E+17	0.615	292.744
7.00E+15	0.5	0.2	8.00E+17	0.627	343.904
7.00E+15	0.5	0.2	9.00E+17	0.642	399.687
7.00E+15	0.5	0.2	1.00E+18	0.654	458.726
7.00E+15	0.5	0.25	3.00E+17	0.578	158.683
7.00E+15	0.5	0.25	4.00E+17	0.634	206.069

7.00E+15	0.5	0.25	5.00E+17	0.666	259.182
7.00E+15	0.5	0.25	6.00E+17	0.691	316.194
7.00E+15	0.5	0.25	7.00E+17	0.699	390.294
7.00E+15	0.5	0.25	8.00E+17	0.721	464.301
7.00E+15	0.5	0.25	9.00E+17	0.734	539.415
7.00E+15	0.5	0.25	1.00E+18	0.752	614.133
7.00E+15	0.5	0.3	3.00E+17	0.674	186.314
7.00E+15	0.5	0.3	4.00E+17	0.721	249.067
7.00E+15	0.5	0.3	5.00E+17	0.765	322.274
7.00E+15	0.5	0.3	6.00E+17	0.772	405.847
7.00E+15	0.5	0.3	9.00E+17	0.852	674.835
7.00E+15	0.5	0.3	1.00E+18	0.862	763.040
7.00E+15	0.5	0.35	3.00E+17	0.758	215.738
7.00E+15	0.5	0.35	4.00E+17	0.825	295.612
7.00E+15	0.5	0.35	5.00E+17	0.872	323.876
7.00E+15	0.5	0.35	6.00E+17	0.887	493.982
7.00E+15	0.5	0.35	7.00E+17	0.929	598.725
7.00E+15	0.5	0.35	8.00E+17	0.951	702.628
7.00E+15	0.5	0.35	9.00E+17	0.974	804.511
7.00E+15	0.5	0.35	1.00E+18	0.984	903.144
7.00E+15	0.5	0.4	3.00E+17	0.855	247.298
7.00E+15	0.5	0.4	4.00E+17	0.937	346.596
7.00E+15	0.5	0.4	5.00E+17	0.983	462.145
7.00E+15	0.5	0.4	7.00E+17	1.048	699.343
7.00E+15	0.5	0.4	8.00E+17	1.074	815.681
7.00E+15	0.5	0.4	9.00E+17	1.105	928.291
7.00E+15	0.5	0.4	1.00E+18	1.134	1035.480
7.00E+15	0.6	0.15	3.00E+17	0.442	113.417
7.00E+15	0.6	0.15	4.00E+17	0.483	135.216
7.00E+15	0.6	0.15	5.00E+17	0.546	156.245
7.00E+15	0.6	0.15	6.00E+17	0.606	191.453
7.00E+15	0.6	0.15	7.00E+17	0.640	223.346
7.00E+15	0.6	0.15	8.00E+17	0.670	258.361
7.00E+15	0.6	0.15	9.00E+17	0.687	295.876
7.00E+15	0.6	0.15	1.00E+18	0.700	336.493
7.00E+15	0.6	0.2	3.00E+17	0.555	138.634
7.00E+15	0.6	0.2	4.00E+17	0.650	175.368
7.00E+15	0.6	0.2	5.00E+17	0.722	218.992
7.00E+15	0.6	0.2	6.00E+17	0.762	266.884
7.00E+15	0.6	0.2	7.00E+17	0.785	320.228
7.00E+15	0.6	0.2	8.00E+17	0.811	378.983
7.00E+15	0.6	0.2	9.00E+17	0.828	441.810
7.00E+15	0.6	0.2	1.00E+18	0.846	504.262
7.00E+15	0.6	0.25	3.00E+17	0.708	169.095
7.00E+15	0.6	0.25	4.00E+17	0.806	222.243
7.00E+15	0.6	0.25	5.00E+17	0.874	284.156
7.00E+15	0.6	0.25	6.00E+17	0.908	354.881
7.00E+15	0.6	0.25	7.00E+17	0.932	433.474
7.00E+15	0.6	0.25	8.00E+17	0.970	512.510
7.00E+15	0.6	0.25	9.00E+17	0.985	595.969
7.00E+15	0.6	0.25	1.00E+18	1.023	676.677
7.00E+15	0.6	0.3	3.00E+17	0.866	201.337
7.00E+15	0.6	0.3	4.00E+17	0.964	273.122
7.00E+15	0.6	0.3	5.00E+17	1.045	247.581
7.00E+15	0.6	0.3	6.00E+17	1.075	452.786
7.00E+15	0.6	0.3	7.00E+17	1.115	548.515
7.00E+15	0.6	0.3	8.00E+17	1.160	648.567
7.00E+15	0.6	0.3	9.00E+17	1.192	744.974

7.00E+15	0.6	0.35	3.00E+17	1.018	235.497
7.00E+15	0.6	0.35	4.00E+17	1.138	328.066
7.00E+15	0.6	0.35	5.00E+17	1.268	437.028
7.00E+15	0.6	0.35	6.00E+17	1.276	548.509
7.00E+15	0.6	0.35	7.00E+17	1.341	664.805
7.00E+15	0.6	0.35	8.00E+17	1.387	777.288
7.00E+15	0.6	0.35	9.00E+17	1.410	887.690
7.00E+15	0.6	0.35	1.00E+18	1.448	993.941
7.00E+15	0.6	0.4	3.00E+17	1.202	273.043
7.00E+15	0.6	0.4	4.00E+17	1.331	388.210
7.00E+15	0.6	0.4	5.00E+17	1.486	515.320
7.00E+15	0.6	0.4	6.00E+17	1.528	647.521
7.00E+15	0.6	0.4	7.00E+17	1.588	776.196
7.00E+15	0.6	0.4	8.00E+17	1.641	902.299
7.00E+15	0.6	0.4	9.00E+17	1.694	1023.360
7.00E+15	0.6	0.4	1.00E+18	1.744	1136.900
4.00E+15	0.2	0.15	3.00E+17	0.547	68.486
4.00E+15	0.2	0.15	4.00E+17	0.571	80.273
4.00E+15	0.2	0.15	5.00E+17	0.584	93.327
4.00E+15	0.2	0.15	6.00E+17	0.599	108.019
4.00E+15	0.2	0.15	7.00E+17	0.609	123.968
4.00E+15	0.2	0.15	8.00E+17	0.614	141.218
4.00E+15	0.2	0.15	9.00E+17	0.617	159.951
4.00E+15	0.2	0.15	1.00E+18	0.620	179.838
4.00E+15	0.2	0.2	3.00E+17	0.596	80.203
4.00E+15	0.2	0.2	4.00E+17	0.615	98.240
4.00E+15	0.2	0.2	5.00E+17	0.636	118.716
4.00E+15	0.2	0.2	6.00E+17	0.641	141.593
4.00E+15	0.2	0.2	7.00E+17	0.651	167.545
4.00E+15	0.2	0.2	8.00E+17	0.659	195.480
4.00E+15	0.2	0.2	9.00E+17	0.667	225.722
4.00E+15	0.2	0.2	1.00E+18	0.666	258.044
4.00E+15	0.2	0.25	3.00E+17	0.637	92.850
4.00E+15	0.2	0.25	4.00E+17	0.663	117.952
4.00E+15	0.2	0.25	5.00E+17	0.681	147.008
4.00E+15	0.2	0.25	6.00E+17	0.693	179.881
4.00E+15	0.2	0.25	7.00E+17	0.702	216.327
4.00E+15	0.2	0.25	8.00E+17	0.709	256.036
4.00E+15	0.2	0.25	9.00E+17	0.716	297.271
4.00E+15	0.2	0.25	1.00E+18	0.719	338.464
4.00E+15	0.2	0.3	3.00E+17	0.678	106.369
4.00E+15	0.2	0.3	4.00E+17	0.714	139.248
4.00E+15	0.2	0.3	5.00E+17	0.737	177.532
4.00E+15	0.2	0.3	6.00E+17	0.747	221.040
4.00E+15	0.2	0.3	7.00E+17	0.763	268.933
4.00E+15	0.2	0.3	8.00E+17	0.769	317.649
4.00E+15	0.2	0.3	9.00E+17	0.777	366.592
4.00E+15	0.2	0.3	1.00E+18	0.784	415.397
4.00E+15	0.2	0.35	3.00E+17	0.722	120.509
4.00E+15	0.2	0.35	4.00E+17	0.769	161.695
4.00E+15	0.2	0.35	5.00E+17	0.794	209.940
4.00E+15	0.2	0.35	6.00E+17	0.806	264.377
4.00E+15	0.2	0.35	7.00E+17	0.822	320.760
4.00E+15	0.2	0.35	8.00E+17	0.835	376.930
4.00E+15	0.2	0.35	9.00E+17	0.845	433.133
4.00E+15	0.2	0.35	1.00E+18	0.853	489.405
4.00E+15	0.2	0.4	3.00E+17	0.774	135.276
4.00E+15	0.2	0.4	4.00E+17	0.830	185.479

4.00E+15	0.2	0.4	5.00E+17	0.858	244.569
4.00E+15	0.2	0.4	6.00E+17	0.875	307.875
4.00E+15	0.2	0.4	7.00E+17	0.888	371.330
4.00E+15	0.2	0.4	8.00E+17	0.906	434.941
4.00E+15	0.2	0.4	9.00E+17	0.915	498.300
4.00E+15	0.2	0.4	1.00E+18	0.930	561.655
4.00E+15	0.25	0.15	3.00E+17	0.568	58.116
4.00E+15	0.25	0.15	4.00E+17	0.592	69.580
4.00E+15	0.25	0.15	5.00E+17	0.610	82.239
4.00E+15	0.25	0.15	6.00E+17	0.626	96.558
4.00E+15	0.25	0.15	7.00E+17	0.641	112.377
4.00E+15	0.25	0.15	8.00E+17	0.647	129.772
4.00E+15	0.25	0.15	9.00E+17	0.656	148.666
4.00E+15	0.25	0.15	1.00E+18	0.661	168.973
4.00E+15	0.25	0.2	3.00E+17	0.623	70.220
4.00E+15	0.25	0.2	4.00E+17	0.661	88.080
4.00E+15	0.25	0.2	5.00E+17	0.673	109.126
4.00E+15	0.25	0.2	6.00E+17	0.686	133.015
4.00E+15	0.25	0.2	7.00E+17	0.702	159.723
4.00E+15	0.25	0.2	8.00E+17	0.713	189.178
4.00E+15	0.25	0.2	9.00E+17	0.717	221.396
4.00E+15	0.25	0.2	1.00E+18	0.716	255.979
4.00E+15	0.25	0.25	3.00E+17	0.681	82.508
4.00E+15	0.25	0.25	4.00E+17	0.719	107.910
4.00E+15	0.25	0.25	5.00E+17	0.735	137.720
4.00E+15	0.25	0.25	6.00E+17	0.754	171.962
4.00E+15	0.25	0.25	7.00E+17	0.767	210.508
4.00E+15	0.25	0.25	8.00E+17	0.780	243.141
4.00E+15	0.25	0.25	9.00E+17	0.780	270.123
4.00E+15	0.25	0.25	1.00E+18	0.785	340.734
4.00E+15	0.25	0.3	3.00E+17	0.734	95.914
4.00E+15	0.25	0.3	4.00E+17	0.783	129.468
4.00E+15	0.25	0.3	5.00E+17	0.801	169.253
4.00E+15	0.25	0.3	6.00E+17	0.818	215.036
4.00E+15	0.25	0.3	7.00E+17	0.839	265.953
4.00E+15	0.25	0.3	8.00E+17	0.833	318.115
4.00E+15	0.25	0.3	9.00E+17	0.852	370.246
4.00E+15	0.25	0.3	1.00E+18	0.854	422.421
4.00E+15	0.25	0.35	3.00E+17	0.793	110.153
4.00E+15	0.25	0.35	4.00E+17	0.843	152.761
4.00E+15	0.25	0.35	5.00E+17	0.871	203.592
4.00E+15	0.25	0.35	6.00E+17	0.889	261.465
4.00E+15	0.25	0.35	7.00E+17	0.906	321.498
4.00E+15	0.25	0.35	8.00E+17	0.923	381.600
4.00E+15	0.25	0.35	9.00E+17	0.931	441.562
4.00E+15	0.25	0.35	1.00E+18	0.944	501.530
4.00E+15	0.25	0.4	3.00E+17	0.854	125.556
4.00E+15	0.25	0.4	4.00E+17	0.920	177.881
4.00E+15	0.25	0.4	5.00E+17	0.941	240.513
4.00E+15	0.25	0.4	6.00E+17	0.974	307.722
4.00E+15	0.25	0.4	7.00E+17	0.993	375.826
4.00E+15	0.25	0.4	8.00E+17	1.010	443.492
4.00E+15	0.25	0.4	9.00E+17	1.023	511.153
4.00E+15	0.25	0.4	1.00E+18	1.027	578.747
4.00E+15	0.3	0.15	3.00E+17	0.587	61.767
4.00E+15	0.3	0.15	4.00E+17	0.615	74.928
4.00E+15	0.3	0.15	5.00E+17	0.644	89.962
4.00E+15	0.3	0.15	6.00E+17	0.669	107.098

4.00E+15	0.3	0.15	7.00E+17	0.686	126.157
4.00E+15	0.3	0.15	8.00E+17	0.691	146.992
4.00E+15	0.3	0.15	9.00E+17	0.699	169.716
4.00E+15	0.3	0.15	1.00E+18	0.703	194.329
4.00E+15	0.3	0.2	3.00E+17	0.651	75.590
4.00E+15	0.3	0.2	4.00E+17	0.700	96.894
4.00E+15	0.3	0.2	5.00E+17	0.720	121.980
4.00E+15	0.3	0.2	6.00E+17	0.749	152.970
4.00E+15	0.3	0.2	7.00E+17	0.758	182.464
4.00E+15	0.3	0.2	8.00E+17	0.765	217.885
4.00E+15	0.3	0.2	9.00E+17	0.772	256.333
4.00E+15	0.3	0.2	1.00E+18	0.782	299.785
4.00E+15	0.3	0.25	3.00E+17	0.725	90.377
4.00E+15	0.3	0.25	4.00E+17	0.780	120.775
4.00E+15	0.3	0.25	5.00E+17	0.805	156.481
4.00E+15	0.3	0.25	6.00E+17	0.825	197.726
4.00E+15	0.3	0.25	7.00E+17	0.836	247.610
4.00E+15	0.3	0.25	8.00E+17	0.846	292.697
4.00E+15	0.3	0.25	9.00E+17	0.854	345.956
4.00E+15	0.3	0.25	1.00E+18	0.869	395.159
4.00E+15	0.3	0.3	3.00E+17	0.801	108.612
4.00E+15	0.3	0.3	4.00E+17	0.862	149.681
4.00E+15	0.3	0.3	5.00E+17	0.887	198.301
4.00E+15	0.3	0.3	6.00E+17	0.910	250.269
4.00E+15	0.3	0.3	7.00E+17	0.925	312.508
4.00E+15	0.3	0.3	8.00E+17	0.936	366.611
4.00E+15	0.3	0.3	9.00E+17	0.945	424.653
4.00E+15	0.3	0.3	1.00E+18	0.960	487.765
4.00E+15	0.3	0.35	3.00E+17	0.883	124.260
4.00E+15	0.3	0.35	4.00E+17	0.951	175.761
4.00E+15	0.3	0.35	5.00E+17	0.978	237.417
4.00E+15	0.3	0.35	6.00E+17	1.000	308.314
4.00E+15	0.3	0.35	7.00E+17	1.018	371.318
4.00E+15	0.3	0.35	8.00E+17	1.034	443.224
4.00E+15	0.3	0.35	9.00E+17	1.048	505.634
4.00E+15	0.3	0.35	1.00E+18	1.058	577.933
4.00E+15	0.3	0.4	3.00E+17	0.968	143.117
4.00E+15	0.3	0.4	4.00E+17	1.047	210.221
4.00E+15	0.3	0.4	5.00E+17	1.079	285.215
4.00E+15	0.3	0.4	6.00E+17	1.101	361.419
4.00E+15	0.3	0.4	7.00E+17	1.120	432.861
4.00E+15	0.3	0.4	8.00E+17	1.135	513.755
4.00E+15	0.3	0.4	9.00E+17	1.160	584.491
4.00E+15	0.3	0.4	1.00E+18	1.175	665.680
4.00E+15	0.35	0.15	3.00E+17	0.605	66.053
4.00E+15	0.35	0.15	4.00E+17	0.640	81.233
4.00E+15	0.35	0.15	5.00E+17	0.681	98.960
4.00E+15	0.35	0.15	6.00E+17	0.719	119.005
4.00E+15	0.35	0.15	7.00E+17	0.735	141.241
4.00E+15	0.35	0.15	8.00E+17	0.746	165.872
4.00E+15	0.35	0.15	9.00E+17	0.755	192.634
4.00E+15	0.35	0.15	1.00E+18	0.760	221.492
4.00E+15	0.35	0.2	3.00E+17	0.688	82.245
4.00E+15	0.35	0.2	4.00E+17	0.758	107.343
4.00E+15	0.35	0.2	5.00E+17	0.780	136.787
4.00E+15	0.35	0.2	6.00E+17	0.807	170.545
4.00E+15	0.35	0.2	7.00E+17	0.821	208.432
4.00E+15	0.35	0.2	8.00E+17	0.832	250.101

4.00E+15	0.35	0.2	9.00E+17	0.843	293.480
4.00E+15	0.35	0.2	1.00E+18	0.852	336.929
4.00E+15	0.35	0.25	3.00E+17	0.797	100.111
4.00E+15	0.35	0.25	4.00E+17	0.844	135.921
4.00E+15	0.35	0.25	5.00E+17	0.876	178.389
4.00E+15	0.35	0.25	6.00E+17	0.898	227.334
4.00E+15	0.35	0.25	7.00E+17	0.911	280.705
4.00E+15	0.35	0.25	8.00E+17	0.925	334.558
4.00E+15	0.35	0.25	9.00E+17	0.946	388.468
4.00E+15	0.35	0.25	1.00E+18	0.959	442.493
4.00E+15	0.35	0.3	3.00E+17	0.881	119.698
4.00E+15	0.35	0.3	4.00E+17	0.949	167.711
4.00E+15	0.35	0.3	5.00E+17	0.983	225.074
4.00E+15	0.35	0.3	6.00E+17	0.987	288.658
4.00E+15	0.35	0.3	7.00E+17	1.009	352.755
4.00E+15	0.35	0.3	8.00E+17	1.038	416.919
4.00E+15	0.35	0.3	9.00E+17	1.052	480.992
4.00E+15	0.35	0.3	1.00E+18	1.065	545.188
4.00E+15	0.35	0.35	3.00E+17	0.983	140.877
4.00E+15	0.35	0.35	4.00E+17	1.053	202.554
4.00E+15	0.35	0.35	5.00E+17	1.094	274.796
4.00E+15	0.35	0.35	6.00E+17	1.117	348.813
4.00E+15	0.35	0.35	7.00E+17	1.134	422.635
4.00E+15	0.35	0.35	8.00E+17	1.160	496.754
4.00E+15	0.35	0.35	9.00E+17	1.174	570.865
4.00E+15	0.35	0.35	1.00E+18	1.188	644.751
4.00E+15	0.35	0.4	3.00E+17	1.090	163.849
4.00E+15	0.35	0.4	4.00E+17	1.150	240.174
4.00E+15	0.35	0.4	5.00E+17	1.208	323.860
4.00E+15	0.35	0.4	6.00E+17	1.239	407.737
4.00E+15	0.35	0.4	7.00E+17	1.272	491.533
4.00E+15	0.35	0.4	8.00E+17	1.294	575.263
4.00E+15	0.35	0.4	9.00E+17	1.320	658.979
4.00E+15	0.35	0.4	1.00E+18	1.342	742.260
4.00E+15	0.4	0.15	3.00E+17	0.626	68.933
4.00E+15	0.4	0.15	4.00E+17	0.669	85.929
4.00E+15	0.4	0.15	5.00E+17	0.728	105.926
4.00E+15	0.4	0.15	6.00E+17	0.769	128.617
4.00E+15	0.4	0.15	7.00E+17	0.790	153.951
4.00E+15	0.4	0.15	8.00E+17	0.801	181.835
4.00E+15	0.4	0.15	9.00E+17	0.808	212.392
4.00E+15	0.4	0.15	1.00E+18	0.825	244.788
4.00E+15	0.4	0.2	3.00E+17	0.734	87.164
4.00E+15	0.4	0.2	4.00E+17	0.816	115.484
4.00E+15	0.4	0.2	5.00E+17	0.856	148.790
4.00E+15	0.4	0.2	6.00E+17	0.878	187.095
4.00E+15	0.4	0.2	7.00E+17	0.901	230.238
4.00E+15	0.4	0.2	8.00E+17	0.913	276.575
4.00E+15	0.4	0.2	9.00E+17	0.925	323.272
4.00E+15	0.4	0.2	1.00E+18	0.943	369.961
4.00E+15	0.4	0.25	3.00E+17	0.866	107.508
4.00E+15	0.4	0.25	4.00E+17	0.937	148.275
4.00E+15	0.4	0.25	5.00E+17	0.970	196.804
4.00E+15	0.4	0.25	6.00E+17	0.989	252.222
4.00E+15	0.4	0.25	7.00E+17	1.012	310.404
4.00E+15	0.4	0.25	8.00E+17	1.029	368.583
4.00E+15	0.4	0.25	9.00E+17	1.050	426.761
4.00E+15	0.4	0.25	1.00E+18	1.068	484.880

4.00E+15	0.4	0.3	3.00E+17	0.986	130.024
4.00E+15	0.4	0.3	4.00E+17	1.051	184.993
4.00E+15	0.4	0.3	5.00E+17	1.092	250.322
4.00E+15	0.4	0.3	6.00E+17	1.116	319.621
4.00E+15	0.4	0.3	7.00E+17	1.142	358.271
4.00E+15	0.4	0.3	8.00E+17	1.162	458.112
4.00E+15	0.4	0.3	9.00E+17	1.172	527.532
4.00E+15	0.4	0.3	1.00E+18	1.189	596.727
4.00E+15	0.4	0.35	3.00E+17	1.095	154.750
4.00E+15	0.4	0.35	4.00E+17	1.172	225.528
4.00E+15	0.4	0.35	5.00E+17	1.215	305.252
4.00E+15	0.4	0.35	6.00E+17	1.242	385.423
4.00E+15	0.4	0.35	7.00E+17	1.275	465.648
4.00E+15	0.4	0.35	8.00E+17	1.295	545.847
4.00E+15	0.4	0.35	9.00E+17	1.321	625.906
4.00E+15	0.4	0.35	1.00E+18	1.336	705.753
4.00E+15	0.4	0.4	3.00E+17	1.226	181.572
4.00E+15	0.4	0.4	4.00E+17	1.314	268.426
4.00E+15	0.4	0.4	5.00E+17	1.366	359.196
4.00E+15	0.4	0.4	6.00E+17	1.408	450.143
4.00E+15	0.4	0.4	7.00E+17	1.438	540.943
4.00E+15	0.4	0.4	8.00E+17	1.472	631.851
4.00E+15	0.4	0.4	9.00E+17	1.496	722.111
4.00E+15	0.4	0.4	1.00E+18	1.530	812.114
4.00E+15	0.45	0.15	3.00E+17	0.649	71.983
4.00E+15	0.45	0.15	4.00E+17	0.700	91.105
4.00E+15	0.45	0.15	5.00E+17	0.775	113.386
4.00E+15	0.45	0.15	6.00E+17	0.825	138.859
4.00E+15	0.45	0.15	7.00E+17	0.858	167.391
4.00E+15	0.45	0.15	8.00E+17	0.870	198.919
4.00E+15	0.45	0.15	9.00E+17	0.880	233.305
4.00E+15	0.45	0.15	1.00E+18	0.895	269.550
4.00E+15	0.45	0.2	3.00E+17	0.783	92.596
4.00E+15	0.45	0.2	4.00E+17	0.880	124.341
4.00E+15	0.45	0.2	5.00E+17	0.907	161.935
4.00E+15	0.45	0.2	6.00E+17	0.956	205.125
4.00E+15	0.45	0.2	7.00E+17	0.971	253.435
4.00E+15	0.45	0.2	8.00E+17	0.995	303.524
4.00E+15	0.45	0.2	9.00E+17	1.017	353.544
4.00E+15	0.45	0.2	1.00E+18	1.034	403.745
4.00E+15	0.45	0.25	3.00E+17	0.926	115.547
4.00E+15	0.45	0.25	4.00E+17	1.023	161.471
4.00E+15	0.45	0.25	5.00E+17	1.057	216.197
4.00E+15	0.45	0.25	6.00E+17	1.099	277.727
4.00E+15	0.45	0.25	7.00E+17	1.107	339.992
4.00E+15	0.45	0.25	8.00E+17	1.145	402.347
4.00E+15	0.45	0.25	9.00E+17	1.167	464.719
4.00E+15	0.45	0.25	1.00E+18	1.185	527.057
4.00E+15	0.45	0.3	3.00E+17	1.068	141.192
4.00E+15	0.45	0.3	4.00E+17	1.165	203.343
4.00E+15	0.45	0.3	5.00E+17	1.206	276.013
4.00E+15	0.45	0.3	6.00E+17	1.240	350.398
4.00E+15	0.45	0.3	7.00E+17	1.291	424.667
4.00E+15	0.45	0.3	8.00E+17	1.314	499.015
4.00E+15	0.45	0.3	9.00E+17	1.331	573.394
4.00E+15	0.45	0.3	1.00E+18	1.345	647.570
4.00E+15	0.45	0.35	3.00E+17	1.215	169.268
4.00E+15	0.45	0.35	4.00E+17	1.322	249.236

4.00E+15	0.45	0.35	5.00E+17	1.393	335.258
4.00E+15	0.45	0.35	6.00E+17	1.427	421.325
4.00E+15	0.45	0.35	7.00E+17	1.474	507.446
4.00E+15	0.45	0.35	8.00E+17	1.501	593.421
4.00E+15	0.45	0.35	9.00E+17	1.523	568.886
4.00E+15	0.45	0.35	1.00E+18	1.558	765.080
4.00E+15	0.45	0.4	3.00E+17	1.383	200.031
4.00E+15	0.45	0.4	4.00E+17	1.511	295.996
4.00E+15	0.45	0.4	5.00E+17	1.602	393.670
4.00E+15	0.45	0.4	6.00E+17	1.650	491.226
4.00E+15	0.45	0.4	7.00E+17	1.684	588.903
4.00E+15	0.45	0.4	8.00E+17	1.715	686.339
4.00E+15	0.45	0.4	9.00E+17	1.776	783.745
4.00E+15	0.45	0.4	1.00E+18	1.814	878.669
4.00E+15	0.5	0.15	3.00E+17	0.670	150.519
4.00E+15	0.5	0.15	4.00E+17	0.728	180.825
4.00E+15	0.5	0.15	5.00E+17	0.825	200.088
4.00E+15	0.5	0.15	6.00E+17	0.885	219.830
4.00E+15	0.5	0.15	7.00E+17	0.928	232.942
4.00E+15	0.5	0.15	8.00E+17	0.947	249.588
4.00E+15	0.5	0.15	9.00E+17	0.966	402.380
4.00E+15	0.5	0.15	1.00E+18	0.984	446.119
4.00E+15	0.5	0.2	3.00E+17	0.826	190.383
4.00E+15	0.5	0.2	4.00E+17	0.947	246.374
4.00E+15	0.5	0.2	5.00E+17	1.025	305.903
4.00E+15	0.5	0.2	6.00E+17	1.050	367.977
4.00E+15	0.5	0.2	7.00E+17	1.084	427.655
4.00E+15	0.5	0.2	8.00E+17	1.104	487.716
4.00E+15	0.5	0.2	9.00E+17	1.128	547.439
4.00E+15	0.5	0.2	1.00E+18	1.150	607.415
4.00E+15	0.5	0.25	3.00E+17	1.009	236.246
4.00E+15	0.5	0.25	4.00E+17	1.128	308.914
4.00E+15	0.5	0.25	5.00E+17	1.185	386.394
4.00E+15	0.5	0.25	6.00E+17	1.223	461.528
4.00E+15	0.5	0.25	7.00E+17	1.263	537.128
4.00E+15	0.5	0.25	8.00E+17	1.299	611.997
4.00E+15	0.5	0.25	9.00E+17	1.322	687.745
4.00E+15	0.5	0.25	1.00E+18	1.354	762.409
4.00E+15	0.5	0.3	3.00E+17	1.205	279.639
4.00E+15	0.5	0.3	4.00E+17	1.300	307.467
4.00E+15	0.5	0.3	5.00E+17	1.385	462.531
4.00E+15	0.5	0.3	6.00E+17	1.407	552.675
4.00E+15	0.5	0.3	7.00E+17	1.470	643.275
4.00E+15	0.5	0.3	8.00E+17	1.504	733.219
4.00E+15	0.5	0.35	3.00E+17	1.372	323.862
4.00E+15	0.5	0.35	4.00E+17	1.503	430.836
4.00E+15	0.5	0.35	5.00E+17	1.613	536.210
4.00E+15	0.5	0.35	6.00E+17	1.658	641.040
4.00E+15	0.5	0.35	7.00E+17	1.710	746.073
4.00E+15	0.5	0.35	8.00E+17	1.741	849.622
4.00E+15	0.5	0.35	9.00E+17	1.778	951.377
4.00E+15	0.5	0.4	3.00E+17	1.593	369.671
4.00E+15	0.5	0.4	4.00E+17	1.744	488.852
4.00E+15	0.5	0.4	5.00E+17	1.910	608.359
4.00E+15	0.5	0.4	6.00E+17	1.942	727.699
4.00E+15	0.5	0.4	7.00E+17	2.002	845.900
4.00E+15	0.5	0.4	8.00E+17	2.047	961.737
4.00E+15	0.5	0.4	9.00E+17	2.104	1074.390

4.00E+15	0.5	0.4	1.00E+18	2.171	1178.570
4.00E+15	0.6	0.15	3.00E+17	0.717	159.669
4.00E+15	0.6	0.15	4.00E+17	0.784	197.747
4.00E+15	0.6	0.15	5.00E+17	0.932	242.243
4.00E+15	0.6	0.15	6.00E+17	1.035	289.226
4.00E+15	0.6	0.15	7.00E+17	1.106	317.337
4.00E+15	0.6	0.15	8.00E+17	1.157	339.628
4.00E+15	0.6	0.15	9.00E+17	1.184	357.172
4.00E+15	0.6	0.15	1.00E+18	1.213	372.624
4.00E+15	0.6	0.2	3.00E+17	0.949	203.220
4.00E+15	0.6	0.2	4.00E+17	1.129	265.312
4.00E+15	0.6	0.2	5.00E+17	1.263	332.161
4.00E+15	0.6	0.2	6.00E+17	1.315	396.623
4.00E+15	0.6	0.2	7.00E+17	1.359	461.493
4.00E+15	0.6	0.2	8.00E+17	1.408	525.855
4.00E+15	0.6	0.2	9.00E+17	1.452	590.593
4.00E+15	0.6	0.25	3.00E+17	1.249	252.651
4.00E+15	0.6	0.25	4.00E+17	1.420	336.473
4.00E+15	0.6	0.25	5.00E+17	1.563	417.657
4.00E+15	0.6	0.25	6.00E+17	1.586	499.249
4.00E+15	0.6	0.25	7.00E+17	1.658	580.857
4.00E+15	0.6	0.25	8.00E+17	1.727	662.479
4.00E+15	0.6	0.25	9.00E+17	1.765	743.940
4.00E+15	0.6	0.25	1.00E+18	1.801	824.596
4.00E+15	0.6	0.3	3.00E+17	1.534	303.272
4.00E+15	0.6	0.3	4.00E+17	1.717	403.384
4.00E+15	0.6	0.3	5.00E+17	1.878	501.664
4.00E+15	0.6	0.3	7.00E+17	2.036	697.916
4.00E+15	0.6	0.3	8.00E+17	2.099	795.282
4.00E+15	0.6	0.3	9.00E+17	2.144	892.508
4.00E+15	0.6	0.3	1.00E+18	2.180	986.931
4.00E+15	0.6	0.35	3.00E+17	1.845	354.686
4.00E+15	0.6	0.35	4.00E+17	2.075	468.652
4.00E+15	0.6	0.35	5.00E+17	2.372	582.929
4.00E+15	0.6	0.35	6.00E+17	2.401	697.271
4.00E+15	0.6	0.35	7.00E+17	2.494	673.538
4.00E+15	0.6	0.35	8.00E+17	2.570	924.294
4.00E+15	0.6	0.35	9.00E+17	2.660	1033.760
4.00E+15	0.6	0.35	1.00E+18	2.753	1139.480
4.00E+15	0.6	0.4	3.00E+17	2.225	402.986
4.00E+15	0.6	0.4	4.00E+17	2.531	533.182
4.00E+15	0.6	0.4	5.00E+17	2.925	663.342
4.00E+15	0.6	0.4	6.00E+17	2.981	793.437
4.00E+15	0.6	0.4	7.00E+17	3.110	922.080
4.00E+15	0.6	0.4	9.00E+17	3.391	1168.330
4.00E+15	0.6	0.4	1.00E+18	3.499	1277.210
6.00E+15	0.2	0.15	3.00E+17	0.372	58.805
6.00E+15	0.2	0.15	4.00E+17	0.384	68.694
6.00E+15	0.2	0.15	5.00E+17	0.392	79.032
6.00E+15	0.2	0.15	6.00E+17	0.397	89.682
6.00E+15	0.2	0.15	7.00E+17	0.405	100.754
6.00E+15	0.2	0.15	8.00E+17	0.407	112.329
6.00E+15	0.2	0.15	9.00E+17	0.410	124.823
6.00E+15	0.2	0.15	1.00E+18	0.412	138.144
6.00E+15	0.2	0.2	3.00E+17	0.397	68.718
6.00E+15	0.2	0.2	4.00E+17	0.409	82.567
6.00E+15	0.2	0.2	5.00E+17	0.419	97.160
6.00E+15	0.2	0.2	6.00E+17	0.426	112.689

6.00E+15	0.2	0.2	7.00E+17	0.428	128.421
6.00E+15	0.2	0.2	8.00E+17	0.432	142.267
6.00E+15	0.2	0.2	9.00E+17	0.433	156.373
6.00E+15	0.2	0.2	1.00E+18	0.435	167.863
6.00E+15	0.2	0.25	3.00E+17	0.418	78.518
6.00E+15	0.2	0.25	4.00E+17	0.433	96.389
6.00E+15	0.2	0.25	5.00E+17	0.442	115.818
6.00E+15	0.2	0.25	6.00E+17	0.447	137.707
6.00E+15	0.2	0.25	7.00E+17	0.451	162.338
6.00E+15	0.2	0.25	8.00E+17	0.457	188.203
6.00E+15	0.2	0.25	9.00E+17	0.459	219.475
6.00E+15	0.2	0.25	1.00E+18	0.462	249.711
6.00E+15	0.2	0.3	3.00E+17	0.444	88.111
6.00E+15	0.2	0.3	4.00E+17	0.455	110.345
6.00E+15	0.2	0.3	5.00E+17	0.466	135.856
6.00E+15	0.2	0.3	6.00E+17	0.475	165.241
6.00E+15	0.2	0.3	7.00E+17	0.480	198.369
6.00E+15	0.2	0.3	8.00E+17	0.484	233.037
6.00E+15	0.2	0.3	9.00E+17	0.490	272.926
6.00E+15	0.2	0.3	1.00E+18	0.493	316.336
6.00E+15	0.2	0.35	3.00E+17	0.466	97.560
6.00E+15	0.2	0.35	4.00E+17	0.482	125.090
6.00E+15	0.2	0.35	5.00E+17	0.497	157.370
6.00E+15	0.2	0.35	6.00E+17	0.503	194.833
6.00E+15	0.2	0.35	7.00E+17	0.513	198.896
6.00E+15	0.2	0.35	8.00E+17	0.517	283.421
6.00E+15	0.2	0.35	9.00E+17	0.523	332.633
6.00E+15	0.2	0.35	1.00E+18	0.525	387.733
6.00E+15	0.2	0.4	3.00E+17	0.489	107.083
6.00E+15	0.2	0.4	4.00E+17	0.513	140.534
6.00E+15	0.2	0.4	5.00E+17	0.524	180.599
6.00E+15	0.2	0.4	6.00E+17	0.537	226.346
6.00E+15	0.2	0.4	7.00E+17	0.546	278.338
6.00E+15	0.2	0.4	8.00E+17	0.549	336.282
6.00E+15	0.2	0.4	9.00E+17	0.555	396.577
6.00E+15	0.2	0.4	1.00E+18	0.563	461.955
6.00E+15	0.25	0.15	3.00E+17	0.382	50.064
6.00E+15	0.25	0.15	4.00E+17	0.397	59.826
6.00E+15	0.25	0.15	5.00E+17	0.407	70.160
6.00E+15	0.25	0.15	6.00E+17	0.416	80.897
6.00E+15	0.25	0.15	7.00E+17	0.422	92.075
6.00E+15	0.25	0.15	8.00E+17	0.428	103.701
6.00E+15	0.25	0.15	9.00E+17	0.430	116.296
6.00E+15	0.25	0.15	1.00E+18	0.434	129.815
6.00E+15	0.25	0.2	3.00E+17	0.416	60.350
6.00E+15	0.25	0.2	4.00E+17	0.430	74.646
6.00E+15	0.25	0.2	5.00E+17	0.447	89.696
6.00E+15	0.25	0.2	6.00E+17	0.453	105.667
6.00E+15	0.25	0.2	7.00E+17	0.453	123.436
6.00E+15	0.25	0.2	8.00E+17	0.457	143.327
6.00E+15	0.25	0.2	9.00E+17	0.460	165.047
6.00E+15	0.25	0.2	1.00E+18	0.465	188.794
6.00E+15	0.25	0.25	3.00E+17	0.447	70.234
6.00E+15	0.25	0.25	4.00E+17	0.467	88.601
6.00E+15	0.25	0.25	5.00E+17	0.476	108.545
6.00E+15	0.25	0.25	6.00E+17	0.486	131.273
6.00E+15	0.25	0.25	7.00E+17	0.488	157.218
6.00E+15	0.25	0.25	8.00E+17	0.496	186.123

6.00E+15	0.25	0.25	9.00E+17	0.503	217.876
6.00E+15	0.25	0.25	1.00E+18	0.506	252.559
6.00E+15	0.25	0.3	3.00E+17	0.473	79.934
6.00E+15	0.25	0.3	4.00E+17	0.492	102.705
6.00E+15	0.25	0.3	5.00E+17	0.506	129.057
6.00E+15	0.25	0.3	6.00E+17	0.518	159.910
6.00E+15	0.25	0.3	7.00E+17	0.524	194.818
6.00E+15	0.25	0.3	8.00E+17	0.533	233.910
6.00E+15	0.25	0.3	9.00E+17	0.534	277.002
6.00E+15	0.25	0.3	1.00E+18	0.544	324.019
6.00E+15	0.25	0.35	3.00E+17	0.504	89.552
6.00E+15	0.25	0.35	4.00E+17	0.535	117.667
6.00E+15	0.25	0.35	5.00E+17	0.549	151.657
6.00E+15	0.25	0.35	6.00E+17	0.560	191.204
6.00E+15	0.25	0.35	7.00E+17	0.566	236.295
6.00E+15	0.25	0.35	8.00E+17	0.574	286.828
6.00E+15	0.25	0.35	9.00E+17	0.578	342.520
6.00E+15	0.25	0.35	1.00E+18	0.586	401.785
6.00E+15	0.25	0.4	3.00E+17	0.529	99.371
6.00E+15	0.25	0.4	4.00E+17	0.568	134.145
6.00E+15	0.25	0.4	5.00E+17	0.591	176.251
6.00E+15	0.25	0.4	6.00E+17	0.598	225.340
6.00E+15	0.25	0.4	7.00E+17	0.612	281.603
6.00E+15	0.25	0.4	8.00E+17	0.618	344.444
6.00E+15	0.25	0.4	9.00E+17	0.623	370.570
6.00E+15	0.25	0.4	1.00E+18	0.628	479.038
6.00E+15	0.3	0.15	3.00E+17	0.395	53.020
6.00E+15	0.3	0.15	4.00E+17	0.411	64.087
6.00E+15	0.3	0.15	5.00E+17	0.428	75.795
6.00E+15	0.3	0.15	6.00E+17	0.439	87.970
6.00E+15	0.3	0.15	7.00E+17	0.447	100.751
6.00E+15	0.3	0.15	8.00E+17	0.453	114.700
6.00E+15	0.3	0.15	9.00E+17	0.456	129.812
6.00E+15	0.3	0.15	1.00E+18	0.460	146.473
6.00E+15	0.3	0.2	3.00E+17	0.435	64.623
6.00E+15	0.3	0.2	4.00E+17	0.456	80.691
6.00E+15	0.3	0.2	5.00E+17	0.474	97.746
6.00E+15	0.3	0.2	6.00E+17	0.482	116.780
6.00E+15	0.3	0.2	7.00E+17	0.490	138.308
6.00E+15	0.3	0.2	8.00E+17	0.489	162.143
6.00E+15	0.3	0.2	9.00E+17	0.498	191.054
6.00E+15	0.3	0.2	1.00E+18	0.503	217.126
6.00E+15	0.3	0.25	3.00E+17	0.472	75.895
6.00E+15	0.3	0.25	4.00E+17	0.501	96.687
6.00E+15	0.3	0.25	5.00E+17	0.518	122.207
6.00E+15	0.3	0.25	6.00E+17	0.527	148.171
6.00E+15	0.3	0.25	7.00E+17	0.530	182.170
6.00E+15	0.3	0.25	8.00E+17	0.541	214.434
6.00E+15	0.3	0.25	9.00E+17	0.549	252.944
6.00E+15	0.3	0.25	1.00E+18	0.557	299.065
6.00E+15	0.3	0.3	3.00E+17	0.511	87.135
6.00E+15	0.3	0.3	4.00E+17	0.548	113.870
6.00E+15	0.3	0.3	5.00E+17	0.565	146.122
6.00E+15	0.3	0.3	6.00E+17	0.577	186.224
6.00E+15	0.3	0.3	7.00E+17	0.586	225.876
6.00E+15	0.3	0.3	8.00E+17	0.594	277.333
6.00E+15	0.3	0.3	9.00E+17	0.603	326.267
6.00E+15	0.3	0.3	1.00E+18	0.606	388.024

6.00E+15	0.3	0.35	3.00E+17	0.548	98.440
6.00E+15	0.3	0.35	4.00E+17	0.595	134.722
6.00E+15	0.3	0.35	5.00E+17	0.614	176.791
6.00E+15	0.3	0.35	6.00E+17	0.627	222.422
6.00E+15	0.3	0.35	7.00E+17	0.637	281.652
6.00E+15	0.3	0.35	8.00E+17	0.648	339.626
6.00E+15	0.3	0.35	9.00E+17	0.655	410.711
6.00E+15	0.3	0.35	1.00E+18	0.663	472.809
6.00E+15	0.3	0.4	3.00E+17	0.597	110.534
6.00E+15	0.3	0.4	4.00E+17	0.645	155.302
6.00E+15	0.3	0.4	5.00E+17	0.662	207.686
6.00E+15	0.3	0.4	6.00E+17	0.673	268.804
6.00E+15	0.3	0.4	7.00E+17	0.695	338.835
6.00E+15	0.3	0.4	8.00E+17	0.705	409.297
6.00E+15	0.3	0.4	9.00E+17	0.718	490.287
6.00E+15	0.3	0.4	1.00E+18	0.730	565.088
6.00E+15	0.35	0.15	3.00E+17	0.409	56.623
6.00E+15	0.35	0.15	4.00E+17	0.431	68.960
6.00E+15	0.35	0.15	5.00E+17	0.449	81.897
6.00E+15	0.35	0.15	6.00E+17	0.471	95.508
6.00E+15	0.35	0.15	7.00E+17	0.478	110.418
6.00E+15	0.35	0.15	8.00E+17	0.483	126.708
6.00E+15	0.35	0.15	9.00E+17	0.491	144.873
6.00E+15	0.35	0.15	1.00E+18	0.496	164.295
6.00E+15	0.35	0.2	3.00E+17	0.459	69.721
6.00E+15	0.35	0.2	4.00E+17	0.496	87.631
6.00E+15	0.35	0.2	5.00E+17	0.507	107.279
6.00E+15	0.35	0.2	6.00E+17	0.524	129.680
6.00E+15	0.35	0.2	7.00E+17	0.506	155.219
6.00E+15	0.35	0.2	8.00E+17	0.536	183.706
6.00E+15	0.35	0.2	9.00E+17	0.547	214.889
6.00E+15	0.35	0.2	1.00E+18	0.556	248.969
6.00E+15	0.35	0.25	3.00E+17	0.504	82.467
6.00E+15	0.35	0.25	4.00E+17	0.549	106.428
6.00E+15	0.35	0.25	5.00E+17	0.569	134.792
6.00E+15	0.35	0.25	6.00E+17	0.578	167.733
6.00E+15	0.35	0.25	7.00E+17	0.593	204.748
6.00E+15	0.35	0.25	8.00E+17	0.603	246.964
6.00E+15	0.35	0.25	9.00E+17	0.609	292.998
6.00E+15	0.35	0.25	1.00E+18	0.619	343.343
6.00E+15	0.35	0.3	3.00E+17	0.558	95.316
6.00E+15	0.35	0.3	4.00E+17	0.605	127.193
6.00E+15	0.35	0.3	5.00E+17	0.623	165.678
6.00E+15	0.35	0.3	6.00E+17	0.634	210.651
6.00E+15	0.35	0.3	7.00E+17	0.648	261.668
6.00E+15	0.35	0.3	8.00E+17	0.663	318.888
6.00E+15	0.35	0.3	9.00E+17	0.672	381.523
6.00E+15	0.35	0.3	1.00E+18	0.682	445.603
6.00E+15	0.35	0.35	3.00E+17	0.616	109.037
6.00E+15	0.35	0.35	4.00E+17	0.664	150.135
6.00E+15	0.35	0.35	5.00E+17	0.688	199.977
6.00E+15	0.35	0.35	6.00E+17	0.708	258.153
6.00E+15	0.35	0.35	7.00E+17	0.721	324.742
6.00E+15	0.35	0.35	8.00E+17	0.734	397.729
6.00E+15	0.35	0.35	9.00E+17	0.743	471.684
6.00E+15	0.35	0.35	1.00E+18	0.753	545.156
6.00E+15	0.35	0.4	3.00E+17	0.676	123.943
6.00E+15	0.35	0.4	4.00E+17	0.733	175.211

6.00E+15	0.35	0.4	5.00E+17	0.762	237.465
6.00E+15	0.35	0.4	6.00E+17	0.777	310.390
6.00E+15	0.35	0.4	7.00E+17	0.795	392.467
6.00E+15	0.35	0.4	8.00E+17	0.804	476.143
6.00E+15	0.35	0.4	9.00E+17	0.818	559.789
6.00E+15	0.35	0.4	1.00E+18	0.833	642.911
6.00E+15	0.4	0.15	3.00E+17	0.424	58.892
6.00E+15	0.4	0.15	4.00E+17	0.452	72.264
6.00E+15	0.4	0.15	5.00E+17	0.480	86.386
6.00E+15	0.4	0.15	6.00E+17	0.502	101.488
6.00E+15	0.4	0.15	7.00E+17	0.515	118.256
6.00E+15	0.4	0.15	8.00E+17	0.525	137.093
6.00E+15	0.4	0.15	9.00E+17	0.536	157.606
6.00E+15	0.4	0.15	1.00E+18	0.545	180.077
6.00E+15	0.4	0.2	3.00E+17	0.485	73.110
6.00E+15	0.4	0.2	4.00E+17	0.537	92.592
6.00E+15	0.4	0.2	5.00E+17	0.562	114.732
6.00E+15	0.4	0.2	6.00E+17	0.570	140.448
6.00E+15	0.4	0.2	7.00E+17	0.581	169.507
6.00E+15	0.4	0.2	8.00E+17	0.595	201.868
6.00E+15	0.4	0.2	9.00E+17	0.607	237.546
6.00E+15	0.4	0.2	1.00E+18	0.615	276.435
6.00E+15	0.4	0.25	3.00E+17	0.548	87.128
6.00E+15	0.4	0.25	4.00E+17	0.610	114.085
6.00E+15	0.4	0.25	5.00E+17	0.630	146.634
6.00E+15	0.4	0.25	6.00E+17	0.638	184.292
6.00E+15	0.4	0.25	7.00E+17	0.655	227.140
6.00E+15	0.4	0.25	8.00E+17	0.671	275.102
6.00E+15	0.4	0.25	9.00E+17	0.682	328.057
6.00E+15	0.4	0.25	1.00E+18	0.691	385.220
6.00E+15	0.4	0.3	3.00E+17	0.625	101.875
6.00E+15	0.4	0.3	4.00E+17	0.679	138.534
6.00E+15	0.4	0.3	5.00E+17	0.698	182.587
6.00E+15	0.4	0.3	6.00E+17	0.717	234.263
6.00E+15	0.4	0.3	7.00E+17	0.739	293.201
6.00E+15	0.4	0.3	8.00E+17	0.752	358.953
6.00E+15	0.4	0.3	9.00E+17	0.762	428.146
6.00E+15	0.4	0.3	1.00E+18	0.772	362.139
6.00E+15	0.4	0.35	3.00E+17	0.693	117.686
6.00E+15	0.4	0.35	4.00E+17	0.751	165.274
6.00E+15	0.4	0.35	5.00E+17	0.779	222.658
6.00E+15	0.4	0.35	6.00E+17	0.799	290.074
6.00E+15	0.4	0.35	7.00E+17	0.819	366.541
6.00E+15	0.4	0.35	8.00E+17	0.833	446.686
6.00E+15	0.4	0.35	9.00E+17	0.845	526.689
6.00E+15	0.4	0.35	1.00E+18	0.858	606.547
6.00E+15	0.4	0.4	3.00E+17	0.778	135.480
6.00E+15	0.4	0.4	4.00E+17	0.832	194.619
6.00E+15	0.4	0.4	5.00E+17	0.855	266.828
6.00E+15	0.4	0.4	6.00E+17	0.886	351.471
6.00E+15	0.4	0.4	7.00E+17	0.911	442.125
6.00E+15	0.4	0.4	8.00E+17	0.926	532.910
6.00E+15	0.4	0.4	9.00E+17	0.941	623.437
6.00E+15	0.4	0.4	1.00E+18	0.951	713.368
6.00E+15	0.45	0.15	3.00E+17	0.444	61.233
6.00E+15	0.45	0.15	4.00E+17	0.474	75.568
6.00E+15	0.45	0.15	5.00E+17	0.515	90.851
6.00E+15	0.45	0.15	6.00E+17	0.544	107.779

6.00E+15	0.45	0.15	7.00E+17	0.562	126.750
6.00E+15	0.45	0.15	8.00E+17	0.572	147.991
6.00E+15	0.45	0.15	9.00E+17	0.587	171.275
6.00E+15	0.45	0.15	1.00E+18	0.599	196.462
6.00E+15	0.45	0.2	3.00E+17	0.519	76.533
6.00E+15	0.45	0.2	4.00E+17	0.582	98.030
6.00E+15	0.45	0.2	5.00E+17	0.612	122.929
6.00E+15	0.45	0.2	6.00E+17	0.633	152.000
6.00E+15	0.45	0.2	7.00E+17	0.632	184.911
6.00E+15	0.45	0.2	8.00E+17	0.659	221.622
6.00E+15	0.45	0.2	9.00E+17	0.671	262.074
6.00E+15	0.45	0.2	1.00E+18	0.675	306.377
6.00E+15	0.45	0.25	3.00E+17	0.601	91.895
6.00E+15	0.45	0.25	4.00E+17	0.673	122.377
6.00E+15	0.45	0.25	5.00E+17	0.702	159.156
6.00E+15	0.45	0.25	6.00E+17	0.721	202.056
6.00E+15	0.45	0.25	7.00E+17	0.736	250.762
6.00E+15	0.45	0.25	8.00E+17	0.719	305.302
6.00E+15	0.45	0.25	9.00E+17	0.767	365.119
6.00E+15	0.45	0.25	1.00E+18	0.777	426.768
6.00E+15	0.45	0.3	3.00E+17	0.698	108.672
6.00E+15	0.45	0.3	4.00E+17	0.745	150.218
6.00E+15	0.45	0.3	5.00E+17	0.780	200.549
6.00E+15	0.45	0.3	6.00E+17	0.799	259.190
6.00E+15	0.45	0.3	7.00E+17	0.834	326.490
6.00E+15	0.45	0.3	8.00E+17	0.855	399.820
6.00E+15	0.45	0.3	9.00E+17	0.865	474.147
6.00E+15	0.45	0.3	1.00E+18	0.873	548.417
6.00E+15	0.45	0.35	3.00E+17	0.785	127.080
6.00E+15	0.45	0.35	4.00E+17	0.852	180.980
6.00E+15	0.45	0.35	5.00E+17	0.891	246.549
6.00E+15	0.45	0.35	6.00E+17	0.917	323.513
6.00E+15	0.45	0.35	7.00E+17	0.938	408.437
6.00E+15	0.45	0.35	8.00E+17	0.958	494.458
6.00E+15	0.45	0.35	9.00E+17	0.978	580.317
6.00E+15	0.45	0.35	1.00E+18	0.997	666.012
6.00E+15	0.45	0.4	3.00E+17	0.878	147.340
6.00E+15	0.45	0.4	4.00E+17	0.950	214.846
6.00E+15	0.45	0.4	5.00E+17	1.009	297.390
6.00E+15	0.45	0.4	6.00E+17	1.029	392.722
6.00E+15	0.45	0.4	7.00E+17	1.067	490.154
6.00E+15	0.45	0.4	8.00E+17	1.083	541.134
6.00E+15	0.45	0.4	9.00E+17	1.114	684.980
6.00E+15	0.45	0.4	1.00E+18	1.134	779.922
6.00E+15	0.5	0.15	3.00E+17	0.464	117.137
6.00E+15	0.5	0.15	4.00E+17	0.500	141.074
6.00E+15	0.5	0.15	5.00E+17	0.553	167.878
6.00E+15	0.5	0.15	6.00E+17	0.590	160.249
6.00E+15	0.5	0.15	7.00E+17	0.615	171.104
6.00E+15	0.5	0.15	8.00E+17	0.631	182.882
6.00E+15	0.5	0.15	9.00E+17	0.648	194.331
6.00E+15	0.5	0.15	1.00E+18	0.662	206.237
6.00E+15	0.5	0.2	3.00E+17	0.556	144.192
6.00E+15	0.5	0.2	4.00E+17	0.634	182.176
6.00E+15	0.5	0.2	5.00E+17	0.675	227.145
6.00E+15	0.5	0.2	6.00E+17	0.690	275.692
6.00E+15	0.5	0.2	7.00E+17	0.716	329.948
6.00E+15	0.5	0.2	8.00E+17	0.739	388.514

6.00E+15	0.5	0.2	9.00E+17	0.745	446.219
6.00E+15	0.5	0.2	1.00E+18	0.772	508.090
6.00E+15	0.5	0.25	3.00E+17	0.659	175.300
6.00E+15	0.5	0.25	4.00E+17	0.742	229.428
6.00E+15	0.5	0.25	5.00E+17	0.781	291.796
6.00E+15	0.5	0.25	6.00E+17	0.808	363.243
6.00E+15	0.5	0.25	7.00E+17	0.834	438.355
6.00E+15	0.5	0.25	8.00E+17	0.855	513.573
6.00E+15	0.5	0.25	9.00E+17	0.870	588.990
6.00E+15	0.5	0.25	1.00E+18	0.885	663.688
6.00E+15	0.5	0.3	3.00E+17	0.787	191.800
6.00E+15	0.5	0.3	4.00E+17	0.852	279.789
6.00E+15	0.5	0.3	5.00E+17	0.901	364.578
6.00E+15	0.5	0.3	6.00E+17	0.922	302.898
6.00E+15	0.5	0.3	7.00E+17	0.941	544.722
6.00E+15	0.5	0.3	8.00E+17	0.989	634.910
6.00E+15	0.5	0.3	9.00E+17	0.998	724.334
6.00E+15	0.5	0.3	1.00E+18	1.010	812.086
6.00E+15	0.5	0.35	3.00E+17	0.892	241.333
6.00E+15	0.5	0.35	4.00E+17	0.967	315.956
6.00E+15	0.5	0.35	5.00E+17	1.008	438.102
6.00E+15	0.5	0.35	6.00E+17	1.070	543.051
6.00E+15	0.5	0.35	7.00E+17	1.099	647.890
6.00E+15	0.5	0.35	8.00E+17	1.121	751.542
6.00E+15	0.5	0.35	9.00E+17	1.149	853.653
6.00E+15	0.5	0.35	1.00E+18	1.169	952.092
6.00E+15	0.5	0.4	3.00E+17	1.003	278.119
6.00E+15	0.5	0.4	4.00E+17	1.104	391.782
6.00E+15	0.5	0.4	5.00E+17	1.173	445.845
6.00E+15	0.5	0.4	6.00E+17	1.222	627.720
6.00E+15	0.5	0.4	7.00E+17	1.259	747.886
6.00E+15	0.5	0.4	8.00E+17	1.295	864.355
6.00E+15	0.5	0.4	9.00E+17	1.334	977.030
6.00E+15	0.5	0.4	1.00E+18	1.362	1083.470
6.00E+15	0.6	0.15	3.00E+17	0.505	122.680
6.00E+15	0.6	0.15	4.00E+17	0.551	148.468
6.00E+15	0.6	0.15	5.00E+17	0.632	179.990
6.00E+15	0.6	0.15	6.00E+17	0.694	211.731
6.00E+15	0.6	0.15	7.00E+17	0.745	250.478
6.00E+15	0.6	0.15	8.00E+17	0.780	291.152
6.00E+15	0.6	0.15	9.00E+17	0.799	334.481
6.00E+15	0.6	0.15	1.00E+18	0.819	380.767
6.00E+15	0.6	0.2	3.00E+17	0.640	152.031
6.00E+15	0.6	0.2	4.00E+17	0.758	194.619
6.00E+15	0.6	0.2	5.00E+17	0.838	243.637
6.00E+15	0.6	0.2	6.00E+17	0.878	301.005
6.00E+15	0.6	0.2	7.00E+17	0.911	362.417
6.00E+15	0.6	0.2	8.00E+17	0.943	426.864
6.00E+15	0.6	0.2	9.00E+17	0.961	489.136
6.00E+15	0.6	0.2	1.00E+18	0.987	556.004
6.00E+15	0.6	0.25	3.00E+17	0.824	185.774
6.00E+15	0.6	0.25	4.00E+17	0.951	249.081
6.00E+15	0.6	0.25	5.00E+17	1.018	320.780
6.00E+15	0.6	0.25	6.00E+17	1.062	401.326
6.00E+15	0.6	0.25	7.00E+17	1.096	480.139
6.00E+15	0.6	0.25	8.00E+17	1.142	561.680
6.00E+15	0.6	0.25	9.00E+17	1.167	645.344
6.00E+15	0.6	0.25	1.00E+18	1.195	726.064

6.00E+15	0.6	0.3	3.00E+17	1.011	224.929
6.00E+15	0.6	0.3	4.00E+17	1.134	242.011
6.00E+15	0.6	0.3	5.00E+17	1.226	403.685
6.00E+15	0.6	0.3	6.00E+17	1.265	499.288
6.00E+15	0.6	0.3	7.00E+17	1.321	599.551
6.00E+15	0.6	0.3	1.00E+18	1.425	888.884
6.00E+15	0.6	0.35	3.00E+17	1.201	262.676
6.00E+15	0.6	0.35	4.00E+17	1.336	371.326
6.00E+15	0.6	0.35	5.00E+17	1.487	454.103
6.00E+15	0.6	0.35	6.00E+17	1.517	599.685
6.00E+15	0.6	0.35	7.00E+17	1.587	713.622
6.00E+15	0.6	0.35	8.00E+17	1.626	826.565
6.00E+15	0.6	0.35	9.00E+17	1.674	936.237
6.00E+15	0.6	0.4	3.00E+17	1.409	308.111
6.00E+15	0.6	0.4	4.00E+17	1.579	433.923
6.00E+15	0.6	0.4	5.00E+17	1.821	566.042
6.00E+15	0.6	0.4	6.00E+17	1.824	696.195
6.00E+15	0.6	0.4	7.00E+17	1.882	824.928
6.00E+15	0.6	0.4	8.00E+17	1.952	950.723
6.00E+15	0.6	0.4	9.00E+17	2.026	1071.600
6.00E+15	0.6	0.4	1.00E+18	2.082	1185.040
8.00E+15	0.2	0.15	3.00E+17	0.282	52.709
8.00E+15	0.2	0.15	4.00E+17	0.287	61.555
8.00E+15	0.2	0.15	5.00E+17	0.295	70.725
8.00E+15	0.2	0.15	6.00E+17	0.298	80.331
8.00E+15	0.2	0.15	7.00E+17	0.302	90.261
8.00E+15	0.2	0.15	8.00E+17	0.305	100.544
8.00E+15	0.2	0.15	9.00E+17	0.306	111.041
8.00E+15	0.2	0.15	1.00E+18	0.307	122.041
8.00E+15	0.2	0.2	3.00E+17	0.299	61.564
8.00E+15	0.2	0.2	4.00E+17	0.305	73.965
8.00E+15	0.2	0.2	5.00E+17	0.312	87.038
8.00E+15	0.2	0.2	6.00E+17	0.316	100.773
8.00E+15	0.2	0.2	7.00E+17	0.318	114.962
8.00E+15	0.2	0.2	8.00E+17	0.320	129.878
8.00E+15	0.2	0.2	9.00E+17	0.323	132.685
8.00E+15	0.2	0.2	1.00E+18	0.325	141.116
8.00E+15	0.2	0.25	3.00E+17	0.313	70.317
8.00E+15	0.2	0.25	4.00E+17	0.321	86.345
8.00E+15	0.2	0.25	5.00E+17	0.328	103.373
8.00E+15	0.2	0.25	6.00E+17	0.332	121.302
8.00E+15	0.2	0.25	7.00E+17	0.333	139.837
8.00E+15	0.2	0.25	8.00E+17	0.336	159.107
8.00E+15	0.2	0.25	9.00E+17	0.338	182.518
8.00E+15	0.2	0.25	1.00E+18	0.339	205.587
8.00E+15	0.2	0.3	3.00E+17	0.329	78.891
8.00E+15	0.2	0.3	4.00E+17	0.336	98.639
8.00E+15	0.2	0.3	5.00E+17	0.342	119.460
8.00E+15	0.2	0.3	6.00E+17	0.345	141.622
8.00E+15	0.2	0.3	7.00E+17	0.349	166.334
8.00E+15	0.2	0.3	8.00E+17	0.351	192.580
8.00E+15	0.2	0.3	9.00E+17	0.354	224.150
8.00E+15	0.2	0.3	1.00E+18	0.356	257.069
8.00E+15	0.2	0.35	3.00E+17	0.341	87.325
8.00E+15	0.2	0.35	4.00E+17	0.349	110.543
8.00E+15	0.2	0.35	5.00E+17	0.356	135.352
8.00E+15	0.2	0.35	6.00E+17	0.360	163.241
8.00E+15	0.2	0.35	7.00E+17	0.364	193.605

8.00E+15	0.2	0.35	8.00E+17	0.367	228.770
8.00E+15	0.2	0.35	9.00E+17	0.371	201.932
8.00E+15	0.2	0.35	1.00E+18	0.373	311.472
8.00E+15	0.2	0.4	3.00E+17	0.356	95.631
8.00E+15	0.2	0.4	4.00E+17	0.364	122.422
8.00E+15	0.2	0.4	5.00E+17	0.373	152.325
8.00E+15	0.2	0.4	6.00E+17	0.378	185.360
8.00E+15	0.2	0.4	7.00E+17	0.386	225.803
8.00E+15	0.2	0.4	8.00E+17	0.388	269.615
8.00E+15	0.2	0.4	9.00E+17	0.393	317.970
8.00E+15	0.2	0.4	1.00E+18	0.394	368.828
8.00E+15	0.25	0.15	3.00E+17	0.289	44.933
8.00E+15	0.25	0.15	4.00E+17	0.298	53.712
8.00E+15	0.25	0.15	5.00E+17	0.306	62.944
8.00E+15	0.25	0.15	6.00E+17	0.312	72.515
8.00E+15	0.25	0.15	7.00E+17	0.316	82.538
8.00E+15	0.25	0.15	8.00E+17	0.318	92.951
8.00E+15	0.25	0.15	9.00E+17	0.323	103.657
8.00E+15	0.25	0.15	1.00E+18	0.323	114.703
8.00E+15	0.25	0.2	3.00E+17	0.310	54.199
8.00E+15	0.25	0.2	4.00E+17	0.321	66.918
8.00E+15	0.25	0.2	5.00E+17	0.328	80.417
8.00E+15	0.25	0.2	6.00E+17	0.332	94.659
8.00E+15	0.25	0.2	7.00E+17	0.334	109.460
8.00E+15	0.25	0.2	8.00E+17	0.337	124.840
8.00E+15	0.25	0.2	9.00E+17	0.339	141.180
8.00E+15	0.25	0.2	1.00E+18	0.343	158.860
8.00E+15	0.25	0.25	3.00E+17	0.331	63.019
8.00E+15	0.25	0.25	4.00E+17	0.338	79.391
8.00E+15	0.25	0.25	5.00E+17	0.347	96.958
8.00E+15	0.25	0.25	6.00E+17	0.353	115.433
8.00E+15	0.25	0.25	7.00E+17	0.358	134.972
8.00E+15	0.25	0.25	8.00E+17	0.359	156.443
8.00E+15	0.25	0.25	9.00E+17	0.361	180.406
8.00E+15	0.25	0.25	1.00E+18	0.365	206.475
8.00E+15	0.25	0.3	3.00E+17	0.348	71.636
8.00E+15	0.25	0.3	4.00E+17	0.358	91.872
8.00E+15	0.25	0.3	5.00E+17	0.366	113.437
8.00E+15	0.25	0.3	6.00E+17	0.373	136.569
8.00E+15	0.25	0.3	7.00E+17	0.374	162.605
8.00E+15	0.25	0.3	8.00E+17	0.384	192.172
8.00E+15	0.25	0.3	9.00E+17	0.383	224.597
8.00E+15	0.25	0.3	1.00E+18	0.386	260.172
8.00E+15	0.25	0.35	3.00E+17	0.362	80.191
8.00E+15	0.25	0.35	4.00E+17	0.381	104.125
8.00E+15	0.25	0.35	5.00E+17	0.387	129.991
8.00E+15	0.25	0.35	6.00E+17	0.396	159.375
8.00E+15	0.25	0.35	7.00E+17	0.402	193.426
8.00E+15	0.25	0.35	8.00E+17	0.408	231.512
8.00E+15	0.25	0.35	9.00E+17	0.409	273.548
8.00E+15	0.25	0.35	1.00E+18	0.414	319.766
8.00E+15	0.25	0.4	3.00E+17	0.383	88.784
8.00E+15	0.25	0.4	4.00E+17	0.401	116.520
8.00E+15	0.25	0.4	5.00E+17	0.415	147.796
8.00E+15	0.25	0.4	6.00E+17	0.419	184.498
8.00E+15	0.25	0.4	7.00E+17	0.429	226.992
8.00E+15	0.25	0.4	8.00E+17	0.431	274.481
8.00E+15	0.25	0.4	9.00E+17	0.440	327.302

8.00E+15	0.25	0.4	1.00E+18	0.438	385.103
8.00E+15	0.3	0.15	3.00E+17	0.299	47.527
8.00E+15	0.3	0.15	4.00E+17	0.311	57.429
8.00E+15	0.3	0.15	5.00E+17	0.320	67.814
8.00E+15	0.3	0.15	6.00E+17	0.327	78.708
8.00E+15	0.3	0.15	7.00E+17	0.333	90.194
8.00E+15	0.3	0.15	8.00E+17	0.337	101.978
8.00E+15	0.3	0.15	9.00E+17	0.341	114.170
8.00E+15	0.3	0.15	1.00E+18	0.343	126.779
8.00E+15	0.3	0.2	3.00E+17	0.326	57.954
8.00E+15	0.3	0.2	4.00E+17	0.338	72.242
8.00E+15	0.3	0.2	5.00E+17	0.348	88.853
8.00E+15	0.3	0.2	6.00E+17	0.354	103.582
8.00E+15	0.3	0.2	7.00E+17	0.359	120.493
8.00E+15	0.3	0.2	8.00E+17	0.363	138.433
8.00E+15	0.3	0.2	9.00E+17	0.364	158.038
8.00E+15	0.3	0.2	1.00E+18	0.366	179.662
8.00E+15	0.3	0.25	3.00E+17	0.351	67.960
8.00E+15	0.3	0.25	4.00E+17	0.367	86.657
8.00E+15	0.3	0.25	5.00E+17	0.374	106.437
8.00E+15	0.3	0.25	6.00E+17	0.380	127.551
8.00E+15	0.3	0.25	7.00E+17	0.387	150.984
8.00E+15	0.3	0.25	8.00E+17	0.395	177.247
8.00E+15	0.3	0.25	9.00E+17	0.393	209.012
8.00E+15	0.3	0.25	1.00E+18	0.398	238.242
8.00E+15	0.3	0.3	3.00E+17	0.371	77.966
8.00E+15	0.3	0.3	4.00E+17	0.394	100.989
8.00E+15	0.3	0.3	5.00E+17	0.403	125.667
8.00E+15	0.3	0.3	6.00E+17	0.411	155.632
8.00E+15	0.3	0.3	7.00E+17	0.419	185.720
8.00E+15	0.3	0.3	8.00E+17	0.424	224.608
8.00E+15	0.3	0.3	9.00E+17	0.426	261.357
8.00E+15	0.3	0.3	1.00E+18	0.433	308.667
8.00E+15	0.3	0.35	3.00E+17	0.396	87.958
8.00E+15	0.3	0.35	4.00E+17	0.422	115.330
8.00E+15	0.3	0.35	5.00E+17	0.433	146.156
8.00E+15	0.3	0.35	6.00E+17	0.440	182.228
8.00E+15	0.3	0.35	7.00E+17	0.456	227.142
8.00E+15	0.3	0.35	8.00E+17	0.456	270.805
8.00E+15	0.3	0.35	9.00E+17	0.464	326.912
8.00E+15	0.3	0.35	1.00E+18	0.467	379.663
8.00E+15	0.3	0.4	3.00E+17	0.419	99.261
8.00E+15	0.3	0.4	4.00E+17	0.447	130.125
8.00E+15	0.3	0.4	5.00E+17	0.456	170.886
8.00E+15	0.3	0.4	6.00E+17	0.475	213.962
8.00E+15	0.3	0.4	7.00E+17	0.490	266.166
8.00E+15	0.3	0.4	8.00E+17	0.490	329.108
8.00E+15	0.3	0.4	9.00E+17	0.496	390.203
8.00E+15	0.3	0.4	1.00E+18	0.505	467.020
8.00E+15	0.35	0.15	3.00E+17	0.308	50.744
8.00E+15	0.35	0.15	4.00E+17	0.325	61.706
8.00E+15	0.35	0.15	5.00E+17	0.336	73.204
8.00E+15	0.35	0.15	6.00E+17	0.347	85.452
8.00E+15	0.35	0.15	7.00E+17	0.358	98.118
8.00E+15	0.35	0.15	8.00E+17	0.359	111.285
8.00E+15	0.35	0.15	9.00E+17	0.362	124.962
8.00E+15	0.35	0.15	1.00E+18	0.365	139.710
8.00E+15	0.35	0.2	3.00E+17	0.342	62.407

8.00E+15	0.35	0.2	4.00E+17	0.363	78.351
8.00E+15	0.35	0.2	5.00E+17	0.373	95.504
8.00E+15	0.35	0.2	6.00E+17	0.381	113.475
8.00E+15	0.35	0.2	7.00E+17	0.396	132.677
8.00E+15	0.35	0.2	8.00E+17	0.396	153.897
8.00E+15	0.35	0.2	9.00E+17	0.398	177.423
8.00E+15	0.35	0.2	1.00E+18	0.402	203.097
8.00E+15	0.35	0.25	3.00E+17	0.375	73.725
8.00E+15	0.35	0.25	4.00E+17	0.402	94.711
8.00E+15	0.35	0.25	5.00E+17	0.412	117.056
8.00E+15	0.35	0.25	6.00E+17	0.416	141.746
8.00E+15	0.35	0.25	7.00E+17	0.435	169.698
8.00E+15	0.35	0.25	8.00E+17	0.434	201.246
8.00E+15	0.35	0.25	9.00E+17	0.433	222.394
8.00E+15	0.35	0.25	1.00E+18	0.445	244.455
8.00E+15	0.35	0.3	3.00E+17	0.411	85.226
8.00E+15	0.35	0.3	4.00E+17	0.438	111.076
8.00E+15	0.35	0.3	5.00E+17	0.450	139.934
8.00E+15	0.35	0.3	6.00E+17	0.462	173.488
8.00E+15	0.35	0.3	7.00E+17	0.472	211.903
8.00E+15	0.35	0.3	8.00E+17	0.469	255.171
8.00E+15	0.35	0.3	9.00E+17	0.482	303.171
8.00E+15	0.35	0.3	1.00E+18	0.487	355.659
8.00E+15	0.35	0.35	3.00E+17	0.440	96.528
8.00E+15	0.35	0.35	4.00E+17	0.473	127.884
8.00E+15	0.35	0.35	5.00E+17	0.492	164.999
8.00E+15	0.35	0.35	6.00E+17	0.500	208.802
8.00E+15	0.35	0.35	7.00E+17	0.511	259.057
8.00E+15	0.35	0.35	8.00E+17	0.520	315.698
8.00E+15	0.35	0.35	9.00E+17	0.525	378.397
8.00E+15	0.35	0.35	1.00E+18	0.532	447.102
8.00E+15	0.35	0.4	3.00E+17	0.469	108.038
8.00E+15	0.35	0.4	4.00E+17	0.518	146.145
8.00E+15	0.35	0.4	5.00E+17	0.531	192.723
8.00E+15	0.35	0.4	6.00E+17	0.539	247.702
8.00E+15	0.35	0.4	7.00E+17	0.552	311.024
8.00E+15	0.35	0.4	8.00E+17	0.563	382.243
8.00E+15	0.35	0.4	9.00E+17	0.575	347.148
8.00E+15	0.35	0.4	1.00E+18	0.585	543.811
8.00E+15	0.4	0.15	3.00E+17	0.321	52.702
8.00E+15	0.4	0.15	4.00E+17	0.341	64.537
8.00E+15	0.4	0.15	5.00E+17	0.357	77.064
8.00E+15	0.4	0.15	6.00E+17	0.376	90.402
8.00E+15	0.4	0.15	7.00E+17	0.386	104.190
8.00E+15	0.4	0.15	8.00E+17	0.392	118.619
8.00E+15	0.4	0.15	9.00E+17	0.394	134.064
8.00E+15	0.4	0.15	1.00E+18	0.398	150.756
8.00E+15	0.4	0.2	3.00E+17	0.365	65.303
8.00E+15	0.4	0.2	4.00E+17	0.388	82.744
8.00E+15	0.4	0.2	5.00E+17	0.410	101.292
8.00E+15	0.4	0.2	6.00E+17	0.419	120.941
8.00E+15	0.4	0.2	7.00E+17	0.434	142.796
8.00E+15	0.4	0.2	8.00E+17	0.441	166.979
8.00E+15	0.4	0.2	9.00E+17	0.438	193.985
8.00E+15	0.4	0.2	1.00E+18	0.448	223.144
8.00E+15	0.4	0.25	3.00E+17	0.404	77.812
8.00E+15	0.4	0.25	4.00E+17	0.442	100.628
8.00E+15	0.4	0.25	5.00E+17	0.457	125.328

8.00E+15	0.4	0.25	6.00E+17	0.469	153.452
8.00E+15	0.4	0.25	7.00E+17	0.481	185.933
8.00E+15	0.4	0.25	8.00E+17	0.488	222.128
8.00E+15	0.4	0.25	9.00E+17	0.491	262.109
8.00E+15	0.4	0.25	1.00E+18	0.498	305.571
8.00E+15	0.4	0.3	3.00E+17	0.446	90.523
8.00E+15	0.4	0.3	4.00E+17	0.490	118.934
8.00E+15	0.4	0.3	5.00E+17	0.509	151.909
8.00E+15	0.4	0.3	6.00E+17	0.518	190.817
8.00E+15	0.4	0.3	7.00E+17	0.533	235.309
8.00E+15	0.4	0.3	8.00E+17	0.537	285.147
8.00E+15	0.4	0.3	9.00E+17	0.550	340.484
8.00E+15	0.4	0.3	1.00E+18	0.555	401.398
8.00E+15	0.4	0.35	3.00E+17	0.489	103.082
8.00E+15	0.4	0.35	4.00E+17	0.538	138.514
8.00E+15	0.4	0.35	5.00E+17	0.558	181.763
8.00E+15	0.4	0.35	6.00E+17	0.576	232.442
8.00E+15	0.4	0.35	7.00E+17	0.583	290.512
8.00E+15	0.4	0.35	8.00E+17	0.596	355.979
8.00E+15	0.4	0.35	9.00E+17	0.601	429.020
8.00E+15	0.4	0.35	1.00E+18	0.614	507.481
8.00E+15	0.4	0.4	3.00E+17	0.538	116.003
8.00E+15	0.4	0.4	4.00E+17	0.593	160.210
8.00E+15	0.4	0.4	5.00E+17	0.613	214.471
8.00E+15	0.4	0.4	6.00E+17	0.629	278.412
8.00E+15	0.4	0.4	7.00E+17	0.642	351.899
8.00E+15	0.4	0.4	8.00E+17	0.650	435.047
8.00E+15	0.4	0.4	9.00E+17	0.668	524.555
8.00E+15	0.4	0.4	1.00E+18	0.676	612.866
8.00E+15	0.45	0.15	3.00E+17	0.337	54.678
8.00E+15	0.45	0.15	4.00E+17	0.359	67.388
8.00E+15	0.45	0.15	5.00E+17	0.381	80.998
8.00E+15	0.45	0.15	6.00E+17	0.408	95.285
8.00E+15	0.45	0.15	7.00E+17	0.419	110.285
8.00E+15	0.45	0.15	8.00E+17	0.431	126.181
8.00E+15	0.45	0.15	9.00E+17	0.434	143.615
8.00E+15	0.45	0.15	1.00E+18	0.436	162.509
8.00E+15	0.45	0.2	3.00E+17	0.391	68.264
8.00E+15	0.45	0.2	4.00E+17	0.429	87.125
8.00E+15	0.45	0.2	5.00E+17	0.453	107.311
8.00E+15	0.45	0.2	6.00E+17	0.466	129.121
8.00E+15	0.45	0.2	7.00E+17	0.478	153.674
8.00E+15	0.45	0.2	8.00E+17	0.488	181.498
8.00E+15	0.45	0.2	9.00E+17	0.494	211.903
8.00E+15	0.45	0.2	1.00E+18	0.500	245.453
8.00E+15	0.45	0.25	3.00E+17	0.448	82.025
8.00E+15	0.45	0.25	4.00E+17	0.487	106.714
8.00E+15	0.45	0.25	5.00E+17	0.510	134.235
8.00E+15	0.45	0.25	6.00E+17	0.528	165.830
8.00E+15	0.45	0.25	7.00E+17	0.538	203.050
8.00E+15	0.45	0.25	8.00E+17	0.550	244.276
8.00E+15	0.45	0.25	9.00E+17	0.559	289.904
8.00E+15	0.45	0.25	1.00E+18	0.567	339.956
8.00E+15	0.45	0.3	3.00E+17	0.505	95.804
8.00E+15	0.45	0.3	4.00E+17	0.559	127.310
8.00E+15	0.45	0.3	5.00E+17	0.560	164.872
8.00E+15	0.45	0.3	6.00E+17	0.580	209.094
8.00E+15	0.45	0.3	7.00E+17	0.612	259.932

8.00E+15	0.45	0.3	8.00E+17	0.616	317.059
8.00E+15	0.45	0.3	9.00E+17	0.626	380.372
8.00E+15	0.45	0.3	1.00E+18	0.638	449.792
8.00E+15	0.45	0.35	3.00E+17	0.566	109.655
8.00E+15	0.45	0.35	4.00E+17	0.618	149.769
8.00E+15	0.45	0.35	5.00E+17	0.642	199.054
8.00E+15	0.45	0.35	6.00E+17	0.650	257.149
8.00E+15	0.45	0.35	7.00E+17	0.680	323.706
8.00E+15	0.45	0.35	8.00E+17	0.689	398.774
8.00E+15	0.45	0.35	9.00E+17	0.707	481.546
8.00E+15	0.45	0.35	1.00E+18	0.720	567.151
8.00E+15	0.45	0.4	3.00E+17	0.628	124.522
8.00E+15	0.45	0.4	4.00E+17	0.689	175.116
8.00E+15	0.45	0.4	5.00E+17	0.719	236.926
8.00E+15	0.45	0.4	6.00E+17	0.744	304.146
8.00E+15	0.45	0.4	7.00E+17	0.758	394.645
8.00E+15	0.45	0.4	8.00E+17	0.782	489.171
8.00E+15	0.45	0.4	9.00E+17	0.793	586.342
8.00E+15	0.45	0.4	1.00E+18	0.806	681.180
8.00E+15	0.5	0.15	3.00E+17	0.356	102.762
8.00E+15	0.5	0.15	4.00E+17	0.381	122.724
8.00E+15	0.5	0.15	5.00E+17	0.413	142.131
8.00E+15	0.5	0.15	6.00E+17	0.442	164.440
8.00E+15	0.5	0.15	7.00E+17	0.463	190.644
8.00E+15	0.5	0.15	8.00E+17	0.466	146.391
8.00E+15	0.5	0.15	9.00E+17	0.484	156.446
8.00E+15	0.5	0.15	1.00E+18	0.487	166.506
8.00E+15	0.5	0.2	3.00E+17	0.421	125.283
8.00E+15	0.5	0.2	4.00E+17	0.467	153.861
8.00E+15	0.5	0.2	5.00E+17	0.482	183.169
8.00E+15	0.5	0.2	6.00E+17	0.522	223.741
8.00E+15	0.5	0.2	7.00E+17	0.534	264.965
8.00E+15	0.5	0.2	8.00E+17	0.547	310.120
8.00E+15	0.5	0.2	9.00E+17	0.545	359.076
8.00E+15	0.5	0.2	1.00E+18	0.562	412.303
8.00E+15	0.5	0.25	3.00E+17	0.496	146.288
8.00E+15	0.5	0.25	4.00E+17	0.552	188.740
8.00E+15	0.5	0.25	5.00E+17	0.579	236.408
8.00E+15	0.5	0.25	6.00E+17	0.602	279.091
8.00E+15	0.5	0.25	7.00E+17	0.616	318.669
8.00E+15	0.5	0.25	8.00E+17	0.632	417.518
8.00E+15	0.5	0.25	9.00E+17	0.642	487.952
8.00E+15	0.5	0.25	1.00E+18	0.646	564.857
8.00E+15	0.5	0.3	3.00E+17	0.571	171.562
8.00E+15	0.5	0.3	4.00E+17	0.633	183.331
8.00E+15	0.5	0.3	5.00E+17	0.660	291.124
8.00E+15	0.5	0.3	6.00E+17	0.672	364.676
8.00E+15	0.5	0.3	7.00E+17	0.708	447.522
8.00E+15	0.5	0.3	1.00E+18	0.746	713.805
8.00E+15	0.5	0.35	3.00E+17	0.637	172.392
8.00E+15	0.5	0.35	4.00E+17	0.713	267.587
8.00E+15	0.5	0.35	5.00E+17	0.742	284.295
8.00E+15	0.5	0.35	6.00E+17	0.781	446.094
8.00E+15	0.5	0.35	7.00E+17	0.780	547.624
8.00E+15	0.5	0.35	8.00E+17	0.822	653.503
8.00E+15	0.5	0.35	9.00E+17	0.837	755.605
8.00E+15	0.5	0.35	1.00E+18	0.849	854.287
8.00E+15	0.5	0.4	3.00E+17	0.726	225.225

8.00E+15	0.5	0.4	4.00E+17	0.805	312.164
8.00E+15	0.5	0.4	5.00E+17	0.838	415.163
8.00E+15	0.5	0.4	6.00E+17	0.885	530.020
8.00E+15	0.5	0.4	7.00E+17	0.902	460.406
8.00E+15	0.5	0.4	8.00E+17	0.928	766.888
8.00E+15	0.5	0.4	9.00E+17	0.951	879.531
8.00E+15	0.5	0.4	1.00E+18	0.972	986.738
8.00E+15	0.6	0.15	3.00E+17	0.394	106.756
8.00E+15	0.6	0.15	4.00E+17	0.430	125.986
8.00E+15	0.6	0.15	5.00E+17	0.479	143.374
8.00E+15	0.6	0.15	6.00E+17	0.532	155.002
8.00E+15	0.6	0.15	7.00E+17	0.564	203.642
8.00E+15	0.6	0.15	8.00E+17	0.588	234.521
8.00E+15	0.6	0.15	9.00E+17	0.606	267.455
8.00E+15	0.6	0.15	1.00E+18	0.618	303.059
8.00E+15	0.6	0.2	3.00E+17	0.486	129.438
8.00E+15	0.6	0.2	4.00E+17	0.569	161.439
8.00E+15	0.6	0.2	5.00E+17	0.631	199.958
8.00E+15	0.6	0.2	6.00E+17	0.656	242.219
8.00E+15	0.6	0.2	7.00E+17	0.688	288.938
8.00E+15	0.6	0.2	8.00E+17	0.714	340.510
8.00E+15	0.6	0.2	9.00E+17	0.721	396.762
8.00E+15	0.6	0.2	1.00E+18	0.740	457.485
8.00E+15	0.6	0.25	3.00E+17	0.627	154.867
8.00E+15	0.6	0.25	4.00E+17	0.688	201.315
8.00E+15	0.6	0.25	5.00E+17	0.746	257.285
8.00E+15	0.6	0.25	6.00E+17	0.794	319.295
8.00E+15	0.6	0.25	7.00E+17	0.822	388.931
8.00E+15	0.6	0.25	8.00E+17	0.843	465.727
8.00E+15	0.6	0.25	9.00E+17	0.848	546.697
8.00E+15	0.6	0.25	1.00E+18	0.887	627.515
8.00E+15	0.6	0.3	3.00E+17	0.744	184.326
8.00E+15	0.6	0.3	4.00E+17	0.839	247.357
8.00E+15	0.6	0.3	5.00E+17	0.910	221.519
8.00E+15	0.6	0.3	6.00E+17	0.939	406.494
8.00E+15	0.6	0.3	7.00E+17	0.987	501.661
8.00E+15	0.6	0.3	8.00E+17	1.004	599.451
8.00E+15	0.6	0.3	9.00E+17	1.043	695.970
8.00E+15	0.6	0.3	1.00E+18	1.057	791.090
8.00E+15	0.6	0.35	3.00E+17	0.876	214.181
8.00E+15	0.6	0.35	4.00E+17	0.985	295.586
8.00E+15	0.6	0.35	5.00E+17	1.089	391.958
8.00E+15	0.6	0.35	6.00E+17	1.114	502.127
8.00E+15	0.6	0.35	7.00E+17	1.159	615.891
8.00E+15	0.6	0.35	8.00E+17	1.199	728.473
8.00E+15	0.6	0.35	9.00E+17	1.230	839.007
8.00E+15	0.6	0.35	1.00E+18	1.261	945.608
8.00E+15	0.6	0.4	3.00E+17	1.042	247.059
8.00E+15	0.6	0.4	4.00E+17	1.154	348.609
8.00E+15	0.6	0.4	5.00E+17	1.295	426.646
8.00E+15	0.6	0.4	6.00E+17	1.304	596.647
8.00E+15	0.6	0.4	7.00E+17	1.373	727.716
8.00E+15	0.6	0.4	8.00E+17	1.410	853.835
8.00E+15	0.6	0.4	9.00E+17	1.464	975.068
8.00E+15	0.6	0.4	1.00E+18	1.500	1089.290
1.00E+16	0.2	0.15	3.00E+17	0.226	48.480
1.00E+16	0.2	0.15	4.00E+17	0.232	56.450
1.00E+16	0.2	0.15	5.00E+17	0.236	64.848

1.00E+16	0.2	0.15	6.00E+17	0.240	73.674
1.00E+16	0.2	0.15	7.00E+17	0.242	82.807
1.00E+16	0.2	0.15	8.00E+17	0.243	92.101
1.00E+16	0.2	0.15	9.00E+17	0.244	101.924
1.00E+16	0.2	0.15	1.00E+18	0.245	111.957
1.00E+16	0.2	0.2	3.00E+17	0.238	56.523
1.00E+16	0.2	0.2	4.00E+17	0.244	67.863
1.00E+16	0.2	0.2	5.00E+17	0.248	79.910
1.00E+16	0.2	0.2	6.00E+17	0.250	91.733
1.00E+16	0.2	0.2	7.00E+17	0.252	105.526
1.00E+16	0.2	0.2	8.00E+17	0.255	119.146
1.00E+16	0.2	0.2	9.00E+17	0.256	132.059
1.00E+16	0.2	0.2	1.00E+18	0.258	147.484
1.00E+16	0.2	0.25	3.00E+17	0.250	64.493
1.00E+16	0.2	0.25	4.00E+17	0.256	79.253
1.00E+16	0.2	0.25	5.00E+17	0.259	94.696
1.00E+16	0.2	0.25	6.00E+17	0.263	111.280
1.00E+16	0.2	0.25	7.00E+17	0.264	128.323
1.00E+16	0.2	0.25	8.00E+17	0.266	145.949
1.00E+16	0.2	0.25	9.00E+17	0.268	164.235
1.00E+16	0.2	0.25	1.00E+18	0.269	183.787
1.00E+16	0.2	0.3	3.00E+17	0.261	72.408
1.00E+16	0.2	0.3	4.00E+17	0.265	90.307
1.00E+16	0.2	0.3	5.00E+17	0.269	109.685
1.00E+16	0.2	0.3	6.00E+17	0.273	129.810
1.00E+16	0.2	0.3	7.00E+17	0.275	150.776
1.00E+16	0.2	0.3	8.00E+17	0.276	171.895
1.00E+16	0.2	0.3	9.00E+17	0.278	197.137
1.00E+16	0.2	0.3	1.00E+18	0.278	223.502
1.00E+16	0.2	0.35	3.00E+17	0.270	80.054
1.00E+16	0.2	0.35	4.00E+17	0.274	101.250
1.00E+16	0.2	0.35	5.00E+17	0.279	124.203
1.00E+16	0.2	0.35	6.00E+17	0.281	147.921
1.00E+16	0.2	0.35	7.00E+17	0.285	173.394
1.00E+16	0.2	0.35	8.00E+17	0.287	201.540
1.00E+16	0.2	0.35	9.00E+17	0.288	232.800
1.00E+16	0.2	0.35	1.00E+18	0.289	265.088
1.00E+16	0.2	0.4	3.00E+17	0.279	87.499
1.00E+16	0.2	0.4	4.00E+17	0.284	112.187
1.00E+16	0.2	0.4	5.00E+17	0.289	138.447
1.00E+16	0.2	0.4	6.00E+17	0.291	166.401
1.00E+16	0.2	0.4	7.00E+17	0.297	197.579
1.00E+16	0.2	0.4	8.00E+17	0.297	232.678
1.00E+16	0.2	0.4	9.00E+17	0.300	271.448
1.00E+16	0.2	0.4	1.00E+18	0.302	312.477
1.00E+16	0.25	0.15	3.00E+17	0.232	41.417
1.00E+16	0.25	0.15	4.00E+17	0.240	49.402
1.00E+16	0.25	0.15	5.00E+17	0.245	57.685
1.00E+16	0.25	0.15	6.00E+17	0.249	66.593
1.00E+16	0.25	0.15	7.00E+17	0.253	75.822
1.00E+16	0.25	0.15	8.00E+17	0.256	85.300
1.00E+16	0.25	0.15	9.00E+17	0.257	95.129
1.00E+16	0.25	0.15	1.00E+18	0.257	105.298
1.00E+16	0.25	0.2	3.00E+17	0.246	49.909
1.00E+16	0.25	0.2	4.00E+17	0.256	61.445
1.00E+16	0.25	0.2	5.00E+17	0.261	73.877
1.00E+16	0.25	0.2	6.00E+17	0.264	86.829
1.00E+16	0.25	0.2	7.00E+17	0.266	100.430

1.00E+16	0.25	0.2	8.00E+17	0.270	114.709
1.00E+16	0.25	0.2	9.00E+17	0.271	129.265
1.00E+16	0.25	0.2	1.00E+18	0.273	144.469
1.00E+16	0.25	0.25	3.00E+17	0.263	57.845
1.00E+16	0.25	0.25	4.00E+17	0.269	72.963
1.00E+16	0.25	0.25	5.00E+17	0.275	88.922
1.00E+16	0.25	0.25	6.00E+17	0.275	105.970
1.00E+16	0.25	0.25	7.00E+17	0.279	123.737
1.00E+16	0.25	0.25	8.00E+17	0.281	142.272
1.00E+16	0.25	0.25	9.00E+17	0.284	161.505
1.00E+16	0.25	0.25	1.00E+18	0.285	182.426
1.00E+16	0.25	0.3	3.00E+17	0.275	65.802
1.00E+16	0.25	0.3	4.00E+17	0.281	84.230
1.00E+16	0.25	0.3	5.00E+17	0.288	103.978
1.00E+16	0.25	0.3	6.00E+17	0.289	124.985
1.00E+16	0.25	0.3	7.00E+17	0.293	146.952
1.00E+16	0.25	0.3	8.00E+17	0.296	170.445
1.00E+16	0.25	0.3	9.00E+17	0.298	196.347
1.00E+16	0.25	0.3	1.00E+18	0.302	224.999
1.00E+16	0.25	0.35	3.00E+17	0.285	73.599
1.00E+16	0.25	0.35	4.00E+17	0.295	95.413
1.00E+16	0.25	0.35	5.00E+17	0.299	119.046
1.00E+16	0.25	0.35	6.00E+17	0.304	143.966
1.00E+16	0.25	0.35	7.00E+17	0.305	171.010
1.00E+16	0.25	0.35	8.00E+17	0.314	201.117
1.00E+16	0.25	0.35	9.00E+17	0.311	235.133
1.00E+16	0.25	0.35	1.00E+18	0.317	272.027
1.00E+16	0.25	0.4	3.00E+17	0.298	81.314
1.00E+16	0.25	0.4	4.00E+17	0.306	106.798
1.00E+16	0.25	0.4	5.00E+17	0.316	134.116
1.00E+16	0.25	0.4	6.00E+17	0.320	163.655
1.00E+16	0.25	0.4	7.00E+17	0.323	197.129
1.00E+16	0.25	0.4	8.00E+17	0.331	235.434
1.00E+16	0.25	0.4	9.00E+17	0.331	277.710
1.00E+16	0.25	0.4	1.00E+18	0.333	323.966
1.00E+16	0.3	0.15	3.00E+17	0.238	43.788
1.00E+16	0.3	0.15	4.00E+17	0.250	52.678
1.00E+16	0.3	0.15	5.00E+17	0.256	62.176
1.00E+16	0.3	0.15	6.00E+17	0.262	72.229
1.00E+16	0.3	0.15	7.00E+17	0.266	82.658
1.00E+16	0.3	0.15	8.00E+17	0.269	93.457
1.00E+16	0.3	0.15	9.00E+17	0.271	104.714
1.00E+16	0.3	0.15	1.00E+18	0.272	116.316
1.00E+16	0.3	0.2	3.00E+17	0.260	53.215
1.00E+16	0.3	0.2	4.00E+17	0.269	66.316
1.00E+16	0.3	0.2	5.00E+17	0.275	80.278
1.00E+16	0.3	0.2	6.00E+17	0.280	94.930
1.00E+16	0.3	0.2	7.00E+17	0.283	110.221
1.00E+16	0.3	0.2	8.00E+17	0.285	125.217
1.00E+16	0.3	0.2	9.00E+17	0.288	143.317
1.00E+16	0.3	0.2	1.00E+18	0.290	160.639
1.00E+16	0.3	0.25	3.00E+17	0.278	62.433
1.00E+16	0.3	0.25	4.00E+17	0.286	79.419
1.00E+16	0.3	0.25	5.00E+17	0.294	97.607
1.00E+16	0.3	0.25	6.00E+17	0.297	118.466
1.00E+16	0.3	0.25	7.00E+17	0.301	137.164
1.00E+16	0.3	0.25	8.00E+17	0.305	160.331
1.00E+16	0.3	0.25	9.00E+17	0.308	181.678

1.00E+16	0.3	0.25	1.00E+18	0.309	206.886
1.00E+16	0.3	0.3	3.00E+17	0.292	71.574
1.00E+16	0.3	0.3	4.00E+17	0.303	92.534
1.00E+16	0.3	0.3	5.00E+17	0.311	115.172
1.00E+16	0.3	0.3	6.00E+17	0.316	139.109
1.00E+16	0.3	0.3	7.00E+17	0.322	166.745
1.00E+16	0.3	0.3	8.00E+17	0.324	193.295
1.00E+16	0.3	0.3	9.00E+17	0.330	225.314
1.00E+16	0.3	0.3	1.00E+18	0.330	263.560
1.00E+16	0.3	0.35	3.00E+17	0.306	80.517
1.00E+16	0.3	0.35	4.00E+17	0.321	105.638
1.00E+16	0.3	0.35	5.00E+17	0.331	132.600
1.00E+16	0.3	0.35	6.00E+17	0.336	161.731
1.00E+16	0.3	0.35	7.00E+17	0.344	197.159
1.00E+16	0.3	0.35	8.00E+17	0.347	220.891
1.00E+16	0.3	0.35	9.00E+17	0.354	277.416
1.00E+16	0.3	0.35	1.00E+18	0.354	319.575
1.00E+16	0.3	0.4	3.00E+17	0.320	90.815
1.00E+16	0.3	0.4	4.00E+17	0.341	118.735
1.00E+16	0.3	0.4	5.00E+17	0.352	150.284
1.00E+16	0.3	0.4	6.00E+17	0.360	186.274
1.00E+16	0.3	0.4	7.00E+17	0.368	228.176
1.00E+16	0.3	0.4	8.00E+17	0.373	278.712
1.00E+16	0.3	0.4	9.00E+17	0.378	327.598
1.00E+16	0.3	0.4	1.00E+18	0.380	387.963
1.00E+16	0.35	0.15	3.00E+17	0.249	46.609
1.00E+16	0.35	0.15	4.00E+17	0.261	56.419
1.00E+16	0.35	0.15	5.00E+17	0.268	67.080
1.00E+16	0.35	0.15	6.00E+17	0.275	78.212
1.00E+16	0.35	0.15	7.00E+17	0.279	89.775
1.00E+16	0.35	0.15	8.00E+17	0.284	101.941
1.00E+16	0.35	0.15	9.00E+17	0.286	114.476
1.00E+16	0.35	0.15	1.00E+18	0.290	127.392
1.00E+16	0.35	0.2	3.00E+17	0.273	57.205
1.00E+16	0.35	0.2	4.00E+17	0.284	71.867
1.00E+16	0.35	0.2	5.00E+17	0.296	87.397
1.00E+16	0.35	0.2	6.00E+17	0.299	104.001
1.00E+16	0.35	0.2	7.00E+17	0.304	121.265
1.00E+16	0.35	0.2	8.00E+17	0.306	139.317
1.00E+16	0.35	0.2	9.00E+17	0.313	158.231
1.00E+16	0.35	0.2	1.00E+18	0.316	178.856
1.00E+16	0.35	0.25	3.00E+17	0.290	67.690
1.00E+16	0.35	0.25	4.00E+17	0.313	86.653
1.00E+16	0.35	0.25	5.00E+17	0.321	107.284
1.00E+16	0.35	0.25	6.00E+17	0.325	128.945
1.00E+16	0.35	0.25	7.00E+17	0.336	151.764
1.00E+16	0.35	0.25	8.00E+17	0.332	177.044
1.00E+16	0.35	0.25	9.00E+17	0.339	204.739
1.00E+16	0.35	0.25	1.00E+18	0.344	235.556
1.00E+16	0.35	0.3	3.00E+17	0.318	78.017
1.00E+16	0.35	0.3	4.00E+17	0.332	101.757
1.00E+16	0.35	0.3	5.00E+17	0.348	127.196
1.00E+16	0.35	0.3	6.00E+17	0.358	154.441
1.00E+16	0.35	0.3	7.00E+17	0.358	185.083
1.00E+16	0.35	0.3	8.00E+17	0.365	219.766
1.00E+16	0.35	0.3	9.00E+17	0.373	258.228
1.00E+16	0.35	0.3	1.00E+18	0.375	300.528
1.00E+16	0.35	0.35	3.00E+17	0.336	88.279

1.00E+16	0.35	0.35	4.00E+17	0.366	116.708
1.00E+16	0.35	0.35	5.00E+17	0.373	147.268
1.00E+16	0.35	0.35	6.00E+17	0.379	182.162
1.00E+16	0.35	0.35	7.00E+17	0.391	222.460
1.00E+16	0.35	0.35	8.00E+17	0.400	267.727
1.00E+16	0.35	0.35	9.00E+17	0.402	317.635
1.00E+16	0.35	0.35	1.00E+18	0.403	371.614
1.00E+16	0.35	0.4	3.00E+17	0.356	98.870
1.00E+16	0.35	0.4	4.00E+17	0.386	131.922
1.00E+16	0.35	0.4	5.00E+17	0.399	169.149
1.00E+16	0.35	0.4	6.00E+17	0.415	212.864
1.00E+16	0.35	0.4	7.00E+17	0.416	262.557
1.00E+16	0.35	0.4	8.00E+17	0.432	319.061
1.00E+16	0.35	0.4	9.00E+17	0.428	382.495
1.00E+16	0.35	0.4	1.00E+18	0.437	451.961
1.00E+16	0.4	0.15	3.00E+17	0.260	48.298
1.00E+16	0.4	0.15	4.00E+17	0.274	59.035
1.00E+16	0.4	0.15	5.00E+17	0.284	70.549
1.00E+16	0.4	0.15	6.00E+17	0.293	82.607
1.00E+16	0.4	0.15	7.00E+17	0.301	95.285
1.00E+16	0.4	0.15	8.00E+17	0.310	108.565
1.00E+16	0.4	0.15	9.00E+17	0.312	122.161
1.00E+16	0.4	0.15	1.00E+18	0.315	136.355
1.00E+16	0.4	0.2	3.00E+17	0.290	59.872
1.00E+16	0.4	0.2	4.00E+17	0.308	75.667
1.00E+16	0.4	0.2	5.00E+17	0.325	92.629
1.00E+16	0.4	0.2	6.00E+17	0.327	110.714
1.00E+16	0.4	0.2	7.00E+17	0.336	129.566
1.00E+16	0.4	0.2	8.00E+17	0.340	149.294
1.00E+16	0.4	0.2	9.00E+17	0.348	170.768
1.00E+16	0.4	0.2	1.00E+18	0.355	192.689
1.00E+16	0.4	0.25	3.00E+17	0.317	71.369
1.00E+16	0.4	0.25	4.00E+17	0.343	92.063
1.00E+16	0.4	0.25	5.00E+17	0.357	114.503
1.00E+16	0.4	0.25	6.00E+17	0.362	138.257
1.00E+16	0.4	0.25	7.00E+17	0.372	164.163
1.00E+16	0.4	0.25	8.00E+17	0.375	192.856
1.00E+16	0.4	0.25	9.00E+17	0.384	225.260
1.00E+16	0.4	0.25	1.00E+18	0.387	260.404
1.00E+16	0.4	0.3	3.00E+17	0.348	82.791
1.00E+16	0.4	0.3	4.00E+17	0.375	108.783
1.00E+16	0.4	0.3	5.00E+17	0.393	136.761
1.00E+16	0.4	0.3	6.00E+17	0.400	167.799
1.00E+16	0.4	0.3	7.00E+17	0.407	203.225
1.00E+16	0.4	0.3	8.00E+17	0.418	243.254
1.00E+16	0.4	0.3	9.00E+17	0.421	287.924
1.00E+16	0.4	0.3	1.00E+18	0.427	336.684
1.00E+16	0.4	0.35	3.00E+17	0.376	94.277
1.00E+16	0.4	0.35	4.00E+17	0.412	125.464
1.00E+16	0.4	0.35	5.00E+17	0.425	159.214
1.00E+16	0.4	0.35	6.00E+17	0.433	200.370
1.00E+16	0.4	0.35	7.00E+17	0.448	247.110
1.00E+16	0.4	0.35	8.00E+17	0.452	299.738
1.00E+16	0.4	0.35	9.00E+17	0.459	356.512
1.00E+16	0.4	0.35	1.00E+18	0.466	419.739
1.00E+16	0.4	0.4	3.00E+17	0.406	106.008
1.00E+16	0.4	0.4	4.00E+17	0.447	142.547
1.00E+16	0.4	0.4	5.00E+17	0.464	185.656

1.00E+16	0.4	0.4	6.00E+17	0.473	236.883
1.00E+16	0.4	0.4	7.00E+17	0.478	295.854
1.00E+16	0.4	0.4	8.00E+17	0.496	362.501
1.00E+16	0.4	0.4	9.00E+17	0.504	434.646
1.00E+16	0.4	0.4	1.00E+18	0.513	515.626
1.00E+16	0.45	0.15	3.00E+17	0.273	50.005
1.00E+16	0.45	0.15	4.00E+17	0.291	61.590
1.00E+16	0.45	0.15	5.00E+17	0.304	73.948
1.00E+16	0.45	0.15	6.00E+17	0.318	87.005
1.00E+16	0.45	0.15	7.00E+17	0.330	100.749
1.00E+16	0.45	0.15	8.00E+17	0.341	115.010
1.00E+16	0.45	0.15	9.00E+17	0.344	129.844
1.00E+16	0.45	0.15	1.00E+18	0.352	145.224
1.00E+16	0.45	0.2	3.00E+17	0.310	62.530
1.00E+16	0.45	0.2	4.00E+17	0.339	79.533
1.00E+16	0.45	0.2	5.00E+17	0.355	97.947
1.00E+16	0.45	0.2	6.00E+17	0.363	117.558
1.00E+16	0.45	0.2	7.00E+17	0.373	138.087
1.00E+16	0.45	0.2	8.00E+17	0.384	160.281
1.00E+16	0.45	0.2	9.00E+17	0.386	184.584
1.00E+16	0.45	0.2	1.00E+18	0.397	211.464
1.00E+16	0.45	0.25	3.00E+17	0.346	75.066
1.00E+16	0.45	0.25	4.00E+17	0.383	97.547
1.00E+16	0.45	0.25	5.00E+17	0.388	121.834
1.00E+16	0.45	0.25	6.00E+17	0.405	147.008
1.00E+16	0.45	0.25	7.00E+17	0.423	170.493
1.00E+16	0.45	0.25	8.00E+17	0.428	210.219
1.00E+16	0.45	0.25	9.00E+17	0.432	246.982
1.00E+16	0.45	0.25	1.00E+18	0.443	287.375
1.00E+16	0.45	0.3	3.00E+17	0.395	87.543
1.00E+16	0.45	0.3	4.00E+17	0.432	115.754
1.00E+16	0.45	0.3	5.00E+17	0.445	146.421
1.00E+16	0.45	0.3	6.00E+17	0.461	181.664
1.00E+16	0.45	0.3	7.00E+17	0.473	222.566
1.00E+16	0.45	0.3	8.00E+17	0.478	268.321
1.00E+16	0.45	0.3	9.00E+17	0.483	319.265
1.00E+16	0.45	0.3	1.00E+18	0.493	375.161
1.00E+16	0.45	0.35	3.00E+17	0.435	100.230
1.00E+16	0.45	0.35	4.00E+17	0.480	134.133
1.00E+16	0.45	0.35	5.00E+17	0.487	173.352
1.00E+16	0.45	0.35	6.00E+17	0.504	219.854
1.00E+16	0.45	0.35	7.00E+17	0.529	273.311
1.00E+16	0.45	0.35	8.00E+17	0.537	333.516
1.00E+16	0.45	0.35	9.00E+17	0.539	400.700
1.00E+16	0.45	0.35	1.00E+18	0.552	474.529
1.00E+16	0.45	0.4	3.00E+17	0.472	113.049
1.00E+16	0.45	0.4	4.00E+17	0.525	153.955
1.00E+16	0.45	0.4	5.00E+17	0.551	203.302
1.00E+16	0.45	0.4	6.00E+17	0.567	262.035
1.00E+16	0.45	0.4	7.00E+17	0.580	329.714
1.00E+16	0.45	0.4	8.00E+17	0.596	406.231
1.00E+16	0.45	0.4	9.00E+17	0.601	489.375
1.00E+16	0.45	0.4	1.00E+18	0.607	584.051
1.00E+16	0.5	0.15	3.00E+17	0.288	92.730
1.00E+16	0.5	0.15	4.00E+17	0.308	110.016
1.00E+16	0.5	0.15	5.00E+17	0.327	127.983
1.00E+16	0.5	0.15	6.00E+17	0.349	148.350
1.00E+16	0.5	0.15	7.00E+17	0.364	168.375

1.00E+16	0.5	0.15	8.00E+17	0.379	190.025
1.00E+16	0.5	0.15	9.00E+17	0.378	135.423
1.00E+16	0.5	0.15	1.00E+18	0.392	140.280
1.00E+16	0.5	0.2	3.00E+17	0.332	112.529
1.00E+16	0.5	0.2	4.00E+17	0.375	138.435
1.00E+16	0.5	0.2	5.00E+17	0.394	163.324
1.00E+16	0.5	0.2	6.00E+17	0.410	187.679
1.00E+16	0.5	0.2	7.00E+17	0.415	228.258
1.00E+16	0.5	0.2	8.00E+17	0.433	264.722
1.00E+16	0.5	0.2	9.00E+17	0.432	304.448
1.00E+16	0.5	0.2	1.00E+18	0.447	347.158
1.00E+16	0.5	0.25	3.00E+17	0.385	131.872
1.00E+16	0.5	0.25	4.00E+17	0.433	166.361
1.00E+16	0.5	0.25	5.00E+17	0.453	204.955
1.00E+16	0.5	0.25	6.00E+17	0.465	248.714
1.00E+16	0.5	0.25	7.00E+17	0.485	265.216
1.00E+16	0.5	0.25	8.00E+17	0.491	290.404
1.00E+16	0.5	0.25	9.00E+17	0.511	316.601
1.00E+16	0.5	0.25	1.00E+18	0.516	469.824
1.00E+16	0.5	0.3	3.00E+17	0.447	152.807
1.00E+16	0.5	0.3	4.00E+17	0.480	196.752
1.00E+16	0.5	0.3	5.00E+17	0.510	179.639
1.00E+16	0.5	0.3	6.00E+17	0.535	308.439
1.00E+16	0.5	0.3	7.00E+17	0.549	375.234
1.00E+16	0.5	0.3	8.00E+17	0.563	445.053
1.00E+16	0.5	0.3	9.00E+17	0.564	529.896
1.00E+16	0.5	0.35	3.00E+17	0.501	173.472
1.00E+16	0.5	0.35	4.00E+17	0.527	229.923
1.00E+16	0.5	0.35	5.00E+17	0.573	296.952
1.00E+16	0.5	0.35	6.00E+17	0.607	373.723
1.00E+16	0.5	0.35	7.00E+17	0.613	301.931
1.00E+16	0.5	0.35	8.00E+17	0.637	556.767
1.00E+16	0.5	0.35	9.00E+17	0.646	657.757
1.00E+16	0.5	0.35	1.00E+18	0.663	756.887
1.00E+16	0.5	0.4	3.00E+17	0.560	189.528
1.00E+16	0.5	0.4	4.00E+17	0.619	262.364
1.00E+16	0.5	0.4	5.00E+17	0.656	345.514
1.00E+16	0.5	0.4	6.00E+17	0.668	440.988
1.00E+16	0.5	0.4	7.00E+17	0.689	549.293
1.00E+16	0.5	0.4	8.00E+17	0.720	416.535
1.00E+16	0.5	0.4	9.00E+17	0.731	782.218
1.00E+16	0.5	0.4	1.00E+18	0.736	890.114
1.00E+16	0.6	0.15	3.00E+17	0.325	95.596
1.00E+16	0.6	0.15	4.00E+17	0.352	111.881
1.00E+16	0.6	0.15	5.00E+17	0.387	123.078
1.00E+16	0.6	0.15	6.00E+17	0.425	134.953
1.00E+16	0.6	0.15	7.00E+17	0.452	141.704
1.00E+16	0.6	0.15	8.00E+17	0.476	151.135
1.00E+16	0.6	0.15	9.00E+17	0.479	228.135
1.00E+16	0.6	0.15	1.00E+18	0.502	257.921
1.00E+16	0.6	0.2	3.00E+17	0.395	117.507
1.00E+16	0.6	0.2	4.00E+17	0.459	144.883
1.00E+16	0.6	0.2	5.00E+17	0.498	174.942
1.00E+16	0.6	0.2	6.00E+17	0.523	208.762
1.00E+16	0.6	0.2	7.00E+17	0.552	246.490
1.00E+16	0.6	0.2	8.00E+17	0.564	288.218
1.00E+16	0.6	0.2	9.00E+17	0.579	333.524
1.00E+16	0.6	0.2	1.00E+18	0.595	382.624

1.00E+16	0.6	0.25	3.00E+17	0.495	139.782
1.00E+16	0.6	0.25	4.00E+17	0.569	177.207
1.00E+16	0.6	0.25	5.00E+17	0.602	198.827
1.00E+16	0.6	0.25	6.00E+17	0.630	271.084
1.00E+16	0.6	0.25	7.00E+17	0.654	323.673
1.00E+16	0.6	0.25	8.00E+17	0.673	389.274
1.00E+16	0.6	0.25	9.00E+17	0.700	453.129
1.00E+16	0.6	0.25	1.00E+18	0.715	526.737
1.00E+16	0.6	0.3	3.00E+17	0.595	162.455
1.00E+16	0.6	0.3	4.00E+17	0.666	212.951
1.00E+16	0.6	0.3	5.00E+17	0.705	269.575
1.00E+16	0.6	0.3	6.00E+17	0.721	341.292
1.00E+16	0.6	0.3	7.00E+17	0.783	240.094
1.00E+16	0.6	0.3	8.00E+17	0.810	502.814
1.00E+16	0.6	0.3	9.00E+17	0.828	598.123
1.00E+16	0.6	0.3	1.00E+18	0.837	693.245
1.00E+16	0.6	0.35	3.00E+17	0.680	186.191
1.00E+16	0.6	0.35	4.00E+17	0.777	251.582
1.00E+16	0.6	0.35	5.00E+17	0.848	329.334
1.00E+16	0.6	0.35	6.00E+17	0.882	418.832
1.00E+16	0.6	0.35	7.00E+17	0.909	518.389
1.00E+16	0.6	0.35	8.00E+17	0.943	631.046
1.00E+16	0.6	0.35	9.00E+17	0.960	741.413
1.00E+16	0.6	0.35	1.00E+18	0.989	848.411
1.00E+16	0.6	0.4	3.00E+17	0.812	212.572
1.00E+16	0.6	0.4	4.00E+17	0.900	294.188
1.00E+16	0.6	0.4	5.00E+17	1.008	388.064
1.00E+16	0.6	0.4	6.00E+17	1.027	411.435
1.00E+16	0.6	0.4	7.00E+17	1.062	630.552
1.00E+16	0.6	0.4	8.00E+17	1.098	756.772
1.00E+16	0.6	0.4	9.00E+17	1.138	878.753
1.00E+16	0.6	0.4	1.00E+18	1.161	993.067
9.00E+15	0.2	0.15	3.00E+17	0.547	68.486
9.00E+15	0.2	0.15	4.00E+17	0.571	80.273
9.00E+15	0.2	0.15	5.00E+17	0.584	93.327
9.00E+15	0.2	0.15	6.00E+17	0.599	108.019
9.00E+15	0.2	0.15	7.00E+17	0.609	123.968
9.00E+15	0.2	0.15	8.00E+17	0.614	141.218
9.00E+15	0.2	0.15	9.00E+17	0.617	159.951
9.00E+15	0.2	0.15	1.00E+18	0.620	179.838
9.00E+15	0.2	0.2	3.00E+17	0.596	80.203
9.00E+15	0.2	0.2	4.00E+17	0.615	98.240
9.00E+15	0.2	0.2	5.00E+17	0.636	118.716
9.00E+15	0.2	0.2	6.00E+17	0.641	141.593
9.00E+15	0.2	0.2	7.00E+17	0.651	167.545
9.00E+15	0.2	0.2	8.00E+17	0.659	195.480
9.00E+15	0.2	0.2	9.00E+17	0.667	225.722
9.00E+15	0.2	0.2	1.00E+18	0.666	258.044
9.00E+15	0.2	0.25	3.00E+17	0.637	92.850
9.00E+15	0.2	0.25	4.00E+17	0.663	117.952
9.00E+15	0.2	0.25	5.00E+17	0.681	147.008
9.00E+15	0.2	0.25	6.00E+17	0.693	179.881
9.00E+15	0.2	0.25	7.00E+17	0.702	216.327
9.00E+15	0.2	0.25	8.00E+17	0.709	256.036
9.00E+15	0.2	0.25	9.00E+17	0.716	297.271
9.00E+15	0.2	0.25	1.00E+18	0.719	338.464
9.00E+15	0.2	0.3	3.00E+17	0.678	106.369
9.00E+15	0.2	0.3	4.00E+17	0.714	139.248

9.00E+15	0.2	0.3	5.00E+17	0.737	177.532
9.00E+15	0.2	0.3	6.00E+17	0.747	221.040
9.00E+15	0.2	0.3	7.00E+17	0.763	268.933
9.00E+15	0.2	0.3	8.00E+17	0.769	317.649
9.00E+15	0.2	0.3	9.00E+17	0.777	366.592
9.00E+15	0.2	0.3	1.00E+18	0.784	415.397
9.00E+15	0.2	0.35	3.00E+17	0.722	120.509
9.00E+15	0.2	0.35	4.00E+17	0.769	161.695
9.00E+15	0.2	0.35	5.00E+17	0.794	209.940
9.00E+15	0.2	0.35	6.00E+17	0.806	264.377
9.00E+15	0.2	0.35	7.00E+17	0.822	320.760
9.00E+15	0.2	0.35	8.00E+17	0.835	376.930
9.00E+15	0.2	0.35	9.00E+17	0.845	433.133
9.00E+15	0.2	0.35	1.00E+18	0.853	489.405
9.00E+15	0.2	0.4	3.00E+17	0.774	135.276
9.00E+15	0.2	0.4	4.00E+17	0.830	185.479
9.00E+15	0.2	0.4	5.00E+17	0.858	244.569
9.00E+15	0.2	0.4	6.00E+17	0.875	307.875
9.00E+15	0.2	0.4	7.00E+17	0.888	371.330
9.00E+15	0.2	0.4	8.00E+17	0.906	434.941
9.00E+15	0.2	0.4	9.00E+17	0.915	498.300
9.00E+15	0.2	0.4	1.00E+18	0.930	561.655
9.00E+15	0.25	0.15	3.00E+17	0.568	58.116
9.00E+15	0.25	0.15	4.00E+17	0.592	69.580
9.00E+15	0.25	0.15	5.00E+17	0.610	82.239
9.00E+15	0.25	0.15	6.00E+17	0.626	96.558
9.00E+15	0.25	0.15	7.00E+17	0.641	112.377
9.00E+15	0.25	0.15	8.00E+17	0.647	129.772
9.00E+15	0.25	0.15	9.00E+17	0.656	148.666
9.00E+15	0.25	0.15	1.00E+18	0.661	168.973
9.00E+15	0.25	0.2	3.00E+17	0.623	70.220
9.00E+15	0.25	0.2	4.00E+17	0.661	88.080
9.00E+15	0.25	0.2	5.00E+17	0.673	109.126
9.00E+15	0.25	0.2	6.00E+17	0.686	133.015
9.00E+15	0.25	0.2	7.00E+17	0.702	159.723
9.00E+15	0.25	0.2	8.00E+17	0.713	189.178
9.00E+15	0.25	0.2	9.00E+17	0.717	221.396
9.00E+15	0.25	0.2	1.00E+18	0.716	255.979
9.00E+15	0.25	0.25	3.00E+17	0.681	82.508
9.00E+15	0.25	0.25	4.00E+17	0.719	107.910
9.00E+15	0.25	0.25	5.00E+17	0.735	137.720
9.00E+15	0.25	0.25	6.00E+17	0.754	171.962
9.00E+15	0.25	0.25	7.00E+17	0.767	210.508
9.00E+15	0.25	0.25	8.00E+17	0.780	243.141
9.00E+15	0.25	0.25	9.00E+17	0.780	270.123
9.00E+15	0.25	0.25	1.00E+18	0.785	340.734
9.00E+15	0.25	0.3	3.00E+17	0.734	95.914
9.00E+15	0.25	0.3	4.00E+17	0.783	129.468
9.00E+15	0.25	0.3	5.00E+17	0.801	169.253
9.00E+15	0.25	0.3	6.00E+17	0.818	215.036
9.00E+15	0.25	0.3	7.00E+17	0.839	265.953
9.00E+15	0.25	0.3	8.00E+17	0.833	318.115
9.00E+15	0.25	0.3	9.00E+17	0.852	370.246
9.00E+15	0.25	0.3	1.00E+18	0.854	422.421
9.00E+15	0.25	0.35	3.00E+17	0.793	110.153
9.00E+15	0.25	0.35	4.00E+17	0.843	152.761
9.00E+15	0.25	0.35	5.00E+17	0.871	203.592
9.00E+15	0.25	0.35	6.00E+17	0.889	261.465

9.00E+15	0.25	0.35	7.00E+17	0.906	321.498
9.00E+15	0.25	0.35	8.00E+17	0.923	381.600
9.00E+15	0.25	0.35	9.00E+17	0.931	441.562
9.00E+15	0.25	0.35	1.00E+18	0.944	501.530
9.00E+15	0.25	0.4	3.00E+17	0.854	125.556
9.00E+15	0.25	0.4	4.00E+17	0.920	177.881
9.00E+15	0.25	0.4	5.00E+17	0.941	240.513
9.00E+15	0.25	0.4	6.00E+17	0.974	307.722
9.00E+15	0.25	0.4	7.00E+17	0.993	375.826
9.00E+15	0.25	0.4	8.00E+17	1.010	443.492
9.00E+15	0.25	0.4	9.00E+17	1.023	511.153
9.00E+15	0.25	0.4	1.00E+18	1.027	578.747
9.00E+15	0.3	0.15	3.00E+17	0.587	61.767
9.00E+15	0.3	0.15	4.00E+17	0.615	74.928
9.00E+15	0.3	0.15	5.00E+17	0.644	89.962
9.00E+15	0.3	0.15	6.00E+17	0.669	107.098
9.00E+15	0.3	0.15	7.00E+17	0.686	126.157
9.00E+15	0.3	0.15	8.00E+17	0.691	146.992
9.00E+15	0.3	0.15	9.00E+17	0.699	169.716
9.00E+15	0.3	0.15	1.00E+18	0.703	194.329
9.00E+15	0.3	0.2	3.00E+17	0.651	75.590
9.00E+15	0.3	0.2	4.00E+17	0.700	96.894
9.00E+15	0.3	0.2	5.00E+17	0.720	121.980
9.00E+15	0.3	0.2	6.00E+17	0.749	152.970
9.00E+15	0.3	0.2	7.00E+17	0.758	182.464
9.00E+15	0.3	0.2	8.00E+17	0.765	217.885
9.00E+15	0.3	0.2	9.00E+17	0.772	256.333
9.00E+15	0.3	0.2	1.00E+18	0.782	299.785
9.00E+15	0.3	0.25	3.00E+17	0.725	90.377
9.00E+15	0.3	0.25	4.00E+17	0.780	120.775
9.00E+15	0.3	0.25	5.00E+17	0.805	156.481
9.00E+15	0.3	0.25	6.00E+17	0.825	197.726
9.00E+15	0.3	0.25	7.00E+17	0.836	247.610
9.00E+15	0.3	0.25	8.00E+17	0.846	292.697
9.00E+15	0.3	0.25	9.00E+17	0.854	345.956
9.00E+15	0.3	0.25	1.00E+18	0.869	395.159
9.00E+15	0.3	0.3	3.00E+17	0.801	108.612
9.00E+15	0.3	0.3	4.00E+17	0.862	149.681
9.00E+15	0.3	0.3	5.00E+17	0.887	198.301
9.00E+15	0.3	0.3	6.00E+17	0.910	250.269
9.00E+15	0.3	0.3	7.00E+17	0.925	312.508
9.00E+15	0.3	0.3	8.00E+17	0.936	366.611
9.00E+15	0.3	0.3	9.00E+17	0.945	424.653
9.00E+15	0.3	0.3	1.00E+18	0.960	487.765
9.00E+15	0.3	0.35	3.00E+17	0.883	124.260
9.00E+15	0.3	0.35	4.00E+17	0.951	175.761
9.00E+15	0.3	0.35	5.00E+17	0.978	237.417
9.00E+15	0.3	0.35	6.00E+17	1.000	308.314
9.00E+15	0.3	0.35	7.00E+17	1.018	371.318
9.00E+15	0.3	0.35	8.00E+17	1.034	443.224
9.00E+15	0.3	0.35	9.00E+17	1.048	505.634
9.00E+15	0.3	0.35	1.00E+18	1.058	577.933
9.00E+15	0.3	0.4	3.00E+17	0.968	143.117
9.00E+15	0.3	0.4	4.00E+17	1.047	210.221
9.00E+15	0.3	0.4	5.00E+17	1.079	285.215
9.00E+15	0.3	0.4	6.00E+17	1.101	361.419
9.00E+15	0.3	0.4	7.00E+17	1.120	432.861
9.00E+15	0.3	0.4	8.00E+17	1.135	513.755

9.00E+15	0.3	0.4	9.00E+17	1.160	584.491
9.00E+15	0.3	0.4	1.00E+18	1.175	665.680
9.00E+15	0.35	0.15	3.00E+17	0.605	66.053
9.00E+15	0.35	0.15	4.00E+17	0.640	81.233
9.00E+15	0.35	0.15	5.00E+17	0.681	98.960
9.00E+15	0.35	0.15	6.00E+17	0.719	119.005
9.00E+15	0.35	0.15	7.00E+17	0.735	141.241
9.00E+15	0.35	0.15	8.00E+17	0.746	165.872
9.00E+15	0.35	0.15	9.00E+17	0.755	192.634
9.00E+15	0.35	0.15	1.00E+18	0.760	221.492
9.00E+15	0.35	0.2	3.00E+17	0.688	82.245
9.00E+15	0.35	0.2	4.00E+17	0.758	107.343
9.00E+15	0.35	0.2	5.00E+17	0.780	136.787
9.00E+15	0.35	0.2	6.00E+17	0.807	170.545
9.00E+15	0.35	0.2	7.00E+17	0.821	208.432
9.00E+15	0.35	0.2	8.00E+17	0.832	250.101
9.00E+15	0.35	0.2	9.00E+17	0.843	293.480
9.00E+15	0.35	0.2	1.00E+18	0.852	336.929
9.00E+15	0.35	0.25	3.00E+17	0.797	100.111
9.00E+15	0.35	0.25	4.00E+17	0.844	135.921
9.00E+15	0.35	0.25	5.00E+17	0.876	178.389
9.00E+15	0.35	0.25	6.00E+17	0.898	227.334
9.00E+15	0.35	0.25	7.00E+17	0.911	280.705
9.00E+15	0.35	0.25	8.00E+17	0.925	334.558
9.00E+15	0.35	0.25	9.00E+17	0.946	388.468
9.00E+15	0.35	0.25	1.00E+18	0.959	442.493
9.00E+15	0.35	0.3	3.00E+17	0.881	119.698
9.00E+15	0.35	0.3	4.00E+17	0.949	167.711
9.00E+15	0.35	0.3	5.00E+17	0.983	225.074
9.00E+15	0.35	0.3	6.00E+17	0.987	288.658
9.00E+15	0.35	0.3	7.00E+17	1.009	352.755
9.00E+15	0.35	0.3	8.00E+17	1.038	416.919
9.00E+15	0.35	0.3	9.00E+17	1.052	480.992
9.00E+15	0.35	0.3	1.00E+18	1.065	545.188
9.00E+15	0.35	0.35	3.00E+17	0.983	140.877
9.00E+15	0.35	0.35	4.00E+17	1.053	202.554
9.00E+15	0.35	0.35	5.00E+17	1.094	274.796
9.00E+15	0.35	0.35	6.00E+17	1.117	348.813
9.00E+15	0.35	0.35	7.00E+17	1.134	422.635
9.00E+15	0.35	0.35	8.00E+17	1.160	496.754
9.00E+15	0.35	0.35	9.00E+17	1.174	570.865
9.00E+15	0.35	0.35	1.00E+18	1.188	644.751
9.00E+15	0.35	0.4	3.00E+17	1.090	163.849
9.00E+15	0.35	0.4	4.00E+17	1.150	240.174
9.00E+15	0.35	0.4	5.00E+17	1.208	323.860
9.00E+15	0.35	0.4	6.00E+17	1.239	407.737
9.00E+15	0.35	0.4	7.00E+17	1.272	491.533
9.00E+15	0.35	0.4	8.00E+17	1.294	575.263
9.00E+15	0.35	0.4	9.00E+17	1.320	658.979
9.00E+15	0.35	0.4	1.00E+18	1.342	742.260
9.00E+15	0.4	0.15	3.00E+17	0.626	68.933
9.00E+15	0.4	0.15	4.00E+17	0.669	85.929
9.00E+15	0.4	0.15	5.00E+17	0.728	105.926
9.00E+15	0.4	0.15	6.00E+17	0.769	128.617
9.00E+15	0.4	0.15	7.00E+17	0.790	153.951
9.00E+15	0.4	0.15	8.00E+17	0.801	181.835
9.00E+15	0.4	0.15	9.00E+17	0.808	212.392
9.00E+15	0.4	0.15	1.00E+18	0.825	244.788

9.00E+15	0.4	0.2	3.00E+17	0.734	87.164
9.00E+15	0.4	0.2	4.00E+17	0.816	115.484
9.00E+15	0.4	0.2	5.00E+17	0.856	148.790
9.00E+15	0.4	0.2	6.00E+17	0.878	187.095
9.00E+15	0.4	0.2	7.00E+17	0.901	230.238
9.00E+15	0.4	0.2	8.00E+17	0.913	276.575
9.00E+15	0.4	0.2	9.00E+17	0.925	323.272
9.00E+15	0.4	0.2	1.00E+18	0.943	369.961
9.00E+15	0.4	0.25	3.00E+17	0.866	107.508
9.00E+15	0.4	0.25	4.00E+17	0.937	148.275
9.00E+15	0.4	0.25	5.00E+17	0.970	196.804
9.00E+15	0.4	0.25	6.00E+17	0.989	252.222
9.00E+15	0.4	0.25	7.00E+17	1.012	310.404
9.00E+15	0.4	0.25	8.00E+17	1.029	368.583
9.00E+15	0.4	0.25	9.00E+17	1.050	426.761
9.00E+15	0.4	0.25	1.00E+18	1.068	484.880
9.00E+15	0.4	0.3	3.00E+17	0.986	130.024
9.00E+15	0.4	0.3	4.00E+17	1.051	184.993
9.00E+15	0.4	0.3	5.00E+17	1.092	250.322
9.00E+15	0.4	0.3	6.00E+17	1.116	319.621
9.00E+15	0.4	0.3	7.00E+17	1.142	358.271
9.00E+15	0.4	0.3	8.00E+17	1.162	458.112
9.00E+15	0.4	0.3	9.00E+17	1.172	527.532
9.00E+15	0.4	0.3	1.00E+18	1.189	596.727
9.00E+15	0.4	0.35	3.00E+17	1.095	154.750
9.00E+15	0.4	0.35	4.00E+17	1.172	225.528
9.00E+15	0.4	0.35	5.00E+17	1.215	305.252
9.00E+15	0.4	0.35	6.00E+17	1.242	385.423
9.00E+15	0.4	0.35	7.00E+17	1.275	465.648
9.00E+15	0.4	0.35	8.00E+17	1.295	545.847
9.00E+15	0.4	0.35	9.00E+17	1.321	625.906
9.00E+15	0.4	0.35	1.00E+18	1.336	705.753
9.00E+15	0.4	0.4	3.00E+17	1.226	181.572
9.00E+15	0.4	0.4	4.00E+17	1.314	268.426
9.00E+15	0.4	0.4	5.00E+17	1.366	359.196
9.00E+15	0.4	0.4	6.00E+17	1.408	450.143
9.00E+15	0.4	0.4	7.00E+17	1.438	540.943
9.00E+15	0.4	0.4	8.00E+17	1.472	631.851
9.00E+15	0.4	0.4	9.00E+17	1.496	722.111
9.00E+15	0.4	0.4	1.00E+18	1.530	812.114
9.00E+15	0.45	0.15	3.00E+17	0.649	71.983
9.00E+15	0.45	0.15	4.00E+17	0.700	91.105
9.00E+15	0.45	0.15	5.00E+17	0.775	113.386
9.00E+15	0.45	0.15	6.00E+17	0.825	138.859
9.00E+15	0.45	0.15	7.00E+17	0.858	167.391
9.00E+15	0.45	0.15	8.00E+17	0.870	198.919
9.00E+15	0.45	0.15	9.00E+17	0.880	233.305
9.00E+15	0.45	0.15	1.00E+18	0.895	269.550
9.00E+15	0.45	0.2	3.00E+17	0.783	92.596
9.00E+15	0.45	0.2	4.00E+17	0.880	124.341
9.00E+15	0.45	0.2	5.00E+17	0.907	161.935
9.00E+15	0.45	0.2	6.00E+17	0.956	205.125
9.00E+15	0.45	0.2	7.00E+17	0.971	253.435
9.00E+15	0.45	0.2	8.00E+17	0.995	303.524
9.00E+15	0.45	0.2	9.00E+17	1.017	353.544
9.00E+15	0.45	0.2	1.00E+18	1.034	403.745
9.00E+15	0.45	0.25	3.00E+17	0.926	115.547
9.00E+15	0.45	0.25	4.00E+17	1.023	161.471

9.00E+15	0.45	0.25	5.00E+17	1.057	216.197
9.00E+15	0.45	0.25	6.00E+17	1.099	277.727
9.00E+15	0.45	0.25	7.00E+17	1.107	339.992
9.00E+15	0.45	0.25	8.00E+17	1.145	402.347
9.00E+15	0.45	0.25	9.00E+17	1.167	464.719
9.00E+15	0.45	0.25	1.00E+18	1.185	527.057
9.00E+15	0.45	0.3	3.00E+17	1.068	141.192
9.00E+15	0.45	0.3	4.00E+17	1.165	203.343
9.00E+15	0.45	0.3	5.00E+17	1.206	276.013
9.00E+15	0.45	0.3	6.00E+17	1.240	350.398
9.00E+15	0.45	0.3	7.00E+17	1.291	424.667
9.00E+15	0.45	0.3	8.00E+17	1.314	499.015
9.00E+15	0.45	0.3	9.00E+17	1.331	573.394
9.00E+15	0.45	0.3	1.00E+18	1.345	647.570
9.00E+15	0.45	0.35	3.00E+17	1.215	169.268
9.00E+15	0.45	0.35	4.00E+17	1.322	249.236
9.00E+15	0.45	0.35	5.00E+17	1.393	335.258
9.00E+15	0.45	0.35	6.00E+17	1.427	421.325
9.00E+15	0.45	0.35	7.00E+17	1.474	507.446
9.00E+15	0.45	0.35	8.00E+17	1.501	593.421
9.00E+15	0.45	0.35	9.00E+17	1.523	568.886
9.00E+15	0.45	0.35	1.00E+18	1.558	765.080
9.00E+15	0.45	0.4	3.00E+17	1.383	200.031
9.00E+15	0.45	0.4	4.00E+17	1.511	295.996
9.00E+15	0.45	0.4	5.00E+17	1.602	393.670
9.00E+15	0.45	0.4	6.00E+17	1.650	491.226
9.00E+15	0.45	0.4	7.00E+17	1.684	588.903
9.00E+15	0.45	0.4	8.00E+17	1.715	686.339
9.00E+15	0.45	0.4	9.00E+17	1.776	783.745
9.00E+15	0.45	0.4	1.00E+18	1.814	878.669
9.00E+15	0.5	0.15	3.00E+17	0.670	150.519
9.00E+15	0.5	0.15	4.00E+17	0.728	180.825
9.00E+15	0.5	0.15	5.00E+17	0.825	200.088
9.00E+15	0.5	0.15	6.00E+17	0.885	219.830
9.00E+15	0.5	0.15	7.00E+17	0.928	232.942
9.00E+15	0.5	0.15	8.00E+17	0.947	249.588
9.00E+15	0.5	0.15	9.00E+17	0.966	402.380
9.00E+15	0.5	0.15	1.00E+18	0.984	446.119
9.00E+15	0.5	0.2	3.00E+17	0.826	190.383
9.00E+15	0.5	0.2	4.00E+17	0.947	246.374
9.00E+15	0.5	0.2	5.00E+17	1.025	305.903
9.00E+15	0.5	0.2	6.00E+17	1.050	367.977
9.00E+15	0.5	0.2	7.00E+17	1.084	427.655
9.00E+15	0.5	0.2	8.00E+17	1.104	487.716
9.00E+15	0.5	0.2	9.00E+17	1.128	547.439
9.00E+15	0.5	0.2	1.00E+18	1.150	607.415
9.00E+15	0.5	0.25	3.00E+17	1.009	236.246
9.00E+15	0.5	0.25	4.00E+17	1.128	308.914
9.00E+15	0.5	0.25	5.00E+17	1.185	386.394
9.00E+15	0.5	0.25	6.00E+17	1.223	461.528
9.00E+15	0.5	0.25	7.00E+17	1.263	537.128
9.00E+15	0.5	0.25	8.00E+17	1.299	611.997
9.00E+15	0.5	0.25	9.00E+17	1.322	687.745
9.00E+15	0.5	0.25	1.00E+18	1.354	762.409
9.00E+15	0.5	0.3	3.00E+17	1.205	279.639
9.00E+15	0.5	0.3	4.00E+17	1.300	307.467
9.00E+15	0.5	0.3	5.00E+17	1.385	462.531
9.00E+15	0.5	0.3	6.00E+17	1.407	552.675

9.00E+15	0.5	0.3	7.00E+17	1.470	643.275
9.00E+15	0.5	0.3	8.00E+17	1.504	733.219
9.00E+15	0.5	0.35	3.00E+17	1.372	323.862
9.00E+15	0.5	0.35	4.00E+17	1.503	430.836
9.00E+15	0.5	0.35	5.00E+17	1.613	536.210
9.00E+15	0.5	0.35	6.00E+17	1.658	641.040
9.00E+15	0.5	0.35	7.00E+17	1.710	746.073
9.00E+15	0.5	0.35	8.00E+17	1.741	849.622
9.00E+15	0.5	0.35	9.00E+17	1.778	951.377
9.00E+15	0.5	0.4	3.00E+17	1.593	369.671
9.00E+15	0.5	0.4	4.00E+17	1.744	488.852
9.00E+15	0.5	0.4	5.00E+17	1.910	608.359
9.00E+15	0.5	0.4	6.00E+17	1.942	727.699
9.00E+15	0.5	0.4	7.00E+17	2.002	845.900
9.00E+15	0.5	0.4	8.00E+17	2.047	961.737
9.00E+15	0.5	0.4	9.00E+17	2.104	1074.390
9.00E+15	0.5	0.4	1.00E+18	2.171	1178.570
9.00E+15	0.6	0.15	3.00E+17	0.717	159.669
9.00E+15	0.6	0.15	4.00E+17	0.784	197.747
9.00E+15	0.6	0.15	5.00E+17	0.932	242.243
9.00E+15	0.6	0.15	6.00E+17	1.035	289.226
9.00E+15	0.6	0.15	7.00E+17	1.106	317.337
9.00E+15	0.6	0.15	8.00E+17	1.157	339.628
9.00E+15	0.6	0.15	9.00E+17	1.184	357.172
9.00E+15	0.6	0.15	1.00E+18	1.213	372.624
9.00E+15	0.6	0.2	3.00E+17	0.949	203.220
9.00E+15	0.6	0.2	4.00E+17	1.129	265.312
9.00E+15	0.6	0.2	5.00E+17	1.263	332.161
9.00E+15	0.6	0.2	6.00E+17	1.315	396.623
9.00E+15	0.6	0.2	7.00E+17	1.359	461.493
9.00E+15	0.6	0.2	8.00E+17	1.408	525.855
9.00E+15	0.6	0.2	9.00E+17	1.452	590.593
9.00E+15	0.6	0.25	3.00E+17	1.249	252.651
9.00E+15	0.6	0.25	4.00E+17	1.420	336.473
9.00E+15	0.6	0.25	5.00E+17	1.563	417.657
9.00E+15	0.6	0.25	6.00E+17	1.586	499.249
9.00E+15	0.6	0.25	7.00E+17	1.658	580.857
9.00E+15	0.6	0.25	8.00E+17	1.727	662.479
9.00E+15	0.6	0.25	9.00E+17	1.765	743.940
9.00E+15	0.6	0.25	1.00E+18	1.801	824.596
9.00E+15	0.6	0.3	3.00E+17	1.534	303.272
9.00E+15	0.6	0.3	4.00E+17	1.717	403.384
9.00E+15	0.6	0.3	5.00E+17	1.878	501.664
9.00E+15	0.6	0.3	6.00E+17	1.947	507.207
9.00E+15	0.6	0.3	7.00E+17	2.036	697.916
9.00E+15	0.6	0.3	8.00E+17	2.099	795.282
9.00E+15	0.6	0.3	9.00E+17	2.144	892.508
9.00E+15	0.6	0.3	1.00E+18	2.180	986.931
9.00E+15	0.6	0.35	3.00E+17	1.845	354.686
9.00E+15	0.6	0.35	4.00E+17	2.075	468.652
9.00E+15	0.6	0.35	5.00E+17	2.372	582.929
9.00E+15	0.6	0.35	6.00E+17	2.401	697.271
9.00E+15	0.6	0.35	8.00E+17	2.570	924.294
9.00E+15	0.6	0.35	9.00E+17	2.660	1033.760
9.00E+15	0.6	0.35	1.00E+18	2.753	1139.480
9.00E+15	0.6	0.4	3.00E+17	2.225	402.986
9.00E+15	0.6	0.4	4.00E+17	2.531	533.182
9.00E+15	0.6	0.4	5.00E+17	2.925	663.342

9.00E+15	0.6	0.4	6.00E+17	2.981	793.437
9.00E+15	0.6	0.4	7.00E+17	3.110	922.080
9.00E+15	0.6	0.4	9.00E+17	3.391	1168.330
9.00E+15	0.6	0.4	1.00E+18	3.499	1277.210
