# Supplementary material for: GaN JBS Diode Device Performance Prediction Method Based on Neural Network
Source: Micromachines (Basel). 2023 Jan 12;14(1):188. doi: 10.3390/mi14010188 (PMC9860762; doi:10.3390/mi14010188)
Supplement: Supplementary file 1 [file micromachines-14-00188-s001.zip › Predicted results.pdf]

	Prediction results						
	Ron				BV		
	TRUE	predicted	error		TRUE	predicted	error
1	0.982	0.991101	0.009268		335.929	329.0503	0.020477
2	0.539	0.541153	0.003995		247.702	240.6711	0.028384
3	0.747	0.739867	0.009549		221.04	211.1457	0.044762
4	0.438	0.436088	0.004365		385.103	380.0013	0.013248
5	0.776	0.762051	0.017975		111.348	110.6059	0.006664
6	0.5	0.527214	0.054428		86.6467	91.28741	0.053559
7	1.096	1.075516	0.01869		198.981	190.4914	0.042666
8	0.316	0.316491	0.001553		178.856	168.5196	0.057792
9	1.42	1.396853	0.016301		336.473	342.4634	0.017803
10	1.142	1.112745	0.025617		358.271	364.0105	0.01602
11	1.05	1.105678	0.053026		367.977	363.3814	0.012489
12	0.821	0.823571	0.003131		208.432	195.4053	0.062498
13	2.026	2.056604	0.015106		1071.6	1060.939	0.009949
14	1.299	1.29124	0.005974		611.997	594.698	0.028266
15	0.513	0.495621	0.033878		198.896	200.8339	0.009743
16	0.906	0.895678	0.011392		321.498	321.493	1.55E-05
17	0.747	0.717107	0.040018		221.04	212.7891	0.037328
18	0.551	0.532841	0.032956		148.468	157.046	0.057777
19	0.717	0.703959	0.018189		221.396	228.315	0.031251
20	2.03	2.011676	0.009027		681.135	690.4211	0.013633
21	0.318	0.332507	0.045621		78.0168	74.09206	0.050306
22	0.702	0.731772	0.04241		216.327	201.1966	0.069942
23	0.556	0.537228	0.033762		203.169	191.4513	0.057675
24	0.896	0.926409	0.033939		287.248	287.9042	0.002284
25	0.502	0.516075	0.028037		257.921	278.5673	0.080049
26	0.475	0.477707	0.005699		211.29	202.5327	0.041447
27	0.895	0.922849	0.031116		269.55	268.6092	0.00349
28	0.669	0.66718	0.002721		85.9292	83.98791	0.022592
29	0.306	0.303162	0.009273		111.041	120.5439	0.08558
30	0.793	0.793775	0.000977		110.153	110.2959	0.001297
31	0.719	0.704152	0.020651		107.91	104.5833	0.030828
32	1.832	1.830312	0.000921		755.372	751.0768	0.005686
33	0.938	0.929601	0.008955		275.115	265.4953	0.034966
34	0.677	0.691761	0.021803		107.805	100.8415	0.064594
35	0.384	0.371729	0.031957		77.2118	82.76406	0.071909
36	1.18	1.176413	0.00304		186.985	185.0963	0.010101
37	2.047	2.037912	0.00444		961.737	906.3742	0.057565
38	0.818	0.781019	0.045209		215.036	227.3681	0.057349
39	1.087	1.144483	0.052882		250.644	237.042	0.054268
40	0.951	0.94314	0.008265		153.125	156.6104	0.022762
41	0.679	0.669114	0.01456		340.709	329.5823	0.032657
42	0.272	0.276	0.014706		116.316	108.1229	0.070438
43	0.451	0.432176	0.041738		141.631	132.1104	0.067221
44	1.144	1.128335	0.013693		457.891	491.1569	0.07265
45	1.32	1.353126	0.025095		658.979	654.9949	0.006046
46	0.41	0.430081	0.048977		101.292	111.6689	0.102445
47	0.648	0.636088	0.018382		194.331	195.4488	0.005752
48	0.447	0.478278	0.069972		142.547	138.6869	0.027079
49	1.092	1.118242	0.024031		250.322	235.3886	0.059657
50	1.878	1.818616	0.031621		501.664	501.8493	0.000369
51	0.348	0.350308	0.006633		88.8534	97.8743	0.101526
52	0.559	0.559691	0.001237		127.31	134.4604	0.056166
53	0.61	0.61719	0.011786		114.085	113.5053	0.005081
54	0.926	0.897504	0.030774		532.91	522.3433	0.019828
55	1.366	1.384329	0.013418		359.196	343.0878	0.044845

56	0.989	0.999447	0.010563	252.222	242.0362	0.040384
57	1.142	1.123847	0.015896	370.712	343.6704	0.072945
58	1.048	1.044204	0.003622	505.634	483.9161	0.042952
59	0.601	0.60219	0.00198	489.375	542.1236	0.107788
60	0.256	0.247946	0.031462	61.4453	57.70615	0.060853
61	0.853	0.859534	0.00766	346.053	380.3684	0.099162
62	0.627	0.620651	0.010127	222.422	203.6781	0.084272
63	0.745	0.721273	0.031848	393.27	383.7086	0.024313
64	0.58	0.580503	0.000867	329.714	330.6343	0.002791
65	0.444	0.442971	0.002318	88.111	87.8123	0.00339
66	0.531	0.551824	0.039217	192.723	184.7351	0.041447
67	1.65	1.664162	0.008583	491.226	535.4684	0.090065
68	0.414	0.413467	0.001287	96.6266	95.31779	0.013545
69	0.654	0.637718	0.024896	323.673	321.521	0.006649
70	0.496	0.50629	0.020746	87.6305	90.11517	0.028354
71	2.595	2.577291	0.006824	550.674	556.7103	0.010962
72	0.26	0.264041	0.015541	53.2154	50.39805	0.052942
73	0.777	0.731348	0.058754	366.592	328.8638	0.102916
74	0.404	0.403088	0.002258	77.8123	78.41689	0.00777
75	0.709	0.740604	0.044576	256.036	229.028	0.105485
76	1.113	1.106477	0.005861	244.65	242.722	0.007881
77	0.608	0.611719	0.006117	212.227	216.8915	0.021979
78	0.284	0.277793	0.021854	161.505	171.3843	0.06117
79	0.673	0.675789	0.004144	109.126	106.0045	0.028605
80	1.269	1.291716	0.0179	438.462	435.4919	0.006774
81	0.757	0.752101	0.006472	198.814	182.6736	0.081184
82	0.663	0.645054	0.027068	117.952	112.1975	0.048787
83	0.956	0.94917	0.007145	146.563	151.4302	0.033209
84	0.321	0.30158	0.060499	105.638	95.07143	0.100026
85	0.755	0.777012	0.029155	192.634	189.0211	0.018755
86	1.295	1.296039	0.000802	545.847	536.5557	0.017022
87	0.97	0.95256	0.017979	512.51	515.3209	0.005485
88	1.408	1.378986	0.020607	525.855	490.6813	0.066889
89	1.208	1.221864	0.011477	323.86	304.8515	0.058694
90	0.569	0.564966	0.007089	134.792	126.467	0.061762
91	1.01	0.979843	0.029859	443.492	439.7564	0.008423
92	1	1.017621	0.017621	308.314	302.317	0.019451
93	0.568	0.544431	0.041494	95.4735	99.37251	0.040839
94	1.162	1.142526	0.016759	458.112	454.9024	0.007006
95	1.362	1.379912	0.013152	285.067	283.1441	0.006745
96	0.339	0.364412	0.074962	141.18	150.5925	0.06667
97	0.723	0.679371	0.060345	352.924	382.8698	0.084851
98	0.825	0.839891	0.018049	223.205	218.9689	0.018979
99	0.738	0.719441	0.025148	274.31	264.194	0.036878
100	0.507	0.520194	0.026024	63.2509	62.86145	0.006157
101	0.548	0.529367	0.034003	113.87	110.411	0.030377
102	0.749	0.700811	0.064338	152.97	165.8765	0.084373
103	1.606	1.672401	0.041345	913.031	875.3751	0.041243
104	0.858	0.852108	0.006867	167.391	181.4247	0.083838
105	1.612	1.611893	6.63E-05	289.477	288.2725	0.004161
106	0.487	0.471906	0.030994	173.352	187.2315	0.080065
107	0.635	0.625434	0.015065	379.63	375.8858	0.009863
108	0.686	0.638247	0.06961	133.015	147.1053	0.10593
109	0.716	0.672972	0.060094	556.931	559.6444	0.004872
110	0.78	0.756953	0.029547	270.123	289.5966	0.072091
111	0.5	0.522991	0.045982	218.292	210.6914	0.034818
112	0.612	0.597734	0.02331	259.932	262.6955	0.010632
113	1.012	1.012968	0.000956	310.404	291.9817	0.059349

114	0.813	0.78913	0.02936	318.769	300.0322	0.058779
115	0.952	0.945237	0.007104	235.278	240.0574	0.020314
116	0.609	0.587073	0.036005	112.459	116.8069	0.038662
117	0.698	0.690898	0.010175	158.345	144.7833	0.085647
118	1.017	0.992783	0.023813	353.544	383.0058	0.083333
119	1.409	1.464744	0.039563	308.111	337.105	0.094102
120	0.287	0.286299	0.002441	61.555	60.54248	0.016449
121	0.464	0.47883	0.031961	185.656	179.8557	0.031242
122	0.945	0.93176	0.01401	424.653	409.5806	0.035493
123	1.029	1.033813	0.004677	368.583	375.2997	0.018223
124	0.451	0.421668	0.065037	87.1887	92.17513	0.057191
125	1.277	1.235729	0.032318	404.125	412.3827	0.020434
126	0.71	0.722487	0.017587	181.604	182.4236	0.004513
127	0.807	0.819986	0.016091	87.681	88.84405	0.013265
128	0.931	0.923793	0.007741	441.562	448.2144	0.015066
129	0.44	0.360994	0.17956	343.177	320.1602	0.06707
130	0.316	0.316935	0.00296	82.538	89.76399	0.087547
131	0.719	0.743496	0.034069	338.464	318.7615	0.058212
132	0.428	0.423741	0.009952	77.7334	78.54618	0.010456
133	0.336	0.335599	0.001194	159.107	166.3184	0.045324
134	1.004	1.053743	0.049545	599.451	612.535	0.021827
135	0.651	0.600103	0.078182	322.079	355.4142	0.1035
136	0.989	1.023651	0.035037	634.91	600.184	0.054694
137	0.916	0.903024	0.014166	133.121	134.8723	0.013156
138	0.765	0.754399	0.013858	217.885	221.3864	0.01607
139	0.412	0.402751	0.022448	127.565	123.1546	0.034574
140	1.012	1.027016	0.014838	310.404	321.1942	0.034762
141	0.352	0.351458	0.001541	145.224	143.2888	0.013326
142	0.488	0.461697	0.0539	222.128	223.1132	0.004435
143	0.524	0.4916	0.061832	113.544	119.1703	0.049552
144	0.279	0.260673	0.065689	124.203	114.7369	0.076215
145	0.866	0.903502	0.043305	107.508	113.5575	0.05627
146	0.321	0.325788	0.014916	86.3452	88.67542	0.026987
147	0.833	0.805997	0.032416	318.115	318.0478	0.000211
148	0.507	0.528893	0.043182	107.279	114.4328	0.066684
149	0.381	0.351113	0.078444	127.742	116.3293	0.089342
150	1.586	1.612086	0.016447	499.249	489.5958	0.019335
151	0.783	0.770052	0.016536	129.468	126.1646	0.025515
152	0.767	0.708115	0.076773	210.508	217.9664	0.03543
153	2.217	2.149278	0.030547	443.064	516.9444	0.166749
154	0.316	0.40572	0.283924	100.773	106.8852	0.060653
155	0.538	0.512529	0.047345	203.05	209.8965	0.033718
156	0.601	0.577309	0.039419	245.685	239.3721	0.025695
157	0.932	0.853252	0.084494	242.243	238.8225	0.01412
158	0.383	0.363163	0.051794	97.5466	102.807	0.053927
159	0.599	0.611131	0.020252	196.462	216.1033	0.099975
160	0.854	0.831292	0.02659	345.956	327.0743	0.054578
161	0.462	0.453116	0.019231	173.488	171.0608	0.013991
162	0.436	0.42971	0.014426	162.509	168.1759	0.034872
163	1.129	1.095758	0.029444	265.312	258.0328	0.027436
164	0.382	0.368707	0.034798	50.064	52.54807	0.049618
165	1.284	1.310078	0.02031	253.156	254.8325	0.006622
166	0.966	0.932538	0.034639	402.38	416.1893	0.034319
167	1.176	1.217913	0.035641	605.547	591.453	0.023275
168	0.34	0.342867	0.008434	56.5033	56.6061	0.001819
169	0.412	0.43216	0.048933	138.144	131.2061	0.050222
170	0.733	0.682092	0.069452	424.523	462.6449	0.089799
171	0.616	0.589136	0.04361	318.669	327.0305	0.026239

172	1.083	1.040718	0.039042	541.134	543.3161	0.004033
173	0.984	0.931983	0.052863	446.119	448.9272	0.006295
174	1.387	1.339953	0.03392	777.288	782.3271	0.006483
175	0.394	0.378943	0.038216	171.082	173.1663	0.012183
176	0.848	0.87448	0.031226	311.712	311.6346	0.000248
177	0.332	0.338794	0.020463	112.529	114.9439	0.02146
178	2.228	2.339775	0.050169	630.187	630.4189	0.000368
179	0.7	0.71388	0.019829	96.8939	92.02273	0.050273
180	2.422	2.459667	0.015552	537.741	524.1777	0.025223
181	1.172	1.192062	0.017118	527.532	507.6482	0.037692
182	0.587	0.584991	0.003423	146.814	153.8895	0.048194
183	0.323	0.323812	0.002513	132.685	130.6396	0.015415
184	0.541	0.563699	0.041958	88.0598	91.69212	0.041248
185	0.364	0.386188	0.060955	158.038	158.4926	0.002876
186	0.947	0.942512	0.004739	231.054	259.4365	0.122839
187	0.395	0.392095	0.007356	117.507	120.3733	0.024393
188	0.7	0.704817	0.006881	96.8939	107.3624	0.108041
189	0.9	0.91813	0.020145	294.188	290.6811	0.011921
190	0.721	0.71909	0.002649	324.742	306.3946	0.056498
191	0.37	0.375455	0.014743	120.779	118.0592	0.022519
192	0.49	0.488069	0.003941	329.108	325.3888	0.011301
193	0.917	0.875273	0.045503	323.513	362.4716	0.120423
194	1.175	1.258508	0.07107	665.68	664.4942	0.001781
195	0.648	0.656768	0.013531	178.86	174.9514	0.021853
196	0.388	0.376783	0.028909	82.7442	87.4276	0.056601
197	0.483	0.472269	0.022217	165.895	164.1468	0.010538
198	0.563	0.554967	0.014268	164.758	164.9511	0.001172
199	0.547	0.555209	0.015007	214.889	204.7491	0.047187
200	0.732	0.740774	0.011986	133.561	141.5016	0.059453
201	1.27	1.257184	0.010091	441.902	483.494	0.09412
202	0.623	0.627802	0.007707	70.2197	70.01908	0.002857
203	0.689	0.66092	0.040755	398.774	454.2701	0.139167
204	1.354	1.401325	0.034952	762.409	671.5208	0.119212
205	1.824	1.816699	0.004003	696.195	682.6971	0.019388
206	1.315	1.362565	0.036171	396.623	374.5396	0.055679
207	0.716	0.680681	0.049328	255.979	249.2037	0.026468
208	0.675	0.678333	0.004938	227.145	204.7477	0.098604
209	0.609	0.615774	0.011123	292.998	286.5457	0.022022
210	1.034	1.000981	0.031933	443.224	413.5657	0.066915
211	1.08	1.092844	0.011893	213.162	207.8467	0.024936
212	0.592	0.589996	0.003385	69.58	69.3325	0.003557
213	0.578	0.566192	0.02043	158.683	146.5773	0.076289
214	1.096	1.108644	0.011536	480.139	486.8732	0.014025
215	0.453	0.471767	0.041429	107.311	104.9778	0.021743
216	1.117	1.16016	0.038639	348.813	339.1931	0.027579
217	0.852	0.853357	0.001592	145.013	135.8397	0.063258
218	1.135	1.113764	0.01871	513.755	499.4235	0.027896
219	0.869	0.817103	0.059721	395.159	362.4499	0.082774
220	1.011	1.090495	0.07863	246.624	242.3983	0.017134
221	0.906	0.878354	0.030514	434.941	404.3824	0.070259
222	1.045	0.9647	0.076842	247.581	257.1786	0.038765
223	0.376	0.373008	0.007959	90.4016	100.0554	0.106788
224	1.697	1.773463	0.045058	586.565	564.0344	0.038411
225	0.607	0.61458	0.012488	237.546	234.9076	0.011107
226	0.432	0.418392	0.031499	246.982	311.4021	0.260829
227	0.922	0.939217	0.018674	302.898	327.9628	0.08275
228	1.952	1.9918	0.020389	950.723	947.9894	0.002875
229	0.93	0.871091	0.063343	561.655	493.637	0.121103

230	0.721	0.715961	0.006989	341.292	336.3691	0.014424
231	0.728	0.710431	0.024133	235.618	219.52	0.068323
232	0.275	0.283949	0.032542	65.8019	73.12288	0.111258
233	0.604	0.571594	0.053652	208.957	191.9485	0.081397
234	1.066	1.047871	0.017006	148.97	145.51	0.023226
235	0.31	0.307457	0.008202	108.565	105.9055	0.024497
236	0.822	0.771191	0.061811	530.941	588.1975	0.10784
237	0.911	0.93377	0.024994	280.705	268.3181	0.044128
238	0.555	0.538098	0.030454	138.634	137.8673	0.005531
239	0.688	0.704683	0.024249	288.938	311.112	0.076743
240	0.409	0.414216	0.012753	109.095	113.6272	0.041544
241	0.908	0.867795	0.044278	354.881	365.5707	0.030122
242	1.068	1.045814	0.020774	484.88	466.07	0.038793
243	2.002	2.134664	0.066266	845.9	810.3061	0.042078
244	1.587	1.551478	0.022383	713.622	711.2258	0.003358
245	0.878	0.899183	0.024127	208.305	194.9289	0.064214
246	0.274	0.290593	0.06056	101.25	105.033	0.037363
247	0.907	0.877075	0.032994	161.935	204.0932	0.26034
248	1.257	1.25424	0.002196	297.789	252.8915	0.15077
249	0.469	0.478805	0.020905	153.452	154.9048	0.009468
250	0.637	0.63522	0.002794	281.652	269.8804	0.041795
251	1.626	1.615985	0.006159	826.565	823.6522	0.003524
252	0.553	0.539434	0.024531	198.285	212.857	0.07349
253	2.068	2.05422	0.006663	771.649	780.9459	0.012048
254	0.661	0.650052	0.016562	88.0801	101.5842	0.153316
255	1.806	1.803125	0.001592	675.456	671.6174	0.005683
256	1.16	1.153792	0.005352	496.754	488.9586	0.015693
257	0.502	0.505972	0.007913	101.488	113.4635	0.117999
258	0.562	0.581285	0.034315	412.303	385.7804	0.064328
259	0.785	0.73345	0.065669	340.734	324.7147	0.047014
260	0.758	0.727329	0.040463	394.645	447.4992	0.133928
261	0.61	0.623384	0.021941	351.184	339.3716	0.033636
262	1.142	1.149233	0.006334	358.271	378.3229	0.055969
263	1.06	1.075248	0.014385	347.241	344.1339	0.008948
264	0.888	0.873594	0.016222	371.33	352.259	0.051359
265	0.496	0.488973	0.014168	186.123	191.3002	0.027816
266	0.794	0.796462	0.003101	319.295	334.1233	0.046441
267	0.348	0.319687	0.08136	127.196	111.9376	0.119959
268	0.96	0.969475	0.00987	264.7	240.1742	0.092655
269	0.24	0.22678	0.055082	49.4016	49.33638	0.00132
270	1.407	1.48461	0.05516	552.675	497.9924	0.098942
271	0.453	0.444297	0.019213	204.955	187.124	0.086999
272	2.134	2.240088	0.049713	549.008	548.1796	0.001509
273	0.394	0.40797	0.035458	106.756	148.9021	0.394789
274	0.361	0.36297	0.005458	180.406	192.2105	0.065433
275	0.41	0.418413	0.020519	124.823	112.9078	0.095457
276	1.715	1.741948	0.015713	686.339	728.7927	0.061855
277	0.656	0.626488	0.044988	148.666	168.1995	0.131392
278	0.363	0.437996	0.2066	78.3514	83.49652	0.065667
279	1.273	1.278605	0.004403	486.922	458.0372	0.059321
280	0.615	0.62109	0.009903	292.744	258.1221	0.118267
281	0.355	0.348621	0.01797	60.4205	56.88576	0.058502
282	1.015	1.023179	0.008058	214.504	224.3645	0.045969
283	0.475	0.468549	0.013581	165.241	157.4185	0.04734
284	0.617	0.596552	0.033142	259.502	240.3746	0.073708
285	1.421	1.41831	0.001893	530.862	518.3175	0.023631
286	1.344	1.319484	0.018241	286.808	276.9933	0.034221
287	0.641	0.643427	0.003786	141.593	131.4296	0.071779

288	0.343	0.329775	0.038556		92.0634	91.47127	0.006432
289	0.44	0.450358	0.02354		182.228	184.3426	0.011604
290	0.296	0.304168	0.027596		87.3965	88.03594	0.007317
291	0.489	0.490869	0.003822		107.083	98.38892	0.08119
292	0.341	0.359395	0.053943		87.3249	90.59796	0.037481
293	1.503	1.522344	0.01287		430.836	379.9136	0.118195
294	0.49	0.514808	0.050628		60.7396	70.06403	0.153515
295	0.459	0.473373	0.031314		99.7613	101.011	0.012527
296	0.613	0.601117	0.019386		96.8439	94.4841	0.024367
297	0.571	0.584091	0.022927		82.6309	79.94245	0.032536
298	0.618	0.593262	0.040029		344.444	349.6313	0.01506
299	0.66	0.652423	0.01148		291.124	250.6137	0.139151
300	0.358	0.402034	0.122999		91.8724	94.10093	0.024257
301	1.05	1.013944	0.034339		523.825	554.6803	0.058904
302	0.856	0.866244	0.011968		148.79	141.233	0.050789
			**0.02776**				**0.04769**
