# Supplementary material for: GaN JBS Diode Device Performance Prediction Method Based on Neural Network
Source: Micromachines (Basel). 2023 Jan 12;14(1):188. doi: 10.3390/mi14010188 (PMC9860762; doi:10.3390/mi14010188)
Supplement: Supplementary file 1 [file micromachines-14-00188-s001.zip › Sim_GaN JBS.pdf]

Device simulation results					
Epidop	L	Impthickness	Impdop	Ron	BV
Impdop					
5.00E+15	0.4	0.4	1.00E+17	0.67	57.86
5.00E+15	0.4	0.4	2.00E+17	0.85	97.64
5.00E+15	0.4	0.4	3.00E+17	0.95	153.13
5.00E+15	0.4	0.4	4.00E+17	1.02	224.20
5.00E+15	0.4	0.4	5.00E+17	1.06	309.89
5.00E+15	0.4	0.4	6.00E+17	1.09	400.67
5.00E+15	0.4	0.4	7.00E+17	1.12	491.48
5.00E+15	0.4	0.4	8.00E+17	1.14	582.33
5.00E+15	0.4	0.4	9.00E+17	1.16	672.90
5.00E+15	0.4	0.4	1.00E+18	1.18	762.85
5.00E+15	0.4	0.4	1.50E+18	1.25	920.26
5.00E+15	0.4	0.4	2.00E+18	1.33	1005.72
5.00E+15	0.4	0.4	3.00E+18	1.37	1070.99
5.00E+15	0.4	0.4	4.00E+18	1.43	1071.11
5.00E+15	0.4	0.4	5.00E+18	1.49	1074.42
Epidop					
1.00E+15	0.4	0.4	8.00E+17	7.28	780.08
2.00E+15	0.4	0.4	8.00E+17	3.22	730.54
3.00E+15	0.4	0.4	8.00E+17	2.03	681.14
4.00E+15	0.4	0.4	8.00E+17	1.47	631.85
5.00E+15	0.4	0.4	8.00E+17	1.14	582.33
6.00E+15	0.4	0.4	8.00E+17	0.93	532.91
7.00E+15	0.4	0.4	8.00E+17	0.77	483.38
8.00E+15	0.4	0.4	8.00E+17	0.65	435.05
9.00E+15	0.4	0.4	8.00E+17	0.56	394.37
1.00E+16	0.4	0.4	8.00E+17	0.50	362.50
Impthickness					
5.00E+15	0.4	0.1	8.00E+17	0.52	95.54
5.00E+15	0.4	0.15	8.00E+17	0.64	154.34
5.00E+15	0.4	0.2	8.00E+17	0.73	231.77
5.00E+15	0.4	0.25	8.00E+17	0.81	318.77
5.00E+15	0.4	0.3	8.00E+17	0.91	346.26
5.00E+15	0.4	0.35	8.00E+17	1.02	496.20
5.00E+15	0.4	0.4	8.00E+17	1.14	582.33
5.00E+15	0.4	0.45	8.00E+17	1.28	666.52
5.00E+15	0.4	0.5	8.00E+17	1.46	749.92
5.00E+15	0.4	0.55	8.00E+17	1.62	832.67
5.00E+15	0.4	0.6	8.00E+17	1.90	913.55
L					
5.00E+15	0.15	0.4	8.00E+17	0.62	304.52
5.00E+15	0.2	0.4	8.00E+17	0.69	385.08
5.00E+15	0.25	0.4	8.00E+17	0.77	378.29
5.00E+15	0.3	0.4	8.00E+17	0.88	463.93
5.00E+15	0.35	0.4	8.00E+17	1.00	525.65
5.00E+15	0.4	0.4	8.00E+17	1.14	582.33
5.00E+15	0.45	0.4	8.00E+17	1.35	637.05
5.00E+15	0.5	0.4	8.00E+17	1.61	913.03
5.00E+15	0.55	0.4	8.00E+17	1.92	957.59
5.00E+15	0.6	0.4	8.00E+17	2.43	999.29
5.00E+15	0.65	0.4	8.00E+17	3.39	1039.01
5.00E+15	0.7	0.4	8.00E+17	5.92	1077.01
